# Supplementary material for: Genome-Wide Identification and Characterization of SPL Family Genes in Chenopodium quinoa
Source: Genes (Basel). 2022 Aug 16;13(8):1455. doi: 10.3390/genes13081455 (PMC9408038; doi:10.3390/genes13081455)
Supplement: Supplementary file 1 [file genes-13-01455-s001.zip › Figure S.pdf]

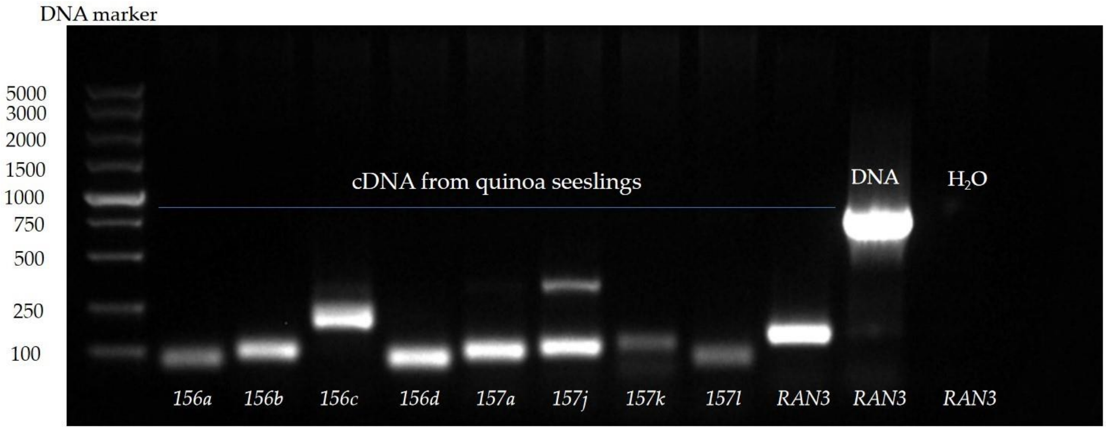

Figure S1. Expression of several *Cqu-MIR156/7* loci in quinoa seedlings. A short name for the *Cqu-MIR156/7* was added below each band, for example, *156a* indicating *Cqu-MIR156a.* The templates used for PCR were indicated on the top of the bands. An unspecific band (the weak band) was appeared from PCR for *Cqu-MIR157j.* The primers used for the PCR were listed in Table S2.

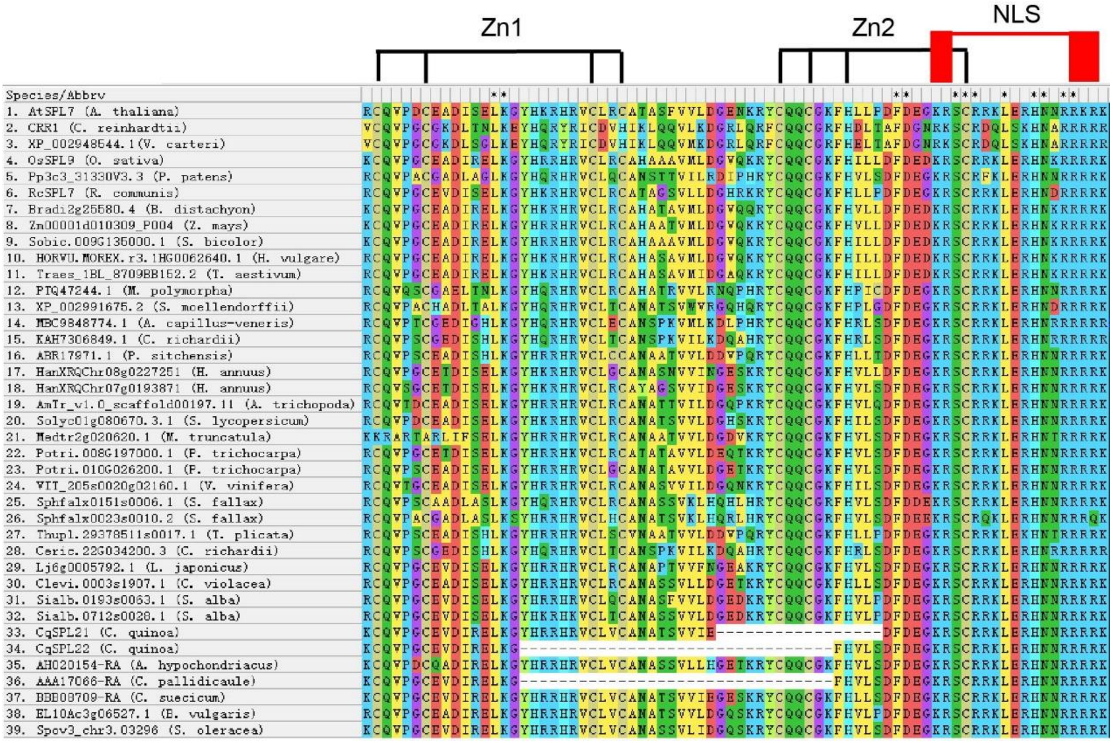

Figure S2. Multiple sequence alignment of the SBP-domains within the AtSPL7 orthologs from different species (Supplementary file 5). Except for the CqSPL21, CqSPL22 and AAA17066-RA (*C. pallidicaule*) missing part of sequences in their SBP-domains, others contain typical SBP-domain of 76 amino acids. The two conserved zinc fingers (Zn1 and Zn2) and the nuclear localization signal (NLS) were indicated.
