# Supplementary material for: Genome-Wide Identification and Characterization of SPL Family Genes in Chenopodium quinoa
Source: Genes (Basel). 2022 Aug 16;13(8):1455. doi: 10.3390/genes13081455 (PMC9408038; doi:10.3390/genes13081455)
Supplement: Supplementary file 1 [file genes-13-01455-s001.zip › Supplementary files.pdf]

Supplementary Files S1

***CqSPL* CDS and deduced protein**

>*CqSPL1*　AUR62028919-RA　1389 bp

ATGAGTGCGATTTCGATGATGGAATTGAATGCAAAAAGTCCCTTCCTGTGGGACTGGG
AGAACTTAGTCATGTTTAACACAAAAGCAGCTGAAACACCGAAAAAATTGCAGTCGG
ATTCTGAAATTGAGGGAATAGAAGGATTTGAGGCCGGGTCTTTCTATTCCTCTGGTGGT
CGTAGTGATGGTGGTACGGGTTCTGATCTGGGATATGCTTCCTCGTCGAAGAGCTCAA
AATCAGCCTCTGTTGATTCATCCGCAGACAGAGAGTTAAAAGGAAACCAATTTTCATT
GGAGGCAACCGATTGTTTCCAACAGGATCTTAGAGGCAAGAGAGATTTGGTTAATAGT
TCAGGGACAACAAGAGCATCACCAACAATTGAAAGCTCAGTGGCTTCTGGTGAATCT
TTGATTGCCTTGGAACTTGGGAAGCGGACATACTTTGAAGATGTTTCTGGGGCCAGCA
ATGTGAAGAATACTGCTCGGAGCTCTATTCCACCTGATTCTGCTGCTAAGAAAACCAA
ATCTTCATCACCCAGCATAGAAAATCCGCGTTGCCAAGTTGAAGGATGCAACCTTGAT
CTTTCCTCTGCTAAAGATTATCACCGGAAGCATAGAGTCTGTGAAAATCATTCTAAAAG
CCCAAAGGTTGTTATAAACGGACTCGAGCGAAGGTTTTGTCAACAGTGTAGCCGGTTC
CATGGCCTTTCGGAGTTTGACCAAAAGAAGAGAAGCTGTCGCAGGCGTCTTTCTGATC
ACAATGCACGTCGCCGGAAGCCAAAGCCAGAGGTCATTCAATTCAACTCGATGAGGC
TGACATCCTCCTTATATGAGGGTAGGAACCAGCTGAATTTTGCTTTTGACCAGGTACCA
ATGCATCAGACTAGGCATGGTACCTGGGATAGTGCAAGAGAATCTAAGGTTACACTGG
CAAAACCGGGATTTTTTACTACCATGAAATCCGGGGGTACTGATCAGCTGCATTCAAC
CCACAGTGGTCTGCCAAGCCCGACAACCAAAGTCTCTCCTGTTTCGACCCAACTATTG
CCATCCAAGGGTGTGCCGTCTGATGTCTTCCAGCGAGGTTTACCAGTCTCTGTGGCAC
CATCCAACTTGGCTGCAGCTCAAGATTTCCGTGCTCTCTCTCTTCTGTCAACAAGTTCT
TGGAGTTCATGCGACCCAGAACCTCCCGCACTTAACCATTCTATGCATACAAATCATGC
CTCCTTGGCTCCGTCTTTGCCTCCCATGCATCCATTGCCTCTAGGTGGCCCTCCCATATC
ATCGGAGTTCTGGCAAGCTGATCAACCAGCCTCAGGTGGTCCTGGATCTCACACCTCG
ACTTTCCAGTTGTTCAAAGCACCGAACGAGTCCAGCTTCTATCTTTACTAA

>CqSPL1 AUR62028919-RA　462

MSAISMMELNAKSPFLWDWENLVMFNTKAAETPKKLQSDSEIEGIEGFEAGSFYSSGGRSD
GGTGSDLGYASSSKSSKSASVDSSADRELKGNQFSLEATDCFQQDLRGKRDLVNSSGTTRAS
PTIESSVASGESLIALELGKRTYFEDVSGASNVKNTARSSIPPDSAAKKTKSSSPSIENPRCQVE
GCNLDLSSAKDYHRKHRVCENHSKSPKVVINGLERRFCQQCSRFHGLSEFDQKKRSCRRRL
SDHNARRRKPKPEVIQFNSMRLTSSLYEGRNQLNFAFDQVPMHQTRHGTWDSARESKVTL
AKPGFFTTMKSGGTDQLHSTHSGLPSPTTKVSPVSTQLLPSKGVPSDVFQRGLPVSVAPSNL
AAAQDFRALSLLSTSSWSSCDPEPPALNHSMHTNHASLAPSLPPMHPLPLGGPPISSEFWQ
ADQPASGGPGSHTSTFQLFKAPNESSFYLY*

>*CqSPL2* AUR62005629-RA　1389bp

ATGAGTGCGATTTCGATGATGGAATTGAATGCAAAAAGTCCCTTCCTGTGGGACTGGG
AGAACTTAGTCATGTTTAACACAAAAGCAGCTGAAACACCAAAAAAAATTGCAGTCAG
ACTCTGAAATCGAGGGAATAGAAGGATTTGAAGTCGGGTCTTTCTATTCTTCTGGTGGT
CGTAGTGATGGTGGTACGGGTTCTGATCTGGGATATGTTTCCTCGTCGAAGAGCTCAAA
ATCAGCTTCTGTTGATTCATCCGCAGACAGAGAGTTCAAAGGAAACCAAATTTCATTG

GAGGCAACCGATTGTTTCCAACAGGATCTTAGAGGCCAGAGAGATTTGGTTAATACTT
CAGGGACAACAAGAGCATCACCAACAATTGAAAGCTCTGTGGCTTCTGGTGAATCTTT
GATTGCCTTGGAACTTGGGAAGCGGACATACTTTGAAGATGTTTCTGGGGCCAGCAAT
GTGAAGAATACTGCTCGGAGCTCTATTCCACCTGATTCTGTTGCTAAGAAGACCAAAT
CTTCATCACCCAGTATAGAAAATCCGCGTTGCCAAGTTGAAGGATGCAACCTTGATCT
TTCCTCTGCTAAAGATTATCACCGGAAGCATAGAGTCTGTGAAAATCATTCTAAAAGC
CCAAAGGTTGTTATAAACGGACTCGAGCGAAGGTTTTGTCAACAGTGTAGCCGGTTCC
ATAGCCTTTCGGAGTTTGACCAAAAGAAGAGAAGCTGTCGCAGGCGTCTTTCTGATCA
CAATGCACGTCGCCGGAAGCCAAAGCCAGAGGTCATCCAATTCAACTCAATGAGGCT
GACATCCTCCTTATATGAGGGTAGGAACCAGCTGAATTTTGCTTTTGACCAGGTACCAA
TGCATCAGACTGGGCATGGTACCTGGGATAGTGCAAGAGAATCCAAGGTTACGCTGGT
GAAACCGGGATTTTTTACTACCATGAAAACGGGGGGTACTGATCAGCTGCTTTCAACA
CACAGTGGACTGCCAAGCCCGACAACCAAAGTCTCTCCTGTTTCGACCCAACTATTGC
CATCCAAGGGTGTGCCGTCTGATGTCTTCCAGCGAGGTTTACCAGTCTCTGTGGCACC
ATCCAACTTGACTGCAGCTCAAGATTTCCGTGCTCTCTCTCTTCTGTCAACAAGTTCTT
GGGGTTCATGCGACCCAGAACCTCCCGCACTTAACCATTCCATGCATACAAATCATGC
CTCCTTGGCTCCGTCTTTAGCTACCATGCATCCGGTGCCTCTTGGTGGCCCTCCCATTTC
ATCGGAGTTCTGGCAAGCTGATCAACCAGCCTTAGGTGGTCCTGGATCTCACACCTCG
ACTTTCCAGTTGTTCAAAGCACCAAACGAGTCCAGCTTCTATCTTTACTAA

>CqSPL2 AUR62005629-RA 462
MSAISMMELNAKSPFLWDWENLVMFNTKAAETPKKLQSDSEIEGIEGFEVGSFYSSGGRSD
GGTGSDLGYVSSSKSSKSASVDSSADREFKGNQISLEATDCFQQDLRGQRDLVNTSGTTRAS
PTIESSVASGESLIALELGKRTYFEDVSGASNVKNTARSSIPPDSVAKKTKSSSPSIENPRCQVE
GCNLDLSSAKDYHRKHRVCENHSKSPKVVINGLERRFCQQCSRFHSLSEFDQKKRSCRRRL
SDHNARRRKPKPEVIQFNSMRLTSSLYEGRNQLNFAFDQVPMHQTGHGTWDSARESKVTL
VKPGFFTTMKTGGTDQLLSTHSGLPSPTTKVSPVSTQLLPSKGVPSDVFQRGLPVSVAPSNLT
AAQDFRALSLLSTSSWGSCDPEPPALNHSMHTNHASLAPSLATMHPVPLGGPPISSEFWQA
DQPALGGPGSHTSTFQLFKAPNESSFYLY*

>*CqSPL3* AUR62002563-RA 2937bp
ATGGAGGCTGAGATGGGAGGGAAAACTGATGTTTATTGTTCGGCGGTGATGCCGGTGT
CTGACCCGAAAGCAGTAGGAAAGAAGACTTTGGAATGGGATTTGAATGATTGGAAAT
GGGATGGTGATCTTTTTCTTGCCGCTCCATTAAATACTACTCATACACCTTTAGATTGTA
GAAGTAAACAGCTTTTTCCCCTTGGACCAGAACTTGCATCGAATAATAATGTAGGTTCC
GAAGAGAGTAATAAAACTAATGAGAAAGAAAAGAGGGAAATGGAGAAGAGAAGGA
GGGTTGTGGTTGTTCCTCATGAGGAGCTGAATGATGAAGGTAGACCACTGAATTTGAA
GTTGGGTGAGCAGGTTTATCCTATAGCAGAAGACGAAGCTGATAAATTGGAAGGGAA
GAGTGTGAAAAAGAGTAAGTCTGCTGGAACTGGAACTTCCCAACCTGCCTGTCAGGT
AGAAGGTTGTACAGCTGATCTTAGTAATGCCAAGGATTACCATAGACGTCATAAAGTTT
GTGAAGTGCACTCCAAGGTCAGTGAAGCTTTTGTGGGAAATGTTATTCAACGGTTCTG
TCAGCAGTGTAGCAGGTTCCATGCTCTGCCAGAATTTGATGAAGGGAAGAGAAGCTG
TCGTAGACGTCTAGCAGGGCATAATAAGAGGAGAAGAAAAACACTTCCTGAAACTTC
ACCTAATGTTGGTTCCTTGACTGATGAAAAGAGTTCAGGCTACCTACTAGTTAGTCTGC

TGAGAATTTTGTCCAATCTGCACTCAAATGGTTCTGATGAGACAAAAGATCAAGATCT
GATTTCTCATCTTTTGAGAAATCTTGCTAGTCAAGTTAGTGGAAGCAACCTGCCTGAAC
TACAGCAAGGATCTCAGAGTTTACATAATGCAGGAATATCTATCGGTGTTCCAGAAAA
GAACCCTTCTAGCGCACAAGAACTATGTCAAGCTGTGCCTTCAGCAGAAGCACGTATA
GGATTATTGACAAGGGAAAATCAGCATCAGAAAGAAAAAACACAATGTGCTTCACAA
CCGGGCATTTTCCATCCAACTGATGGTTCCATAGCTACAAAAGGAAGTGTACCTGGGG
CTTACCTTGGCATTGACTTGAATAATGTTTATGATGACTCTCAAGAATGTGTAGATATTC
CAGGAAATGGACATGGTTCTATTGCTTGTGCATTCAGGTCAGAACATTTACAGAAATC
AAGTCTACCTCAAACAAGTGGAAACTCCGATTCAAATTCTGGTCATTCTAGTTCTAGTG
GTAGTGATTCACAGGGCCGAACTGATCGGATTGTTTTCAAGCTGTTTGGTAAGGATCC
AAATGACTTGCCTAATCAATTGCGAACACAGATTCTTGATTGGTTGTCCCACAAACCTT
CGGACATTGAAGGGTACATCAGACCTGGCTGCATTGTTTTGACAGTATATCTTCGTTTG
AACAAGTCCTTATGGGAAGAGCTCTGCTATGACATGAGCTCCAGTTTGAGTAGGCTCC
TGAGTCTGTCTGATGATCCATTTTGGAAGACAGGATGGATCTATACCAGAGTGCAGCA
AAGTGCAGCATTTATTTGTGATGGTCGTGTTGTTCTAGATACTCCTCTACCTTTCAAAAG
CCGTGGCAGTAGAATTTCAAGCATCAGTCCCATTGCGGTTCCTACTGCAGAGACAGTG
CAATTAGTTGTTAAGGGTTCTAATCTCTCTGGGCCAACTTCTAGGTTACTTTGTGCTATA
GAAGGGAAGTATTTGGTCCAAGATAGCTGTTATAGCTTGGTAGAGAGTACTGCTGCTG
AGCACAATGAGATTCAGTCTCTTAGCTTTCATTGCTCTATACCAAATGTTGTTGGGAGA
GGATTTATTGAGGTGGAAGATTATGGCCTCAGTGGTTGCTTCTTTCCATTTATAGTTGCA
GAGCCTGAAATTTGTTCTGAGATTTGCATGCTTGAAAGAGTAATGGAGATTGCTGGTAC
AGATGAGGGTATTGAAAGAAGGAACGATGCCTTAGAGTTTATACATGAGATGGGTTGG
CTGCTACATACGAATCGTTTAAGCTCAATGTCAGGCCAAACTAATATCCACTTGGACCT
CTTCCCTTTTGTACGACTAAAGTGGCTAATAGATTATTCAATAAACCATGATTGGTGTGC
TGTTTTAAGAAAACTTTTGGATTTGCTGTTCAGTGGAATTGTAGATACTGGAAATCATG
CTTCTGTTGAAAATGCATTGTCAGAGATTTCTCTCCTCCACACTGCCGTCCAAAGAAAT
AGCAGGTCAATGGTGGAATTTTTGTTAAGATACATACCCAAAAAAGTTAAAAATACAA
TAGGCTTGGAACAGAAGCAGTCACATTATGTACTACCCAGCAGCTTCTTATTTAGACCT
GATGTTACTGGCTCCAATGGGTTGACCCCTTTGCACTTAGCTGCCAGTTGTGCTGGTTT
CGAGAATATGTTAGATGCCTTGCTTGAAGATCCTGGAATGGTTGGTATTGGAGCTTGGG
AAAACGCCCGAGACAGCACAGGATTGACCCCGAAAGATTATGCGTACCTTAGAGGTC
ATAACCATTACATTGATCTATTCCAGAGTAAAGTTAACAAAAATTCATCTGGCAAACAT
GTAGTTGTGGATATCCTTGGACTCTCTAACTTAGAATACAAGCAAAAGCCGTCTGATGA
ACTCAAACTTGCAAAGTTCAATAGCTTATACACTGAGAAGCGGCAAATCAGTCAAAA
CTGCAAACTTTGCGAGCAGAGGCCAAATTATGGATTCAGAGGAACATCACTGACATGT
AGGCCAGTAGTGATGTCTCTTGTTACCATTGCTGTAGTTTGTGTTTGTACAGCTCTGCTT
TTCAAGAGTATGCCTCGAGTTTGTTATGTCTTCGTGCCATTCAGGTGGGACTCGCTGAA
ATATGGAGCAATGTAG

>CqSPL3 AUR62002563-RA 978
MEAEMGGKTDVYCSAVMPVSDPKAVGKKTLEWDLNDWKWDGDLFLAAPLNTTHTPLD
CRSKQLFPLGPELASNNNVGSEESNKTNEKEKREMEKRRRVVVVPHEELNDEGRPLNLKL
GEQVYPIAEDEADKLEGKSVKKSKSAGTGTSQPACQVEGCTADLSNAKDYHRRHKVCEV

HSKVSEAFVGNVIQRFCQQCSRFHALPEFDEGKRSCRRRLAGHNKRRRKTLPETSPNVGSL
TDEKSSGYLLVSLLRILSNLHSNGSDETKDQDLISHLLRNLASQVSGSNLPELQQGSQSLHN
AGISIGVPEKNPSSAQELCQAVPSAEARIGLLTRENQHQKEKTQCASQPGIFHPTDGSIATK
GSVPGAYLGIDLNNVYDDSQECVDIPGNGHGSIACAFRSEHLQKSSLPQTSGNSDSNSGHS
SSSGSDSQGRTDRIVFKLFGKDPNDLPNQLRTQILDWLSHKPSDIEGYIRPGCIVLTVYLRLN
KSLWEELCYDMSSSLSRLLSLSDDPFWKTGWIYTRVQQSAAFICDGRVVLDTPLPFKSRGSRI
SSISPIAVPTAETVQLVVKGSNLSGPTSRLLCAIEGKYLVQDSCYSLVESTAAEHNEIQSLSFH
CSIPNVVGRGFIEVEDYGLSGCFFPFIVAEPEICSEICMLERVMEIAGTDEGIERRNDALEFIHE
MGWLLHTNRLSSMSGQTNIHLDLFPFVRLKWLIDYSINHDWCAVLRKLLDLLFSGIVDTG
NHASVENALSEISLLHTAVQRNSRSMVEFLLRYIPKKVKNTIGLEQKQSHYVLPSSFLFRPDV
TGSNGLTPLHLAASCAGFENMLDALLEDPGMVGIGAWENARDSTGLTPKDYAYLRGHN
HYIDLFQSKVNKNSSGKHVVVDILGLSNLEYKQKPSDELKLAKFNSLYTEKRQISQNCKLCE
QRPNYGFRGTSLTCRPVVMSLVTIAVVCVCTALLFKSMPRVCYVFVPFRWDSLKYGAM

>*CqSPL4* AUR62029983-RA 3570bp
ATGGAGGCTGGGATGGGAGGGATAACCGATGTTTATAGTTCAGCGGTGTTGCCGGTGT
CTGACCCGAAAGCAGTAGGAAAGAAGACTTTGGAATGGGATTTGAATGATTGGAAAT
GGGATGGTGATCTTTTTCTTGCCACTCCATTGAATACTACTCATACACCTTTAGATTGTA
GAAGTAAACAGCTTTTTCCCCTTGGACCGGAACTTGCAATGAACAATAATGTAGGCTC
CGAAGAGATCAGTATAACTAATGAGAAAGGAAAGAGGGAAATGGAGAAGAGAAGG
AGGGTTGTGGTTGTTCCTCATGAGGAGCTGAATGAAGAAGGTAGACCACTGAATTTGA
AGTTGGGTGAGCAGGTTTATCCTATAGCAGAAGATGAAGCTGATAAATTGGAAGGGA
AGAGTGTGAAAAAGAGTAGGTCCGCTGGAACTGGAACTTCCCAACCTGCCTGTCAGG
TAGAAGGTTGTATGGCCAATCTTAGTAATGCCAAGGATTACCATAGACGTCATAAAGTT
TGTGAAGTGCACTCCAAGGTCAGTGAAGCTTTTGTGAGAAATGTTATGCAACGGTTCT
GTCAGCAGTGTAGCAGGTTCCATGCTCTGCCAGAATTTGATGAAGGGAAGAGAAGCT
GTCGTAGACGTCTTGCAGGGCATAACAAGAGGAGAAGAAAAACACTTCCTGAAACTT
CACCTAATGTTGGTTCCTTGACTGATGAAAAGAGTGCAGGCTACCTACTAGTTAGTCTG
CTGAGAATTTTGTCCAATCTGCACTCAAATGGTTCTGATGAGACAAAAGATCAAGATC
TAATTTCTCATCTTTTGAGAAATCTTGCTAGTCAAGTTAGTGGAAGCAACTTGCCTGAA
CTACAGCAAGGATCTCAGAGATTACTCAATGCAGGAATATCTGTCGGAATTCCAGAAA
AGAACCCTTCTAGCGCACAAGAACTATGTCAAGCTGTGCCTTCAGCAGAAGCACATAT
AGGATTACTGACCAGAGAAAATCAGCATCAGAAAGAAAAAACACAATATGCTTCACA
ACCGGGCATTTTCCATCCAGCTGATGGTTCCATAGCTACAAAAGGAAGTGTACCTAGG
GCTTACCTTGGCATTGACTTAAATAATGTTTATGATGACTCTCAAGAATGTGTAGATATT
CCAGGAAATGGACATGGTTCTATTGCTTGTGCATATTGGCCAGAACATTTACAGAAATC
AAGTCCACCTCAAACAAGTGGAATCTCCGATTCAAATTCTGGTCATTCTAGTTCTAGTG
GCAGTGATTCACAGGGCCGAACTGATCGGATTGTTTTCAAGCTTTTTGGTAAGGATCC
AGATGACTTGCCTCATCAATTGCGAACACAGATTCTGGATTGGTTGTCCCACAAACCTT
CGGACATTGAAGGGTACATCAGGCCTGGTTGCATTGTTTTGACAATATATCTTCGTTTG
AACAAGTCTTTATGGGAAGAGCTCTGCTATGACATGAGCTCCAGTTTGAGTAGGCTCC
TGAGTCTGTCTGATGATCCATTTTGGAAGACAGGATGGATCTATACCAGAGTGCAGCA
AAGTGCAGCATTTATTTGTGATGGTCGGGTTGTTCTAGATACTCCTCTACCTTTCAAAA

GCCGTGGCAGTCAAATTTCAAGCATCAGTCCCATTGCGGTTCCTGCTGCTCAGACAGT
GCAGTTAGTTGTTAAGGGTTCTAATCTCTCTGGGCCAACTTCTAGGTTACTTTGTGCTAT
AGAAGGGAGGTATTTGGTCCAAGATAGCTGTTATAGCCTGGTAGAGAGTACTGCCGAG
CAGGATGAGGTTCAGTCTCTTAGCTTTCATTGCTCTATACCAAATGTTGTTGGGAGAGG
ATTTATTGAGGTGGAAGACTATGGCCTCAGTGGTTGCTTCTTTCCATTCATAGTTGCAG
AACCTGAAATTTGTTCTGAGATTTGCATGCTTGAAAGAGTAATGGAGACTGTCGGTAC
AGATGAGGGTATTAATAGAAGGAATGACGCCTTAGAGTTTATACATGAGATGGGTTGG
CTGCTACACGCGGATCGTTTAAGCTCAATGTCTGGCCAACCTAATATCCAGTTGGACCT
CTTCCCTTTTGTACGACTAAAGTGGCTAATAGATTTTTCAATGGACCATGATTGGAGTG
CTGTTTTAAGAAAACTTCTGGATTTGCTGTTCAGTGGAATTGTTGATACTGGAAATCAT
GCTTCTGTCAAAAATGCATTGTCAGAGATGCCTCTCCTCCACACTGCCGTCCAGAGAA
ATAGCAGGTCAATGGTGGAATTTTTGTTAAGATACATACCCAAAAAAGTTAAAAATAC
TAAAGGCTCAGAACAGAAGCAGTCACATTATGAACCACCCATCAGCTTCTTATTTAGA
CCTGATGTTACTGGTTCCAATGGGTTGACCCCTTTGCACTTAGCTGCCAGTTGTGCTGG
TTTTGAGAATATTTTGGATGCCTTGCTTGAAGATCCTGGAATGGTTGGTATTGGAGCTTG
GGAAAACGCCCGAGACAGCACAGGATTGACCCCAAAAGATTATGCTTACCTTAGAGG
TCATAACCATTACATTGTTCTATTCCAGAGTAAAGTTAACAAAATTTCATCTGGCAAAC
ATGTAGTTGTGGATATCCTTGGACTCTCGAACTTAAATTCCAAGCAAAAGCAGTCTGAT
GAACTCAAATCTGCAAAGTTCAATAGCTTATACACTGAGAAGCGGCAAATCAGTCAA
AACTGCAAACTTTGCGTGCAGAGGCCAAATTATGGATTCAGAGGAACATCACTAACAT
GTAGGCCAGTAGTGATGTCTCTTGTTACCATTGCTGTAGTTTGTGTTTGTACAGCTCTGC
TTTTCAAGAAAAAGAGAAGCGATGAGGCTGAGAAGCTTGCTGTAATTGTTGATGTATT
GGTTCAAAATCGTTTTGAGTTGATTGAAGAGATCAATACAAGCTTTGTGCTTGGTGTTG
GCTGGGATTTGATCATGGAAATGCAAAAATTGGAGAAAACGAGTAATGAGCCCAGAA
TCGTTGAAAGTTACCATTTCGTTTCAGTGCTTGCTGAATCAGAATATGGACTGCAAATA
CATGATTTGAGTTCAGAGATCATGTATAGGCTTGATCTCTATGCTTTCCTTTATGAGATG
AAGTGCAAAGCTGTTGACTCATTAAATAAGCCTTCGCCTCCAAAAAAGAACAAATGG
CCTGTCCCACCAGTTAGGCCACAGGGTGACAAATACATATGCATTGATTACATAAATGT
GATGCGTGTAGTTGTTGCAACTAAGGCAAGGGTTTCTTTAATTGAAGTGGCTGAGCTTG
CTGCAGCAAAGTGGGTGGTCAGTACTTGCAAGTCTGCAAAGCAAGATGCTACAAAGC
GAGCTATTCAGTTCTTGCGTGTTTGCTACAATGTAGACATAATTGATCTGAACTATGGC
AAAGGGGTGTATGCTGGTATCAAGTGTGCATTATCTAGGGAGAACAACATATTTCCAA
CTCCTGCATTACCTCCAGCAGCTCCTCGTAAGCCTAAGGCTTCATCTTCGTTTGGTGAA
CATGCTGTTTAG

>CqSPL4 AUR62029983-RA 1189
MEAGMGGITDVYSSAVLPVSDPKAVGKKTLEWDLNDWKWDGDLFLATPLNTTHTPLDCR
SKQLFPLGPELAMNNNVGSEEISITNEKGKREMEKRRRVVVVPHEELNEEGRPLNLKLGEQ
VYPIAEDEADKLEGKSVKKSRSAGTGTSQPACQVEGCMANLSNAKDYHRRHKVCEVHSK
VSEAFVRNVMQRFCQQCSRFHALPEFDEGKRSCRRRLAGHNKRRRKTLPETSPNVGSLTDE
KSAGYLLVSLLRILSNLHSNGSDETKDQDLISHLLRNLASQVSGSNLPELQQGSQRLLNAGIS
VGIPEKNPSSAQELCQAVPSAEAHIGLLTRENQHQKEKTQYASQPGIFHPADGSIATKGSVP
RAYLGIDLNNVYDDSQECVDIPGNGHGSIACAYWPEHLQKSSPPQTSGISDSNSGHSSSSGS
DSQGRTDRIVFKLFGKDPDDLPHQLRTQILDWLSHKPSDIEGYIRPGCIVLTIYLRLNKSLWE

ELCYDMSSSLSRLLSLSDDPFWKTGWIYTRVQQSAAFICDGRVVLDTPLPFKSRGSQISSISPI
AVPAAQTVQLVVKGSNLSGPTSRLLCAIEGRYLVQDSCYSLVESTAEQDEVQSLSFHCSIPNV
VGRGFIEVEDYGLSGCFFPFIVAEPEICSEICMLERVMETVGTDEGINRRNDALEFIHEMGWL
LHADRLSSMSGQPNIQLDLFPFVRLKWLIDFSMDHDWSAVLRKLLDLLFSGIVDTGNHASV
KNALSEMPLLHTAVQRNSRSMVEFLLRYIPKKVKNTKGSEQKQSHYEPPISFLFRPDVTGSN
GLTPLHLAASCAGFENILDALLEDPGMVGIGAWENARDSTGLTPKDYAYLRGHNHYIVLF
QSKVNKISSGKHVVVDILGLSNLNSKQKQSDELKSAKFNSLYTEKRQISQNCKLCVQRPNY
GFRGTSLTCRPVVMSLVTIAVVCVCTALLFKKKRSDEAEKLAVIVDVLVQNRFELIEEINTSFV
LGVGWDLIMEMQKLEKTSNEPRIVESYHFVSVLAESEYGLQIHDLSSEIMYRLDLYAFLYEM
KCKAVDSLNKPSPPKKNKWPVPPVRPQGDKYICIDYINVMRVVVATKARVSLIEVAELAAA
KWVVSTCKSAKQDATKRAIQFLRVCYNVDIIDLNYGKGVYAGIKCALSRENNIFPTPALPPA
APRKPKASSSFGEHAV

>*CqSPL5*   AUR62029984-RA   2943bp
ATGGAGGCTGAGATGGGAGGGAAAGCTGATGTTTATAGTTCAGCGGTGATGCCGGTGT
CTGACCTGAAAGCAGTAGGAAAGAAGACTTTGGAATGGGATTTGAATGATTGGAAAT
GGGATGGTGATCTTTTTCTTGCCACTCCATTGAATACTACTCATACACCTTTAGATTGTA
GAAGTATACAGCTTTTTCCCCTTGGACCAGAACTTGCAACGAACATTAATGTAGGTTCC
GAAGAGATCAGTAAAACTAATGAGAAAGGAAAGAGGGAAATGGAGAAGAGAAGGA
GGTTTGTGGTTGTTCCTCATGAGGAGGTGAATGAAGAAGGTAGACCATCGAATATGAA
GTTGGGTGAGCAGGTTTATCCTATAGCAGAAGATGAAGCTGATAAGTTGGAAGGGAA
GAGTGTGAAGAAGAGTAAGTCTCTTGGAACTGGAACTTCCCAACCTGCCTGTCAGGT
AGAAGGTTGTACGGCTGATCTTAGTAATGCTAAGGATTACCATAGACGTCATAAAGTTT
GTGAAGTGCACTCCAAAGTCAGTGAAGCTTTCGTGGGAAATTTTATGCAACGGTTCTG
TCAGCAGTGTAGCAGGTTCCATAATCTGCCAGAATTTGATGAAGGGAAGAGAAGCTGT
CGTAGACGTCTGGCTGGGCATAACAAGAGGAGAAGAAAAACACTTTCTGAAACTTCA
CCTAATGGTGGTTCCTTGACTGATGAAAAGAGTGCAGGCTACCTACTTGTTAGTCTGCT
GAGAATTTTGTCCAATCTGCACTCAAATGGTTATGACGAGACAAAAGATCAAGATCTA
CTTTCTCATCTTTTGAGAAATCTTGCTAGTCAAGTTAGTGGAAGCAACTTGCCTGAACT
ACAGCAAGGATTTCAGAGGGTACTCAATGCAGGAATATCTGTCGGAATTCCAGAAAA
GAACCCTTCTAGCGCACAAGAACTATGTCAAGCTGTGCCTTCAGCAGAAGCACATATA
GGATTACTGACCAGAGAAAATCAGCATCAGAAAGAAAAAACACAATATGCTTCACAA
CCGGGCATTTTCCATCCAGCTGATGGTTCCATAGCTACAAAAGGAAGTTTACCTGGGG
CTTACCTTGGCATTGACTTGAATAATGTTTATGATGACTCTCAAGAATTTGTAGATATTC
CAGGAAATGGACATGGTTCTATTGCTTGTGCATATTGGCCAGAACATTTACAGAAATCA
AGTCCACCTCAAACAAGTGGAATCTCCGATTCAAATTCTGGTCATTCTAGTTCTAGTGG
CAGTGATTCACAGGGCCGAACTGATCGGATTGTTTTCAAGCTTTTTGGTAAGGATCCA
GATGACTTGCCTCATCAATTGCGAACACAGATTCTTGATTGGTTGTCCCACAAACCTTC
GGACATTGAAGGGTACATCAGGCCTGGTTGCATTGTTTTGACAATATATCTTCGTTTGA
ACAAGTCCTTATGGGAAGAGCTCTGCTATGACATGAGCTCCAGTTTGAGTAGGCTCCT
GAGTCTGTCTGATGATCCATTTTGGAAGACAGGATGGATCTATACCAGAGTGCAGCAA
AGTGCAGCATTTATTTGTGACGCTCTTTTTTCTTGTGCTGAAGGTCGGGTTGTTCTAGAT
ACTCCTCTACCTTTCAAAAGCCGGGACAGTCGAATTTCAAGCATCAGTCCCATTGCGG
TTCCTGCTGCTCAGACAGTGCAGTTAGTTGTTAAGGGTTCTAATCTCTCTGGCCAACT

TCTAGGTTACTTTGTGCTATAGAAGGGAAGTATCTGGTCCAAGATAGCTGCTGTTATAG
CTTGGTAGATAGTACTGCTGCCGAGCAGGATGAGGTTCAGTCTCTTAGCTTTCATTGCT
CTATACCAGATGTTGTTGGGAGAGGATTTATTGAGGTGGAAGATTATGGCCTCAGTGGT
TGCTTCTTTCCATTCATTGTTGCAGAGCCTGAAATTTGTTCTGAGATTTGCATGCTTGAA
AGAGTAATGGAGATTGTCGGTACAGATGAGGGTATTGATAGAAGGAATGTTGCCTTAG
AGTTTATACATGAGATGGGTTGGCTGCTGCATACGAACCGTGTAAGCTCAATGTCAGG
CCAAACTAATATCCAGTTGGACCTCTTCTCTTTTGTACGACTAAAGTGGCTAATAGATTT
TTCAGTGGACCATGATTGGTGTGCTGTTTTAAGAAAACTCCTGGATTTGCTGTTCAGTG
GAATTGTAGATACTGGACATCATGCTTCTGTTGAAAATGCATTGTCAGAGATGCCTCTC
CTTCACACTGCCGTCCAAAGAAATAGCAGGTCAATGGTGGAATTTTTGTTAAGATACA
TACCCAGAAAAGCTAAGAATACAAAAGTCTCAGATGAATCACCCAGCAGCTTCTTATT
TAGACCTGATGTCACTGGTTCCAATGGGTTGACCCCTTTGCACTTAGCTGCCAGTTGTG
CTGGTTTCGAGAATATGTTGGATGCCTTGCTTGAAGATCCTGGAATGGTTGGTATTAGA
GCTTGGGAAAATGCTCGAGACAGCACAGGATTGACCCCAAAAGATTATGCTTACCTTA
GAGGTCATAACCATTACATTGTTCTATTCCAGAGTAAAGTTAACAAAATTTCATCTGGC
AAACATGTAGTTGTGGATATCCTTGGACTCTCGAACTTAAATTCCAAGCAAAAGCAGT
CTGATGAACTCAAATCTGCAAAGTTCAATAGCTTATACACTGAGAAGCGGCAAATCAG
TCAAAACTGCAAACTTTGCGTGCAGAGGCCAAATTATGGATTCAGAGGAACATCACT
AACATGTAGGCCAGTAGTGATGTCTCTAGTTACCATTGCTGTAGTTTGTGTTTGTACAGC
TCTGTTTTTCAAGAGTATGCCTCGAGTTTGTTATGTCTTCTTGCCATTCAGGTGGGACAC
ACTGAAATATGGAGCAGTGTAG

>CqSPL5 AUR62029984-RA 980
MEAEMGGKADVYSSAVMPVSDLKAVGKKTLEWDLNDWKWDGDLFLATPLNTTHTPLDC
RSIQLFPLGPELATNINVGSEEISKTNEKGKREMEKRRRFVVVPHEEVNEEGRPSNMKLGEQ
VYPIAEDEADKLEGKSVKKSKSLGTGTSQPACQVEGCTADLSNAKDYHRRHKVCEVHSKV
SEAFVGNFMQRFCQQCSRFHNLPEFDEGKRSCRRRLAGHNKRRRKTLSETSPNGGSLTDEK
SAGYLLVSLLRILSNLHSNGYDETKDQDLLSHLLRNLASQVSGSNLPELQQGFQRVLNAGIS
VGIPEKNPSSAQELCQAVPSAEAHIGLLTRENQHQKEKTQYASQPGIFHPADGSIATKGSLP
GAYLGIDLNNVYDDSQEFVDIPGNGHGSIACAYWPEHLQKSSPPQTSGISDSNSGHSSSSGS
DSQGRTDRIVFKLFGKDPDDLPHQLRTQILDWLSHKPSDIEGYIRPGCIVLTIYLRLNKSLWE
ELCYDMSSSLSRLLSLSDDPFWKTGWIYTRVQQSAAFICDALFSCAEGRVVLDTPLPFKSRDS
RISSISPIAVPAAQTVQLVVKGSNLSGPTSRLLCAIEGKYLVQDSCCYSLVDSTAAEQDEVQSL
SFHCSIPDVVGRGFIEVEDYGLSGCFFPFIVAEPEICSEICMLERVMEIVGTDEGIDRRNVALEF
IHEMGWLLHTNRVSSMSGQTNIQLDLFSFVRLKWLIDFSVDHDWCAVLRKLLDLLFSGIVD
TGHHASVENALSEMPLLHTAVQRNSRSMVEFLLRYIPRKAKNTKVSDESPSSFLFRPDVTGS
NGLTPLHLAASCAGFENMLDALLEDPGMVGIRAWENARDSTGLTPKDYAYLRGHNHYIV
LFQSKVNKISSGKHVVVDILGLSNLNSKQKQSDELKSAKFNSLYTEKRQISQNCKLCVQRPN
YGFRGTSLTCRPVVMSLVTIAVVCVCTALFFKSMPRVCYVFLPFRWDTLKYGAV

>*CqSPL6* AUR62019452-RA 609bp
ATGATGGTTGTGGTTCAATCGAACCCCTTATTTAAGATTGCGGTGGGAAGAGAAGAGA
GAGGGAAGGTCGTCGGCGAAATTTGGGGTTGTAGAGTCGCCGGCGGGGGGAACGAG
GAACCATCTCTAGTCTTGGGCCGAAGCGACAAGCGGTGGCAGTTAAGGGGAGGGGG

AAAGGGTGGAGATGGAGGAGATGGAGGAGGTAGTGGTGGACTATTGCTGGGGCTGGC
TCGTTGTCAAGCTGAGAAGTGCACGGTGGATCTAACCGAAGCAAAGCGTTACCACCG
CCGTCATAAGGTGTGTGAGCACCATGCTAAGGCCCCTTCTGTGCTTGTTTCCGGCCTCC
GCCAACGCTTCTGCCAGCAATGTAGCAGGTTTCATGAGCTATCTGAGTTCGATGAGAC
AAAGAGAAGTTGCCGGAGGCGTCTAGCAGGGCACAACGAGAGGCGTCGTAAGAGCG
CATCAGAATCTCAGGGAGAAGGCGGTTCGACTTCAAGCCAGAGTAAAGGGTCTAACC
TACAATTAAGGACAGGGGATCAAGCTCAGCTATCCTATTCTGGGAATAACAATAACAA
CTGCACATCCTCTTACAAACAATTTCAGATCAGATAA

>CqSPL6 AUR62019452-RA 202
MMVVVQSNPLFKIAVGREERGKVVGEIWGCRVAGGGNEEPSLVLGRSDKRWQLRGGGKG
GDGGDGGGSGGLLLGLARCQAEKCTVDLTEAKRYHRRHKVCEHHAKAPSVLVSGLRQRF
CQQCSRFHELSEFDETKRSCRRRLAGHNERRRKSASESQGEGGSTSSQSKGSNLQLRTGDQ
AQLSYSGNNNNNCTSSYKQFQIR*

>*CqSPL7* AUR62024322-RA 1050bp
ATGGGTTCCAACTACATGACCGTTGAAGGGTCTTCAACAGTCTCTTCTGGGTTGTCTGA
TTCAATCAACGGACTTAAATTTGGTCAGAAAATCTACTTTGAGGATGGAGGAAAGGCG
GTGTTGCAAAGCGGGCAGCCGCCACGGTGTCAAGTAGAAGGGTGTAATACAGATCTT
AGTGATGCAAAGACTTATTATTCAAGGCATAAAGTTTGTGGAATGCACTCTAAATCAC
CTATTGTTATTGTTGCTGGTATTGAGCAGCGTTTTTGCCAGCAGTGTAGCAGATTTCATC
GGCTTCCTGAATTTGACCAAGGAAAACGAAGCTGTCGCAGACGCCTTGCTGGTCATA
ATGAACGTCGAAGAAAACCACCACCTGGATCTTTGTTATCATCACGTTTGGGGCGTCT
CTCTTCTTCCTTTTTTGGTGACAACACCAGCAAAAATGGTGGGTTTTTATTGGACTTCTC
TTCGTATTCAAGGCAGTCTGAAAAGGATCTGTGGCCAGGTTCAGAAACCTCTGAGCA
GGTATCTGGAAGTCAGTCCAGGTCCATTATGTGGCCAGGCCACTCTGAGGATCATCCTT
CGAAGATGTATCTGCATGGTTCAGCTAGTGAGTCCAGTTATTCCTTCCCTTCTGGAGAA
TGTATCACAGGCGTCTCATCTGACTCTAGCTGTGCTCTCTCTCTTCTGTCAAATCAATCT
TGGGGCTCCAGAAACAGTTCATCAGGTACTGCACTAGGTAACTCGATGAACGTTGATG
GAACTCCCGTCTCCCAGTGTAATGCAGCTCATGGTGTTACAGTTGCACACTTTCCGAA
CAGCAGCTCATCATGGGGTTATAAAGGCAATAATGATGCCAGTTGTGGTTCAGATGGT
GTGCCATCTGGTCATTTGGGACTTGGCCAGATCTCCCAACCACATTATAATGGTCAATT
TCATGGTGTTCTTCTGATGGACCATGTCCAAGATGGCGGACGGCAATACATGGATGTC
GACCATTCTAGGGCTTACAACTCAAACAACACAAACCATGTGGACTGGTCACTTTGA

>CqSPL7 AUR62024322-RA 349
MGSNYMTVEGSSTVSSGLSDSINGLKFGQKIYFEDGGKAVLQSGQPPRCQVEGCNTDLSDA
KTYYSRHKVCGMHSKSPIVIVAGIEQRFCQQCSRFHRLPEFDQGKRSCRRRLAGHNERRRK
PPPGSLLSSRLGRLSSSFFGDNTSKNGGFLLDFSSYSRQSEKDLWPGSETSEQVSGSQSRSIMW
PGHSEDHPSKMYLHGSASESSYSFPSGECITGVSSDSSCALSLLSNQSWGSRNSSSGTALGNS
MNVDGTPVSQCNAAHGVTVAHFPNSSSSWGYKGNNDASCGSDGVPSGHLGLGQISQPH
YNGQFHGVLLMDHVQDGGRQYMDVDHSRAYNSNNTNHVDWSL*

>*CqSPL8A* AUR62004146-RA 849bp

ATGTTGCGCTACGAATGGAACAATCATTCCCCCCCTCCCCCTAACCTAATCCTCTCCG
ACATCGACCTCCACCACCAGCATCACAATCATAATAACAACAATCATGACTTCACCA
CCCCATCAAACCATCACAACCACCACCACCGTCCAATCTTTGACGACTCGTTCTCGAT
ACCTTACGACAACACTCCTCCACAAATCCCACTGTTCTCCGACCCACGTGGGTTCCCA
ACAGCCGGTTCATCCTCCTTACTAACCCTCGAGTCACTCTCCTCCGTCATGCAAATCC
CCAAAACCGAACCAGATCTTTTTACGAACCGCCCCATTGGTTTGAACTTAGGTCGGCG
CACTTACTTCTCCGCCGATGATGACCTCGTCAGCCGGCTCTACCGCCGGTCTAGGGCG
GTCGAACCGGCGTTGCACTCACCTAAATGTCAAGCTGAAGGTTGTAATGCTGACCTTA
GCCATGCTAAGCATTACCACCGTCGTCATAAGGTTTGCGAGTTTCACTCTAAAGCTTC
TACCGTAATCGCTGCCGGGTTAACCCAGCGTTTCTGCCAGCAGTGCAGCCGGTTTCAT
CTGCTATCGGAATTTGATAACGGGAAGAGAAGTTGCCGGAAAAGATTGGCTGATCAC
AATCGCCGACGTCGAAAATCGAACAATCAACGGATTACTTCTACTGCACCATCTATG
TCTTCAGGGGAAAATGCTCAATCTTCTCCCTCCCAAACTATTCTAAGGTCTCCATCGG
ATTCGGGGATGAACTCATCATCATCGGTGACAATTGCGGTATCACCACCAAGGGTAT
CATTTGATTCAAGTTTGATGCAAATGAGGTCATTTGAGTAG

>CqSPL8A AUR62004146-RA 282
MLRYEWNNHSPPPPNLILSDIDLHHQHHNHNNNNHDFTTPSNHHNHHHRPIFDDSFSIP
YDNTPPQIPLFSDPRGFPTAGSSSLLTLESLSSVMQIPKTEPDLFTNRPIGLNLGRRTYFSADD
DLVSRLYRRSRAVEPALHSPKCQAEGCNADLSHAKHYHRRHKVCEFHSKASTVIAAGLTQ
RFCQQCSRFHLLSEFDNGKRSCRKRLADHNRRRRKSNNQRITSTAPSMSSGENAQSSPSQTI
LRSPSDSGMNSSSSVTIAVSPPRVSFDSSLMQMRSFE*

>*CqSPL8B* AUR62013707-RA 861bp
ATGTTACGCTACGAATGGAACAATCATTCCCCCCCTCCCCCTAATCTAATCCTCTCCG
ACATCGATCTCCACCACCACCATCATCATCATCATAATCATAACAACAATCATGAATT
CACCACCCCATCACACCATCACAACCACCACCACCACCACCGTCCAATCTTCGACGA
CTCGTTCTCAATACCCTACGAAAACACTCCCCCACAAATCCCACTCTTCTCCGACCCA
CGTGGGTTCCCAACAGCCGGTTCATCCTCCTTACTAACCCTTGAGTCACTGTCCTCCGT
CATGCAAATCCCCAAAACCGAACCGGATCTTTTTACGAACCGTCCCATTGGTTTGAAC
TTAGGCCGGCGTACTTACTTCTCCGCCGATGATGACCTCGTCAGCCGGCTCTACCGCC
GGTCTAGGGCGGTCGAACCGGCGTTGCACTCACCTAAATGTCAAGCTGAAGGTTGTA
ATGCTGACCTTAGCCATGCTAAGCATTACCACCGTCGTCATAAGGTTTGCGAGTTTCA
CTCTAAAGCTTCTACAGTAATCGCCGCCGGGTTAACTCAGCGTTTCTGCCAGCAGTGC
AGCCGGTTTCATCTGCTATCGGAATTTGATAATGGGAAGAGAAGTTGCCGGAAAAGA
TTGGCTGATCACAATCGCCGACGTCGAAAATCGAATAATCAACGGATTACTTCTACTG
CACCGTCTATGTCTTCAGGGGAAAATGCTCAATCTTCTCCCTCCCAAACTATTTTAAG
GTCTCCATCGGATTCGGGGATGAACTCATCATCATCAGTGACAATTGCAGTATCACCG
CCAAGGGTGTCATTTGATTCAAGTTTCATGCAAGTAAGGTCATTTGAGTAG

>CqSPL8B AUR62013707-RA 286
MLRYEWNNHSPPPPNLILSDIDLHHHHHHHHNHNNNHEFTTPSHHHNHHHHHRPIFD
DSFSIPYENTPPQIPLFSDPRGFPTAGSSSLLTLESLSSVMQIPKTEPDLFTNRPIGLNLGRRTYF
SADDDLVSRLYRRSRAVEPALHSPKCQAEGCNADLSHAKHYHRRHKVCEFHSKASTVIAA

GLTQRFCQQCSRFHLLSEFDNGKRSCRKRLADHNRRRRKSNNQRITSTAPSMSSGENAQSS
PSQTILRSPSDSGMNSSSSVTIAVSPPRVSFDSSFMQVRSFE*

>*CqSPL9* AUR62012061-RA 1122bp
ATGGGTTCCAACTACATGAACGTTGAAGGGTCTTCAACTTCCTCTTCTGGGTTGTCTGA
TTCAATCAACGGTCTTAAATTTGGTCAGAAAATCTACTTTGAGGATGTGGGTAGCGGC
GGCGGCGGTTCCGGCAAGTCTTCCGGCGCCGGCGGTGGCGGTTGTGGGCCGGTGAAA
AAGGGAGGGAAGGCGGTATTGCAAAGCGGGCAGCCGCCGCGGTGTCAAGTGGAAGG
GTGTAATACAGATCTTAGTGATGCAAAAACATATTATTCAAGGCATAAAGTTTGTGGCA
TGCACTCTAAATCACCTATTGTCATTGTTGCTGGTATTGAGCAGCGTTTTTGCCAGCAGT
GTAGCAGATTTCATCGACTTCCTGAATTTGACCAAGGGAAACGAAGCTGTCGCAGAC
GCCTTGCTGGTCATAATGAACGTCGAAGAAAACCACCACCTGGATCTTTGTTATCATC
ACGTTTGGGACGTCTCTCTTCTTCCTTTTTTGGTGACAACACCAGCAAAAATGGTGGGT
TTTTATTGGACTTCTCTTCGTATTCAAGGCAGTCTGAAAAGGATCTGTGGCCAGGTTCA
GAAACCTCTGAGCAGGTATCTGGAAGTCAGTCCAGGTCCATTGTGTGGCCAGGCCACT
CTGAGGATCATCCTTCGAAGATGTATCTGCATGGTTCAGCTAGTGAGTCCAGTTATTCC
TTCCCTTCTGGAGAATGTATCACAGGCGTCTCATCTGACTCTAGCTGTGCTCTCTCTCTT
CTGTCAAATCAATCTTGGGGCTCCAGAAACAGTTCATCAGGTACTGCACTTGGTAACT
CGATGAACGTTGATGGAACTCCCGTCTCCCAGTGTAATGCAGCTCATGGTGTTACAGTT
GCACACTTTCCAAACAGCAGCTCATCATGGGGTTTTAAAGGCAATAATGATGCTAGTT
GTGGTTTACATGGTGTGCCATCTGGTCATTTGGGACTTGGCCAAATCTCCCAACCACAT
TATAATGGTCAATTTCATGGTGATCTTCTGATGGACCATGTCCAAGCTGGCGGACAGCA
ATACATGGATGTTGACCATTCTAGGGCTTACAACTCAAACAACACAAACCATGTGGAC
TGGTCACTTTGA

>CqSPL9 AUR62012061-RA 373
MGSNYMNVEGSSTSSSGLSDSINGLKFGQKIYFEDVGSGGGGSGKSSGAGGGGCGPVKKG
GKAVLQSGQPPRCQVEGCNTDLSDAKTYYSRHKVCGMHSKSPIVIVAGIEQRFCQQCSRFH
RLPEFDQGKRSCRRRLAGHNERRRKPPPGSLLSSRLGRLSSSFFGDNTSKNGGFLLDFSSYSR
QSEKDLWPGSETSEQVSGSQSRSIVWPGHSEDHPSKMYLHGSASESSYSFPSGECITGVSSDSS
CALSLLSNQSWGSRNSSSGTALGNSMNVDGTPVSQCNAAHGVTVAHFPNSSSSWGFKGN
NDASCGLHGVPSGHLGLGQISQPHYNGQFHGDLLMDHVQAGGQQYMDVDHSRAYNSN
NTNHVDWSL*

>*CqSPL10* AUR62011728-RA 2955bp
ATGATGGAACAAGCTCACCGGCTCTCTGAATTGAGAAAGAGGGGTATTGAATGGGATT
TGAATGACTGGAAATGGGATGGTGACCTATTTATAGCCACCCCTTCCAACTCTCAAGG
TCAGCAATTCATTCCACTTGTTCCTGTTCCCGGCAATTCCTCTAACACTTCGTCGTCGTG
CTCTGATGATGTTGATGATGGAACTGGAAGGAGGGATTTGGAGAGGAAGAGGAGGGT
TTTTGTTGTTGACCAAGACTCTTTGGAGGAAGCTGCTCCCTTGACTTTAAAGCTTGGTG
TTCCTCACAGGGAAAGGGACCATTGGGAGACCTCCACTGCCAAGAAGACTAAGTTGC
CCTCGACTTCTTCCACTCGCGCCGTTTGCCAGGTTGAGGACTGTGAAGCTGATCTCAC
CAAGGCTAAGGACTATCACAGGCGTCACAAGGTCTGTGAGCTGCATTCTAAAGCCAC
CAAAGCTCTTGTTGCCAATGTCATGCAGCGTTTTTGCCAGCAGTGTAGTAGGTTTCATG

CTCTACAAGAATTCGATGAAGGCAAGCGAAGCTGTAGACGTCGCTTGGCTGGTCATAA
TAAGCGGCGCCGGAAAACGCAACCTGAAACAGCTGTTCAGGGAAATTCCATGGATGA
CCAAACTAATAGTGTTCTGTTGATGAGCTTACTCAGGATACTCTCCAACATGCATGGTA
ATAACAGAGCAAACCAGACTACAGATCAAGATCTTGTTGCCCAACTCTTGAAAAGCC
TTGCCAACCCTTCTGGGTTGCACTCAGGGAAAGGGTTATCTGGGCTCCTGCATGAATC
ACAAAAGCTGCTAAATGGTGGTATGGCCACTGGTAATGGGCATTCAGAAAAGATGTC
AGCTTATCTCTCAAATGATCAACAAAATACCCCTAGAGTCATTGACCAACATGTTCAA
CTACCCGATTCAGAGATTCCAAGAAAGGGATTGTATCCCGCTAATAGTAGGGGCAGTG
AAATTCAGGCTGTATCCTTGGAAGAACCTAAAAGTTTGTTCCCTATAAAAGATAGCCCT
CCAGCTTACTCTGAGACCACAGAAGGGAGGATGAAGCTGAACAATTTTGACCTGAAT
GATGCATATGTAGATTCAGATGATGGAATGGAAGATTTGGAAAGGTCGCCTGTTAATG
AGAATTTTGCAACTGGCTCTGTTGATTTCCCTTCATGGGCACGGCAGGATTCACACCA
ATCAAGTCCACCACAGACTAGTGGCAATTCTGACTCAGCCTCTGCTCAATCACCTTCT
AGCTCTAGTGGAGAAGCTCAGAGTCGTACTGATCGGATCGTTTTCAAGCTATTTGGTAA
AGAGCCAAATGACTTTCCGATTATGCTGCGGGGACAGATCCTTGACTGGCTTGCTCAT
AGTCCTACTGAGATTGAGAGCTACATCAGACCTGGCTGTATAATTCTAACAATTTATCT
TCGCTTGGCTGAGTCCTCGTGGGAGGAGCTCTGCTCTGACCTTTCATCGCGCTTAACTC
AACTCTTTGACATATCTGATGACACATTTTGGAGGATGGGATGGGTGTATGTACGAGTG
CAGAATCAAATAGCAATTGTTCATAATGGTGAAGTTGTCTTGGATACGTCGTTATCTCT
AAAAGATAATAATTGCTGTAGAATTTTGAGTGTCATGCCTATAGCCGTGTCTATGAATG
AACAGGTTCAATTTAAAGTTAGAGGTTTCAACCTTTCTCAGTCCACAACAAGATTACT
CTGTGCTCTAGAAGGGAAATATCTTGATCAAGAAGTATCTCAAGAATCAGAGGTTGGC
GATTTCCTTGAGGATGATGATGAGACTGACCATGCCAATTTGTCTTGCATCATTCCAAA
AGCAACTGGAAGAGGGTTTATTGAGGTTGAAGATCATGGTCTCAGCAGCAGTTTCTTC
CCTTTTATAGTTGCAGAAAAGGACGTTTGCTCTGAGATTCGGACTCTGGAGAATGTCCT
GGAGCTGAAGAAGACCGATGAAGAAGCATATGAAATTAATAAAACCCTAGAATCCTG
GTGTCAAGCTATGGATTTCATTAATGAAATGGGTTGGCTGCTTCATAGAAGCCACTTGA
AATCTAGATTAGCTGACCTAGATCCTAACACTGTCATATTTTCTTTTAGAAGGTTTAAAT
GGCTCATGGACTTCTCCATGGACCATGATTGGTGTGCTGTTGTTAAGAAACTTTTGGAC
ATTTTGTTTGCTGGAACTGTGGGGTTGGGGGAACATTCTTCTCTGAAGGTTGCATTGTC
AGAAATGGGTATCCTTCACAGAGCTGTACGTAGAAATTCTAGGCCTATGGTGGAGTTT
CTTTTGAGATATGCCCCTCTGAATGTATCGGAAGAATTCATATCATCCAATGATGGTGG
CCAAGTAAGGTTTTTGTTTCGGCCTGATGCCCAGGGACCTGCTGGTTTGACACCACTC
CATGTTGCAGCTGGCAGAGATGGCTCTGAAGATATATTGGACGCTCTAACTGATGACC
CTGGAAAGAATGGAATTGATGCATGGAAGAATGCTCGCGATAGCACAGGGGCTACCC
CTGAGGATTATGCTCGTCTGCGGGGCCACTATGCTTACATACACATTGTCCAGAGGAA
GATTTACCGGAGTTCAACTTCAGGGCATGTGGTGGTTGACATTCCAGGAGAACAATCT
GTTGCCCCAAGGCAAGATGGGGCTCTGAGCTTTGAGGTTGGAAGAAGTGCGTCGCTT
GCTATGAATCAGAGCTGCAAGCTATGTGATCAGAAGAAGATGTCTGTATATTATGGAA
GTAGATCAAGGGCATCTCTAGTGTACAGGCCAGCAATGCTGTCCATGGTTGGTATTGCT
GCAGTGTGTGTGTGTGTGGCTCTTCTTTTCAAGAGCATGCCTAATGTTGTCTGTCTCTTC
CAGCCCTTCCGTTGGGAAATGCTCAATTATGGTTCCAGCTGA

>CqSPL10 AUR62011728-RA 984

MMEQAHRLSELRKRGIEWDLNDWKWDGDLFIATPSNSQGQQFIPLVPVPGNSSNTSSSCSD
DVDDGTGRRDLERKRRVFVVDQDSLEEAAPLTLKLGVPHRERDHWETSTAKKTKLPSTSST
RAVCQVEDCEADLTKAKDYHRRHKVCELHSKATKALVANVMQRFCQQCSRFHALQEFDE
GKRSCRRRLAGHNKRRRKTQPETAVQGNSMDDQTNSVLLMSLLRILSNMHGNNRANQTT
DQDLVAQLLKSLANPSGLHSGKGLSGLLHESQKLLNGGMATGNGHSEKMSAYLSNDQQN
TPRVIDQHVQLPDSEIPRKGLYPANSRGSEIQAVSLEEPKSLFPIKDSPPAYSETTEGRMKLNN
FDLNDAYVDSDDGMEDLERSPVNENFATGSVDFPSWARQDSHQSSPPQTSGNSDSASAQSP
SSSSGEAQSRTDRIVFKLFGKEPNDFPIMLRGQILDWLAHSPTEIESYIRPGCIILTIYLRLAESS
WEELCSDLSSRLTQLFDISDDTFWRMGWVYVRVQNQIAIVHNGEVVLDTSLSLKDNNCCRI
LSVMPIAVSMNEQVQFKVRGFNLSQSTTRLLCALEGKYLDQEVSQESEVGDFLEDDDETDH
ANLSCIIPKATGRGFIEVEDHGLSSSFFPFIVAEKDMDFSMDHDWCAVVKKLLDILFAGTVG
LGEHSSLKVALSEMGILHRAVRRNSRPMVEFLLRYAPLNVSEEFISSNDGGQVRFLFRPDAQ
GPAGLTPLHVAAGRDGSEDILDALTDDPGKNGIDAWKNARDSTGATPEDYARLRGHYAYI
HIVQRKIYRSSTSGHVVVDIPGEQSVAPRQDGALSFEVGRSASLAMNQSCKLCDQKKMSVY
YGSRSRASLVYRPAMLSMVGIAAVCVCVALLFKSMPNVVCLFQPFRWEMLNYGSS*

>*CqSPL11* AUR62029416-RA 1044bp
ATGGAGTGGGACTCTAAATCATGTGCTTGGAATGGTGTGAGTAAGGTGGAGTTTCAAG
ATAATGGTTACCATCATCATCTGGCTACGTTAGCAGGTTCGAGTGGCACCGGAATTAAT
AACATGTTTTCAGTGGATCTGAAGCTGGGAAGATTGGGAGATATGGGAGATGTATCTAT
GGATACCTTAAAGAATTCAATGCCTTTAAATATGGCTTCTTCGTCTTCTCCTTCACCGGT
TTTGGCGCTTTCAAAGAGGGGAAGGCCAGGCAACAGCGGCGCCCAAAGTACTGTTAT
TTGCTCGGTTGATGGGTGTGCTTCTGACCTTAATCAATGCAGGGAATATCATCGCCGCC
ATAAGGTATGTGAGAGGCACTCTAAGACGCCTGTTGTTCTTGTTGGTGGGAAGGAGCA
ACGGTTTTGCCAGCAGTGTAGCAGGTTTCATTCCCTGGAAAAGTTTGATGAGGTAAAG
AGAAGCTGCAGAAAACGCTTGGATGGACATAATCGCCGTAGGAGGAAAAGACAGCC
TGAAACTTTTAACATGCCTGATTCTGCTGGTGGCGTTTTGTCATCTCAAACAGCAGATG
GAATGCTGCAGTATTCCTGCCCCCAAATGCATACTGTAAATTGGCCTACAATGTCTCAG
CCTGATTTGTACTTCCAAAATAATCAAGAAACAATATATCAGCCACTTCTGAATGACGT
TGCTCCAACCGGAAGTAGGAGAGGCGGCCTAAGGGTTTCACCTGATAACTCAGGATG
TGCTCTCTCTCTTCTGTCAAGGTATTCATCCCAATCGTCAGATATCCGTGTGGGACCTGT
TATGCAACCAACGGTTATGAGTTCAGCAACAGCTCAAGGTAGCAGTACTTCTTTGCAT
CTAAATAACAGCTATCAACTTCCTTGCTCTCAGGGTTTGGACGATATTGAGAGTTCAGC
TTCACTCTCTAGCAGTTCTAATACTAATGCTCCAAACATTGGAGGTTTCCACACAGGCC
ATGCAGGGTTTCGCGAAAACACTAGGATTTTCCCATTTGGTTGGGAGTAG

>CqSPL11 AUR62029416-RA 347
MEWDSKSCAWNGVSKVEFQDNGYHHHLATLAGSSGTGINNMFSVDLKLGRLGDMGDVS
MDTLKNSMPLNMASSSSPSPVLALSKRGRPGNSGAQSTVICSVDGCASDLNQCREYHRRH
KVCERHSKTPVVLVGGKEQRFCQQCSRFHSLEKFDEVKRSCRKRLDGHNRRRRKRQPETF
NMPDSAGGVLSSQTADGMLQYSCPQMHTVNWPTMSQPDLYFQNNQETIYQPLLNDVAPT
GSRRGGLRVSPDNSGCALSLLSRYSSQSSDIRVGPVMQPTVMSSATAQGSSTSLHLNNSYQL
PCSQGLDDIESSASLSSSSNTNAPNIGGFHTGHAGFRENTRIFPFGWE*

>*CqSPL12* AUR62039662-RA 1638bp
ATGGAATCCTGGTGGTTTGATTCATTGGATAAGGGTTTTGAATCAAATGAAGCAATCTC
TCAATCTGATTCAATTATTAAAGGTAAAAATGTGTTGATAGGTTGGGAACATAAATCCC
CTCTTAGCAATGAGGATAGTGTGTTAACCCCTAGTCAACGATCAGTCGAAAATCGTAG
TTTTTCTGAATTGGGTATTGCAGAAGTGGTAAGAAGACAATGTCCTACTGATTCAACAA
GGAATGAGTTAGAGGATAATGGTAGTGATGGAGATATTTATAGTTCTTATGTTACTACTA
ATGCCATTTCTGGAGATGATGAATCAAGTTCTAAATTTTCAAGCTCGGTTATGGACTCA
AGTAGCCGTGAATCACCACTCATTGATTTGAAATTGGGTGGATTTGGTGATAATCCAAA
TTCTAGTTCAACTAGAACAGCAACAGCTCATGTTTTGTCTTCAGCTGACTCTTCAACAC
CTCCAAAAAGGGTTCGAGTTATCTCTCAGGCTGTACATTGCCAAGTTTATGGCTGCCAT
AAAGATCTCAGTTCCGCGAAAGATTACCACAAGAGGCATAAAGTTTGTGATGTTCATT
CGAAGACTCCTAAAGTTATTGTTAATGGTATTGAGCAGAGGTTTTGTCAGCAGTGTAGC
AGGTTCCATTTGCTGGGTGAGTTTGATGATGGTAAGCGAAGCTGTCGTAAACGACTTG
CAGGTCACAATGAAAGGAGAAGGAAACCTCAAGTAGGCTTTAGTAATAGAAATGGGA
GATCGTTTCAGTCATACACTGGAAGCAATTTTCAGGGGTTTACACCTACCTCAACATCC
TTTATTTGCCAAGATATACTACCAAGAGGCATTTCCCATACAGTGAAATATGGAACGAA
TGATTGGGTTAAGCATATCAAGGTTGAGGATGGAACAGGCTGTACACAAACGCCAAC
ATATAGTTGCATAAATCGACAGCTTCAGCCAAAATCTATTTTGCCCCCTTATGATTTTGA
AAAACAGTTCCCTTTTATTGACAACACAAATAATACTGGAACACAATTTCCCTTTGGC
AAGAATGTCAATCAACATACGCCTGAAATAGTTTCACATTCCTTGTTTCAAACAAACT
CACCTAGGAATGAAGACTTGGCTCTTTTGGACGCAGCATCAACCGTTCAAGAATTAGC
AGGAATATCAGAGTCCGGTTGTGCTCTCTCTCTTCTGTCATTTCAATCACAAAATTCTTC
TGGCCATTCATCTGCAATGCCTGGATGTCACCCTGTAGTTATACCAAGCAGTAGTCCAC
AATATAGTGTGAACGAAGTCTCTGAAAAAATCTTTGGAAATGGTGCTCAGGCTTTAAC
AAGTGAAGTTCAAAACAGGTATTCTTCGGTTATTGTCAGCTCTACAGAAAGAAATCAA
CCAAGTTTCATGTCAATGCTTAATTATGGTAATAATGCACACTCTGAATTTGGAAATGA
GATACACCACCGTTCCAAATTTATGAACATCAAGGATCATTTGTTGTGTGAAGATGGG
ACAACTATTGATTTGCTTCAATTGTCATCTCAGCTGCAGCGAGTGGAAAACCAGAAGC
TGTCTGAACCTCTTAAGCAGGACACTGAAAATTCTTTCTGCCTTGGAATGATATGA

>CqSPL12 AUR62039662-RA 545
MESWWFDSLDKGFESNEAISQSDSIIKGKNVLIGWEHKSPLSNEDSVLTPSQRSVENRSFSEL
GIAEVVRRQCPTDSTRNELEDNGSDGDIYSSYVTTNAISGDDESSSKFSSSVMDSSSRESPLID
LKLGGFGDNPNSSSTRTATAHVLSSADSSTPPKRVRVISQAVHCQVYGCHKDLSSAKDYHK
RHKVCDVHSKTPKVIVNGIEQRFCQQCSRFHLLGEFDDGKRSCRKRLAGHNERRRKPQVG
FSNRNGRSFQSYTGSNFQGFTPTSTSFICQDILPRGISHTVKYGTNDWVKHIKVEDGTGCTQ
TPTYSCINRQLQPKSILPPYDFEKQFPFIDNTNNTGTQFPFGKNVNQHTPEIVSHSLFQTNSP
RNEDLALLDAASTVQELAGISESGCALSLLSFQSQNSSGHSSAMPGCHPVVIPSSSPQYSVNE
VSEKIFGNGAQALTSEVQNRYSSVIVSSTERNQPSFMSMLNYGNNAHSEFGNEIHHRSKFM
NIKDHLLCEDGTTIDLLQLSSQLQRVENQKLSEPLKQDTENSFCLGMI*

>*CqSPL13* AUR62032118-RA 1689bp
ATGATTTTGATTGTGTTTTCTGGTGCTAAACATCAAATTAAGAATTTAATTGCAAATTTT
TGTTTGGGGATTTCATATGTGGTGTTTGGGATATTGATCTTGTTATATATTATGGAATCCT

GGTGGTTTGATTCATTGGATAAGGGTTTTGAATCAAATGAAGCCATCTCTCAATCTGAT
GCAATTGTTAGAGGTAAAAATGTGTTAATAGGATGGGAACATAAAATCACTAATGAGG
ATAGTGTGCTAACCCCTAGTCAACAATCCGTCGAAAATCGTAGTTATTCTGAATTGGGT
ATTGCAGAAATGGTAAGAATACAATGTCCTACTGATTCAACAAGGAATGAGTTAGAGG
ATAATGGTAGTGTTGGAGATTTTTATAGCTCTTATGTTACTACTAATGCCTTTTCTGGAGA
TGATGAATCAAGTTCTAAATTTTCGAGCTCGGTTATGGACTCAAGTAGCCGTGAATCAT
CACTAATTGATTTGAAATTGGGTGGATTTGGTGATAATCCAAATTCTAGTTCGACTAGA
ACAGCAACAGCTCATGTTTTGTCTTCAGCTGACTCTCCAACACCTCCAAAAAGGGCTC
GAGTGAGCTCTCAGGCTGTACATTGCCAAGTTTATGGCTGCCATAAAGATCTCAGTTCC
GCGAAAGATTACCACAAGAGGCATAAAGTTTGTGATGTTCATTCAAAGACTCCTAAAG
TTATTGTTAATGGTATTGAGCAGAGGTTTTGTCAGCAGTGTAGCAGGTTCCATTTGCTG
GGTGAGTTTGATGATGGTAAGCGAAGCTGTCGTAAACGACTTGCAGGTCACAATGAA
AGGAGAAGGAAACCTCAAGTAGGCTTTAGTAATAGAAATGGGAGATCGTTTCAGTCA
TACACTGGAAGCAATTTTCAGGGGTTTACACCTACCTCAACATCCTTTATTTGCCAAGA
TATACTACCAAGAGGCATTTCCCATACAGTGAAATATGGAACGAATGATTGGGTTAAG
CATATCAAGGTTGAGGATGGAACAGGCTGTACACAGATGCCAACATATAGTTGCATAA
ATAGACAGCTTCAGCCAAAATCTATTTTGCCCCCTCAAGATTTTGAAAAACAGTTCCCT
TTTATTGACAACACGAATAATACTGGAACACAATTTCCCTTTGGCAAGAATGTCAATCA
ACATACGCCTGTAATAGTCTCACATTCCTTGTTTCAAACAAACTCACCTAGGAATGAA
GACTTGGCTCTTTTGGACGCAACATCAACCGTTCAAGAATTAGCAGGAATATCAGAGT
CCGGTTGTGCTCTCTCTCTTCTGTCATTTCAATCACAAAATTCTTCTGGCCATTCATCTG
CAATGCCTGGATGTCACCCTGTAATTATACCAAGCAGTAGTCCACAATATAGTGTGAAC
GAAGTCTCTGAAAAAATCTTTGGAAATGGTGCGCAGGCTTTAAAAAGTGAAGTTCAA
AACAGGTATTCTTCGGTTATTGTCAGCTCTACAGGAAGAAATCAACCAAGTTTCATGTC
AGTGCTTAATTATGGTAATAATGCACACTCTGAATTTGGAAATGAGATACACCACCGTT
CCAAATTTATGAACATTAAGGATCATTTGTTGTGTGAAGATGGGACTACTATTGATTTGC
TTCAATTGTCATCTCAGCTGCATGGAAAACCAGAAGCTGTCTGA

>CqSPL13 AUR62032118-RA 562
MILIVFSGAKHQIKNLIANFCLGISYVVFGILILLYIMESWWFDSLDKGFESNEAISQSDAIVR
GKNVLIGWEHKITNEDSVLTPSQQSVENRSYSELGIAEMVRIQCPTDSTRNELEDNGSVGDF
YSSYVTTNAFSGDDESSSKFSSSVMDSSSRESSLIDLKLGGFGDNPNSSSTRTATAHVLSSADS
PTPPKRARVSSQAVHCQVYGCHKDLSSAKDYHKRHKVCDVHSKTPKVIVNGIEQRFCQQC
SRFHLLGEFDDGKRSCRKRLAGHNERRRKPQVGFSNRNGRSFQSYTGSNFQGFTPTSTSFIC
QDILPRGISHTVKYGTNDWVKHIKVEDGTGCTQMPTYSCINRQLQPKSILPPQDFEKQFPFI
DNTNNTGTQFPFGKNVNQHTPVIVSHSLFQTNSPRNEDLALLDATSTVQELAGISESGCAL
SLLSFQSQNSSGHSSAMPGCHPVIIPSSSPQYSVNEVSEKIFGNGAQALKSEVQNRYSSVIVSS
TGRNQPSFMSVLNYGNNAHSEFGNEIHHRSKFMNIKDHLLCEDGTTIDLLQLSSQLHGKPE
AV*

>*CqSPL14* AUR62003425-RA 1050bp
ATGGAGTGGGACTCTAAATCATGTGCTTGGAATGGTGTGAGTAAGGTGGAGTTTCAAG
ATAATGGTTACCATCACCATCTGGCTACGTTAGCAGGTTCGAGTGGCACCGGAATTAGT
AACATGTTTTCAGTGGATCTGAAGCTGGGAAGATTGGGAGATATGAGAGATCTATCTAT

GGATACCTTAAAGAATTCAATGCCTTTGAATATGGCTTCTTCGTCGTCTCCTTCACCGGT
TTTGGCGCTTTCAAAGAGGGGAAGGCCACGCAACAGCGGCGCCCAAAGTACTGTTAT
TTGCTCGGTTGATGGGTGTGCTTCTGACCTTAATCAATGCAGGGAATATCATCGCCGCC
ATAAGGTATGTGAGAGGCACTCCAAGACGCCTGTTGTTCTTGTTGGTGGGAAGGAGCA
ACGGTTTTGCCAGCAGTGTAGCAGGTTCCATTCCCTGGAAGAGTTTGATGAGGTAAAG
AGAAGCTGCAGAAAACGCTTGGATGGACATAATCGTCGTAGGAGGAAAAGACAGCC
TGAAACTTTTTACATGCCTGATTCTGCTGGTGGCGTTTTGTCATCTCATACAGCAGATGG
AATGCTGCAGTATTCCTGCCCCCAAATGCATACTGTAAATTGGCCTACAATGTCTCAGC
AGCCTGATTTGTACTTCCAAAATAACCAAGAAACAATATATCAGCCACTTCTGAATGA
CGTTGCTCCAACCGGAAGTAGGAGAGGCGGCCTAAAGGTTTCACCTGATAACTCAGG
ATGTGCTCTCTCTCTTCTGTCAAGGTATTCATCCCAATCGTCAGATATCCGTGTGGGACC
TGCTATGCAACCAACGGTTATGAGTTCAGCAACAGCTCAAGGTAGCAGCACTTCTCTG
CATCTAAATAACAGCTATCAACTTCCCTGCTCTCAGGGTTTGGACGATATTGAGAGTTC
AGCTTCACTCTCTAGCAGTTCCAACACCAATGCTCCAAACATTGGAGGATTCCACACA
GGCCATGCAGGGTATCGCGAAAACAGTTCTAGGATTTTCCCATTTGGTTGGGAGTAG

>CqSPL14 AUR62003425-RA 349
MEWDSKSCAWNGVSKVEFQDNGYHHHLATLAGSSGTGISNMFSVDLKLGRLGDMRDLS
MDTLKNSMPLNMASSSSPSPVLALSKRGRPRNSGAQSTVICSVDGCASDLNQCREYHRRH
KVCERHSKTPVVLVGGKEQRFCQQCSRFHSLEEFDEVKRSCRKRLDGHNRRRRKRQPETFY
MPDSAGGVLSSHTADGMLQYSCPQMHTVNWPTMSQQPDLYFQNNQETIYQPLLNDVAPT
GSRRGGLKVSPDNSGCALSLLSRYSSQSSDIRVGPAMQPTVMSSATAQGSSTSLHLNNSYQL
PCSQGLDDIESSASLSSSSNTNAPNIGGFHTGHAGYRENSSRIFPFGWE*

>*CqSPL15* AUR62042534-RA 2769bp
ATGGAAGAGGTGGGAACACAAGTGGCTTCCCCCTTGTATATACACCAGAACATAGGTG
GGAGGTTTTGTGAGGGTGCTTTGATCGGTGCAAAGCGTAGTCTTTGTTATAATTCTGGT
AGTAATCATCACCAACAGCAACAGCAACACTCACAAATCCATCGTTCTGGGCATGGAT
GGAATCCCAAGGATTGGGAGTGGGATAGTTCCCATTTCTTAGCTAGAAGTAGACCTCT
TGAATCTGATCGGCTTCGTCTGGGTAGTCTTCATGAGGTATCGACTCCTAATAACAAGG
AAGTTGTCAACCCTGTTTTAAGCTCTTCCTTTACCTCGAAGAGCAGCAGCCCTCCTCAT
GATGATCAACAAGGTGGAAATAGTCTTCGATTACAGCTAGGTGGGGTAGGAGACAAT
AATGGCACTGGAACCAGTTTTAACAAAACCAGCGTTGGGAATGTTCAGAGCTCAAAC
TCGACAGAGGAACCAGTTTCCTCTTTAAGGCCCAACAAGAAGGTACGGTCTGGATCTC
CAGGCGGCGGGAACTATCCCATGTGTCAGGTGGATAGTTGTAGCGAGGATCTCTCCAA
ATCTAAGGATTATCACCGACGCCATAAGGTCTGCGAGTTACACAGCAAGGCCACCAA
AGCACTTGTAGGCAAGCAGATGCAGAGGTTCTGCCAACAGTGTAGCAGGTTCCACCC
ACTAGCTGAATTTGATGAAGGTAAAAGAAGTTGTCGAAGAAGACTTGCTGGTCATAAT
AAAAGAAGAAGGAAAACTCAAGCGGAGGACACCACATCTCCGGCCATACAGCCTAC
TGACAGCCACAAGACTGGCTATGGAAATTTGGACATTGTCAACTTGTTGACAGTTTTA
GCACGTGGGCAAGGTGCTTTGGGTCAAAGCAATATGGATGTTGATGCAGCTTCTAGAT
CAACTACCAATCTTCTTGCTGTCCTTTCAGCCACTTTGGCAGCATCAAGTCCAGGTTCT
CTTGCGTTCTTTTCTCAGAAGAGTAATCCTGGCAGTTGCGTGGATAAAAATAAGTCAA
CTACTATGGACAAAGATACTGGCTCAGATGCATGTAAGAAGCCATCTATAGAGTTGCA

GTCATTAGGTGGAGAGAGAAGTAGTTCAAGTTATCAATCTCCAACTGAAGATTCAGAC
AGCCAAGTTCAGGACACTCGAACGAACCTACCGTTGCAGCTGTTTAGTTCATCACCTG
GGGATGATAGTTCGCCTAATCTGGTTACCTCGAGGAGGTATTTCTCTTCTGACAGCAGC
AATCCTACTGAAGAGAGATCACATTCATCGGCTGCACCTGTAACTAGAAAACTGTTTC
CATTGGAAACTGCCTCAGAAAGTGCTAGGCCTGTCAGAATGTCATTTAGTGAGGAGGC
CAATGTAAACGTTGAAGCAAGCCGAACTGAGACCTCTGCTTCTAGGATGACTCTGGA
GCTCTTTACTATGGGAAATAAGGCTGCCAGTAATTCTTTGCAGAACCTGCCACACCAA
GCAGGGTATACTTCTTCGTCTGGTTCTGACCATTCTCCACCAAGTTTCAATTCAGATCCT
CAGAAGGACCGCACTGGCCGAATCATTTTCAAACTGTTTGATAAGGATCCTAGTCAAT
TACCTGGAGCATTGCGCACTCAGATCTACAATTGGCTTTCTAACAGTCCATCAGATATG
GAGAGTTACATTAGACCTGGTTGTGTGGTTCTTTCAATATATGTATCTATGTCATCTGCT
GCCTGGGAACAACTTGAAGAAAGCTTTCTTCAACGAGTTGAGGCATTGGTTCAGGATT
CTGATTTCGAATTCTGGAGAAGTGGGAGGTTTTCAGTGAACATTGGCACGCAGTTAGC
AGTTCATAAGGATGACCCGTTTCGCTTTGTTACCCTTTGGACTGAAGGATTCGCATTTG
CAAACCTTGGAGCACAGAACCTGACTTCTCCTGGTACCAAGATTCATTGTGCATACAT
GGGTGGATACTCATCAAAGGAAGTTTTGAAATCATCTGATCAAGGATTACCATGTGAA
GAGATAAGATTGAATGAATTTAATGTGCATGCTGCAGCTTCTTCTGTTCTAGGCCGTTG
TTTTATTGAGGTAGAAAATGGCGTGAGAGGCAATTGCTTTCCTATAATAATAGCAGATG
CCAAAATTTGTCAGGAGCTTAGGCTCCTTGAGCGTGAGTTTTATGAAGCAAAAGACTC
TGATGTCATCACTGATGATCAGGCCAATATGTTGCAATATCTCTTGACATTTTCCACTGA
AAGAGATTACAGTGCTTTGGTTAAAACACTTTTGGATATATTTGTTGAAGCGGAGTCTA
GAATGGACGGGTTATCGACGGAGTGTGTGGAAGCACTTTCAAACATGCACCTCTTGCA
CAGGGCAGTTAAAAGGAGCTCCAGGAAGATGGTGGACATGCTTGTCCATTATTCTGCA
CCCTGCTCATACGATATTGTAGATGCTCTTACTAATGATCCCATGCAGATTGGACCGCAT
AGCTGGAGGTCTCTTCTAGATGATGGTGAGGTGAGGCAAAGGCCAAGCCTGCCGCTT
GATACAAAACAAAAATCTTGTTCCAAATGTGCTATGAGGAGTTACAGTAGAATGCCTG
GTTCCCATGGTTTTCTTCACCGTCCCTTTATTCATTCTATGCTGACCATTGCTGCAGTATG
TGTTTGTGTCTGCTTGTTCTTCAAAAGTTTGCATGTAAATTCAGTCACTCCTTTCATGTG
GGACAATGTGGATTTTGGGGCTATGTAG

>CqSPL15 AUR62042534-RA 923
MEEVGTQVASPLYIHQNIGGRFCEGALIGAKRSLCYNSGSNHHQQQQHSQIHRSGHGW
NPKDWEWDSSHFLARSRPLESDRLRLGSLHEVSTPNNKEVVNPVLSSSFTSKSSSPPHDDQQ
GGNSLRLQLGGVGDNNGTGTSFNKTSVGNVQSSNSTEEPVSSLRPNKKVRSGSPGGGNYP
MCQVDSCSEDLSKSKDYHRRHKVCELHSKATKALVGKQMQRFCQQCSRFHPLAEFDEGK
RSCRRRLAGHNKRRRKTQAEDTTSPAIQPTDSHKTGYGNLDIVNLLTVLARGQGALGQSN
MDVDAASRSTTNLLAVLSATLAASSPGSLAFFSQKSNPGSCVDKNKSTTMDKDTGSDACK
KPSIELQSLGGERSSSSYQSPTEDSDSQVQDTRTNLPLQLFSSSPGDDSSPNLVTSRRYFSSDSS
NPTEERSHSSAAPVTRKLFPLETASESARPVRMSFSEEANVNVEASRTETSASRMTLELFTMG
NKAASNSLQNLPHQAGYTSSSGSDHSPPSFNSDPQKDRTGRIIFKLFDKDPSQLPGALRTQI
YNWLSNSPSDMESYIRPGCVVLSIYVSMSSAAWEQLEESFLQRVEALVQDSDFEFWRSGRFS
VNIGTQLAVHKDDPFRFVTLWTEGFAFANLGAQNLTSPGTKIHCAYMGGYSSKEVLKSSDQ
GLPCEEIRLNEFNVHAAASSVLGRCFIEVENGVRGNCFPIIIADAKICQELRLLEREFYEAKD
SDVITDDQANMLQYLLTFSTERDYSALVKTLLDIFVEAESRMDGLSTECVEALSNMHLLHR

AVKRSSRKMVDMLVHYSAPCSYDIVDALTNDPMQIGPHSWRSLLDDGEVRQRPSLPLDTK
QKSCSKCAMRSYSRMPGSHGFLHRPFIHSMLTIAAVCVCVCLFFKSLHVNSVTPFMWDNV
DFGAM*

>*CqSPL16* AUR62035190-RA 3321bp
ATGGAAGAGGTGGGAACACAAGTGGCTTCCCCCTTGTATATACACCAAAACATTGGTG
GGAGGTTTTGTGAGGGGGCTTTGATCGGTTCGAAGCGTAGTCTTTGTTATAATTCTGGT
AGTAATCATCACCAACAACAACAGCAACACTCACAAATCCATCGTTCTGGGCATGGAT
GGAATCCCAAGGATTGGGAGTGGGATAGTTCCCATTTCTTAGCTAGAAGTAGACCTCT
TGAATCTGATCGGCTTCGTCTGGGTAGTCTTCATGAGGTATCGACTCCTAATAACAAGG
AAGTTGTCAACCCTGTTTTAAGCTCGTCCTTTACCTCGAAGAGCAGCAGCCCTCCTCAT
GATGATCAACAAAGTGGAAATAGTCTTCGATTACAGCTAGGTGGGGTAGTAGACAATA
ATGGCACTGGAACCAGTTTTAACAAAACCAGCGTTGGGAATGTTCAGAGCTCAAACT
CAACAGAGGATCCGGTTTCCTCTTCAAGGCCCAACAAGAAGGTTCGGTCTGGATCTCC
AGGCGGTGGGAACTATCCCATGTGTCAGGTGGATAGTTGTAGCGAGGATCTCTCCAAA
TCTAAGGATTATCACAGACGCCATAAGGTTTGCGAGTTACACAGCAAGGCCACCAAA
GCACTTGTAGGCAAGCAGATGCAGAGGTTCTGCCAGCAGTGTAGCAGGTTCCACCCA
CTAGCTGAATTTGATGAAGGTAAAAGAAGTTGTCGAAGAAGACTTGCTGGTCATAATA
AAAGAAGAAGGAAAACTCAAGCGGAGGACACCACATCTCCGGCCATACAGCCTACT
GACAGCCACAAGACTGGCTATGGAAATTTGGACATTGTCAACTTGTTGACAGTTTTAG
CACGGGGGCAAGGGAATGCTGAACAGAATGTTCCTCCTTGCCCATCGTTACCTGACAA
AAACCAGCTTATGCAAATACTGAGCAAGATTAATTCATTACCTCTGCCTACGGACATTG
CAGCAAACCCTCATATTCCACCGTGCTCAACCAAAAATGGTTTTGAACAAGGTGCTTT
GGGTCAAAGCAATATGGATGTTGATGCAGCTTCTAGATCAACTACCGATCTTCTTGCTG
TCCTTTCAGCCACTTTGGCAGCATCAAGTCCAGATTCTCTTGCGTTCTTTTCTCAGAAG
AGTAATCCTGCCAGTTGCGTGGATAAAAATAAGTCAACTGATATGGACAAGGATACTG
GCTCAGATGCATGTAAGAAGCCATCTATAGAGTTGCAGTCATTAGGTGGAGAGAGAAG
CAGTTCAAGTTATCAATCTCCAACTGAAGATTCAGACAGCCAAGTTCAGGACACTCGA
ACAAACCTACCGTTGCAGCTGTTTAGTTCATCACCTGGGGATGATAGTTCGCCTAATCT
GGTTACCTCGAGGAGGTATTTCTCGTCTGACAGCAGCAATCCTACTGAAGAGAGATCA
CATTCATCGGCTGCACCTGTAACTAGAAAACTGTTTCCATTGGAAACTGCCTCAGAAA
GTGCTAGGCCTGTCAGAATGTCATTTAGTGAGGAGGCCAATGTAAACGTTGAAGCAAG
CCGAACTGAGACCTCTGCTTCTAGGATGACTCTAGAGCTCTTTACCATGGGAAATAAA
GCTGCCAGTAATTCTTTGCAGAACCTGCCACACCAAGCAGGGTATACTTCTTCGTCTG
GTTCTGACCATTCTCCACCAAGTTTCAATTCAGATCCTCAGAAGGACCGTACCGGCCG
AATCATTTTCAAACTGTTTGATAAGGATCCCAGTCAATTACCTGGAGCATTGCGTACTC
AGATCTACAATTGGCTTTCTAACAGTCCATCAGATATGGAGAGTTACATTAGACCTGGT
TGTGTGGTTCTTTCAATATATGTATCTATGTCATCTGCTGCATGGGAACAACTTGAAGAA
AGCTTTCTTCAACGAGTTGATGCATTGGTTCAGGATTCTGATTTCGAATTCTGGAGAAG
TGGGAGGTTTTCTGTGAACATTGGCAGGCAGTTAGCAGTTCATAAGGATGGAAGGATT
CGCATTTGCAAACCTTGGAGCACAGTGAGTTCTCCCGAGTTGTTTGTGGTTTCTCCTTT
GGCCGTTGTGAGTGGACAAGACACATCTCTTGTCCTTAGAGGCAGGAACCTGACTTCT
CCTGGTACCAAGATTCATTGTGCATACATGGGTGGGTACTCGTCAAAGGAAGTTTTGA
AATCATCTGATCAAGGATTACCATGCGAAGAGATAAGATTGAGTGAATTTAATGTGCAT

GCTGCAGCTGATTCTGTTCTAGGCCGTTGTTTATTGAGGTAGAAAATGGTGTGAGAGG
CAATTGCTTTCCTATTATAATAGCAGATGCCAAAATTTGTCAGGAGCTTAGGCTCCTTG
AGCGCGAGTTTTGTGAGGCAAAAGACTCTGATGTCATCACTGATGATCAGGCCCAATA
TGTTGCAAGTCCTCATTCTCATGAAGCCCTGCACTTCTTAAATGAACTCGGATGGCTTT
TCCAGAGACAAAAATCGTCTGATATTGCTGTTAATGATTTTATGCTTCATCGCTTTCAGT
ATCTCTTGACATTTTCCACTGAAAGAGATTACAGTGCTTTGGTTAAAACACTTTTGGAT
ATATTTGTTGAAGCAGAGTCTACAATGGACGGGTTATCGACGGAGTGTGTGGAAGCAC
TTTCAAACATGCACCTCTTGCACAGGGCAGTTAAAAGGAGCTCCAGGAAGATGGTGG
ACATGCTTGTCCATTATTCTGCACCCTGTGGCAGTGATTCGTCAAAGAAGTACATCTTC
CCTCCTAATCTCCGTGGACCTGGGGGCATCACACCTCTGCATTTGGCTGCTTGTACATC
AGGCTCATACGATATTGTAGATGCTCTTACTAATGATCCCATGCAGATTGGACTGCATA
GCTGGAAATCTCTTCTAGATGATGGTGGTCAGTCTCCATATTCTTATGCTTTGATGAGAA
ATAATCATACACTGAATAGCATGGTGGCTCAAAAGCTGTCTGACAGAAAAAACAAGC
AAGTCTCTGTTACTATTGGAAATGAGATTGTTGAATCCATGGTGCCTGCTGAGGTGAGG
CAAAGGCCAAGCCTGCCACTTGATACAAAACAAAAATCATGTTCCAAATGTGCTATG
AGGAGTTACAGTAGAATGCCTGGTTCCCATGGTTTTCTGCACCGTCCCTTTATTCATTCT
ATGCTGACCATTGCAGCAGTATGTGTTTGTGTCTGCTTGTTCTTCAAAAGTTTGCATGTA
AATTCAGTCACTCCTTTCATGTGGGACAATGTGGATTTTGGGGCTATGTAG

>CqSPL16 AUR62035190-RA 1106
MEEVGTQVASPLYIHQNIGGRFCEGALIGSKRSLCYNSGSNHHQQQQQHSQIHRSGHGWN
PKDWEWDSSHFLARSRPLESDRLRLGSLHEVSTPNNKEVVNPVLSSSFTSKSSSPPHDDQQS
GNSLRLQLGGVVDNNGTGTSFNKTSVGNVQSSNSTEDPVSSSRPNKKVRSGSPGGGNYPM
CQVDSCSEDLSKSKDYHRRHKVCELHSKATKALVGKQMQRFCQQCSRFHPLAEFDEGKRS
CRRRLAGHNKRRRKTQAEDTTSPAIQPTDSHKTGYGNLDIVNLLTVLARGQGNAEQNVPP
CPSLPDKNQLMQILSKINSLPLPTDIAANPHIPPCSTKNGFEQGALGQSNMDVDAASRSTT
DLLAVLSATLAASSPDSLAFFSQKSNPASCVDKNKSTDMDKDTGSDACKKPSIELQSLGGER
SSSSYQSPTEDSDSQVQDTRTNLPLQLFSSSPGDDSSPNLVTSRRYFSSDSSNPTEERSHSSAAP
VTRKLFPLETASESARPVRMSFSEEANVNVEASRTETSASRMTLELFTMGNKAASNSLQNLP
HQAGYTSSSGSDHSPPSFNSDPQKDRTGRIIFKLFDKDPSQLPGALRTQIYNWLSNSPSDMES
YIRPGCVVLSIYVSMSSAAWEQLEESFLQRVDALVQDSDFEFWRSGRFSVNIGRQLAVHKDG
RIRICKPWSTVSSPELFVVSPLAVVSGQDTSLVLRGRNLTSPGTKIHCAYMGGYSSKEVLKSSD
QGLPCEEIRLSEFNVHAAADSVLGRCFIEVENGVRGNCFPIIIADAKICQELRLLEREFCEAK
DSDVITDDQAQYVASPHSHEALHFLNELGWLFQRQKSSDIAVNDFMLHRFQYLLTFSTERD
YSALVKTLLDIFVEAESTMDGLSTECVEALSNMHLLHRAVKRSSRKMVDMLVHYSAPCGSD
SSKKYIFPPNLRGPGGITPLHLAACTSGSYDIVDALTNDPMQIGLHSWKSLLDDGGQSPYSY
ALMRNNHTLNSMVAQKLSDRKNKQVSVTIGNEIVESMVPAEVRQRPSLPLDTKQKSCSKC
AMRSYSRMPGSHGFLHRPFIHSMLTIAAVCVCVCLFFKSLHVNSVTPFMWDNVDFGAM*

>*CqSPL17* AUR62005645-RA 1080bp
ATGGGCTATAAACTTACAAAAAATTACTGTGACTTGGCTGAGTTGAAGAAGGGAGGA
ATTGCTACTATTTCAGCCATTTTTGGGTCCTCTAGCTTTGATACAGAGAAGGCAATAGG
AGAGAGGCTTGTGGATTTGAAACTTGGTAGGTTAGGTGACTTTGGAAAAGGATTAGTG
AACAACTTCAAGGGTCATTCAAATTCTATTATGGACCCATTTTCACCAACAGCTGGATC

ATCAAAAAGGCCCCGAACGCCAGGTACCACTAGCCAAGTGGTTTCGTGCTTAGTGGA
TGGATGCAAGGCCGACCTTAGTAAATGCAGGGACTACCACCGACGCCATAAAGTTTGT
GAGATGCACTCCAAGACCCCAAGAGTTACAATTGGGGGCAATGAACAACGCTTTTGT
CAGCAATGCAGCAGGTTTCACCCACTAGGGGAATTCGATGAGGGGAAGCGGAGTTGT
AGAAAACGTCTTGAAGGACATAATCGTCGTAGAAGGAAGCCTCAGCCTGAACCCCTA
CCTGTGAATCAAGGAAATTTCTTTTCTGCTAGCCAAGGTAGTAGATTTTTGGCATTCAG
CAACCAACCAATAATTCCAGCTACATCAGTGGTGACAACTGCTTGGTCTGGAGCTGTT
AAATCTGAGAGCAACCCGACACTCTACAACAGCGCGGCACCTAGTTCATACTCTCAG
GCTTACAGAGGCAGACAGTTCCCATTCTTACAAGTTCCTGAATCCCCACTTACTGGAG
TTTCTTCAGCTGTGATTGACTCGGATCGTGCTCTCTCTCTTCTGTCATCAAATGCTGAGA
CTCCCGAGATGGGTTTGGGCCATACTTTCCATCATCCGAGCCTAGTAAACCCTACTCGG
GCAATGCTTTCAAGCCTGCACTACAACAGCGTTCCTTCTCAGTACCCAGGCTCCTCCC
AAGGCCATGGTATGGAAGGACATCCTACAGGTTCTGGACTGTTCTCGGACTTACGGAG
CAGTAACAGCCTCTGCCAGGATGTTTTCCAGAATAATACTGGTGCATCTTCTACTAGTG
GAGGCCATCAAACACTTTCCTTCTCTTGGGAGTAA

>CqSPL17 AUR62005645-RA 359
MGYKLTKNYCDLAELKKGGIATISAIFGSSSFDTEKAIGERLVDLKLGRLGDFGKGLVNNFK
GHSNSIMDPFSPTAGSSKRPRTPGTTSQVVSCLVDGCKADLSKCRDYHRRHKVCEMHSKTP
RVTIGGNEQRFCQQCSRFHPLGEFDEGKRSCRKRLEGHNRRRRKPQPEPLPVNQGNFFSAS
QGSRFLAFSNQPIIPATSVVTTAWSGAVKSESNPTLYNSAAPSSYSQAYRGRQFPFLQVPESPL
TGVSSAVIDSDRALSLLSSNAETPEMGLGHTFHHPSLVNPTRAMLSSLHYNSVPSQYPGSSQ
GHGMEGHPTGSGLFSDLRSSNSLCQDVFQNNTGASSTSGGHQTLSFSWE*

>*CqSPL18* AUR62028905-RA 954bp
ATGGGCTATAAACTTACAAAAAATTACTGTGACTTGGCTGAGTTGAAGAAGGGAGGA
ATTGCTACTATTTCAGCCATTTTTGGGTCCTCTAGCTTTGATACAGAGAAGGCAATAGG
AGAGAGGCTTGTGGATTTGAAACTTGGTAGGTTAGGTGACATTGGAAAAGGATTAGTG
AACAACTTCAAGGGTCATTCACATTCTATTATGGACCCATTTTCACCAACAGCTGGATC
ATCAAAAAGGCCCCGAACACCAGGTACCAGTACCCAAGTGGTTTCGTGCTTAGTGGA
CGGATGCAAGGCCGACCTTAGTAAATGCAGGGACTACCACCGACGCCATAAAGTTTG
TGAGATGCACTCCAAGACCCCAAGAGTTACAATTGGGGGTAATGAACAACGCTTTTGT
CAGCAATGTAGCAGGTTTCACTCACTAGGGGAATTCGATGAGGGGAAGCGGAGTTGT
AGGAAACGTCTTGAAGGACATAATCGTCGTAGAAGGAAGCCTCAGCCTGAACCCCTA
CCTGTGAATCATGGAAATTTCTTTTCTGCTAGCCAAGAGAAGCAAGCACAAGCAGAAT
TGTCTTTACCTGACCCTCAACTTCCCTTGATTGTTGAACTGAAACCTTTCCTGAACCAA
GTGATTGACTCGGATCGTGCTCTCTCTCTTCTGTCATCAAATGCTGAGACTCCCGAGAT
GGGTTTGGGTCGTACCTTCCATCATCCGAGCCCTATAAACCCTACTCGGCCAATGCTTT
CAAGCCTGCACTTCAACAGCGTTCCTTCTCAGTACTCAGGCTCCTCCCAAGGCCATGG
TATGGAAGGACATCCTACAGGTTCTGGACTGTTCTCGGACTTACGGAGCAGTAACAGC
CTCTGCCAGGATGTTTTCCAGAATGATACTGGTGCATCTTCTACTAGTGGAGGTCATCA
AACACTTTCCTTCTCTTGGGAGTAA

>CqSPL18 AUR62028905-RA 317

MGYKLTKNYCDLAELKKGGIATISAIFGSSSFDTEKAIGERLVDLKLGRLGDIGKGLVNNFK
GHSHSIMDPFSPTAGSSKRPRTPGTSTQVVSCLVDGCKADLSKCRDYHRRHKVCEMHSKTP
RVTIGGNEQRFCQQCSRFHSLGEFDEGKRSCRKRLEGHNRRRRKPQPEPLPVNHGNFFSAS
QEKQAQAELSLPDPQLPLIVELKPFLNQVIDSDRALSLLSSNAETPEMGLGRTFHHPSPINPT
RPMLSSLHFNSVPSQYSGSSQGHGMEGHPTGSGLFSDLRSSNSLCQDVFQNDTGASSTSGG
HQTLSFSWE*

>*CqSPL19* AUR62003075-RA 1500bp
ATGTTTGCTTCCTCGGTTGAGAGGAGGTGGCATTTCCTTGGTGTAGAGGAAATGGGTGT
GTTATTGAGGTTAGTTGAGATCTCAATGCTTGCATTGCCTTCTTGGTGCGTAGAAGGTG
AGGAGATGGTGTTCCTTGGTGTAGAGGACATGTGTTGCACACATGAGCATTGTGTGTG
GGAAAGCAAAGGATGTTTTGTTCGGGTGGGAGCATGGTCAATTGTGATGGATTCTTGG
AAACATATGGGATGTGAAATTAAAACCATGTTTAATGTTGAAAGTTACCCATTAATTTC
TAATACTCAAGCACTTGAGAGAATGGATTTTATGGATTTGGGGTTTCCTGATGATATTAG
GAAATCATATTCAATTTCTAGTGAACCAAGTGGGGAGGTTTTCAGTGATGAAATTGGC
ACAGATTATCCCCAATTTGGTGAAGAATCTAATCATCAACAAGATTTTTCACTTGTTGA
TTTGAAGTTGGGTAGGTTGGAAGATGGTGGGATTGATAAAGATAGTGAATTGTCAAAA
ATTAATGCAGCTGAATCATCTGTTATGCTGTCTTCTCCGGCAAAGAAAGCTCGGTCTTC
GATGTCGATTAATCGATCTTCCAATTGTCAAGTTCTTGGTTGTAACAAGGATCTTAGCTC
TTTTAAGAGCTACTATAAGCGGCATAAGGTCTGTGATGTTCATACAAAGACACCTAAA
GTTATTGTTGATGGGATTGAGCAAAGATTTTGTCAACAATGTAGCAGGTTTCATCTATTG
GCTGAATTTGACGACATCAAACGTAGTTGTCGTAGGCGTCTTTCTGCTCATAATAAGCG
TCGAAGGAAGCCTCAGCTTGCTTCTGAACCAGGTAGTGGGATAATTGACTTTTCCTCAT
CACTTGCTTTTCCTGAAGTGATGCCTAGTGTTTATCTTGGTCCGAGTAAGTATGAAGAG
GGGATTGATAATTCCATCAAGAGCTCAAATTTCCTTCTCCAAATGGGATGTGTAGGAG
AGAAAATAAATAAGTTTCCTGCAAATGTTAAGGACGACATTGCTGGCTTTGCAAAACC
GACTAATGAAACTTTATCCAGCATTCAGCAAACTAGTCGTGCTCTCTCTCTTCTGTCAG
CTCAATCACATAGTATATCAAGCCAGTTGCTGGAAACTGCAAGCGCGACACTGACCA
ACAGAGGCAACCATGCAGATTCGAATCCTGGGCATTCTCTTAACCATTCAGCTGGCAT
TTTTAATAAAGTTTCAGCAAGTGGCCCTCATGAACCTGGAATCTATTCAATGGACGCTG
ACGAGGATGGACCCACCATGATCAATCATAACAATAGCAACACTGTTGGTCTTCATCT
CCCAAGTGATGGCATTTCAAGGCCGGGTTTAAGGAACACTGATTGTCACCATCCCAAG
GAACGTGGCATCATCCTTAACTTGCTTCAACTGTCTTCGAATCTTCAAAGAGTTGAGCA
GCAAAAACACTCAACTGAAAATGAAGCAGGATAA

>CqSPL19 AUR62003075-RA 499
MFASSVERRWHFLGVEEMGVLLRLVEISMLALPSWCVEGEEMVFLGVEDMCCTHEHCVW
ESKGCFVRVGAWSIVMDSWKHMGCEIKTMFNVESYPLISNTQALERMDFMDLGFPDDIRK
SYSISSEPSGEVFSDEIGTDYPQFGEESNHQQDFSLVDLKLGRLEDGGIDKDSELSKINAAESS
VMLSSPAKKARSSMSINRSSNCQVLGCNKDLSSFKSYYKRHKVCDVHTKTPKVIVDGIEQRF
CQQCSRFHLLAEFDDIKRSCRRRLSAHNKRRRKPQLASEPGSGIIDFSSSLAFPEVMPSVYLG
PSKYEEGIDNSIKSSNFLLQMGCVGEKINKFPANVKDDIAGFAKPTNETLSSIQQTSRALSLLS
AQSHSISSQLLETASATLTNRGNHADSNPGHSLNHSAGIFNKVSASGPHEPGIYSMDADED

GPTMINHNNSNTVGLHLPSDGISRPGLRNTDCHHPKERGIILNLLQLSSNLQRVEQQKHST
ENEAG*

>*CqSPL20* AUR62007890-RA 1272bp
ATGGATTCTTGGAAACATATGGGATGTGAATTTAAACCCATGTTTAATGGTGAAACTTA
CCCATTAATTTCTAATACTCAAGCACTTGAGAGAATGGATTTAATGGATTTGGGGTTTG
CTGATGATATTAGGAAATCATATTCAATTTCTAGTGAACCAAGTGGGGAGGTTTTTAGT
GATGAAATTGGTACAGATTATCCCCAATTTGGTGAAGAATCTAATCATCAACAAGATTT
TCCACTTGTTGATTTGAAGTTGGGTAGGTTGGAAGATGGTGGAATTGATAAAGATAGTG
AATTTTCAAAGTTTAATGCAGCTGAATCATCTGTTAGGCTGTCTTCTCCGGCAAAGAAA
GCTCGGTCTTCGATGTCGAGTAATCGATCTTCCAATTGTCAAGTTCTTGGTTGTAACAA
GGATCTTAGCTCTTTCAAGAGCTACTATAAGCGGCATAAGGTCTGTGATGTTCATACAA
AGACTCCTAAAGTTATTGTTGATGGGATTGAGAAAAGATTTTGTCAGCAATGTAGCAG
GTTTCATCTATTGGCTGAATTTGACGACACCAAACGTAGTTGTCGTAGGCGTCTTTCTG
CTCATAATAAACGTCGAAGGAAGCCTCAGCTTGCTTCTGAACCAGGTAGTGGGATAAT
TGACTTTTCCTCATCACTTGCTTTTCCAGAAGTGATGCCTAGTGTTTATCTTGGTCCGAG
TAAGTATGAAGAGGGGATTGATAATTCCATCAAGAGCTCAAATTTCCTTCTTCAAATGG
GATGTGTAGGAGAGAAAATAAATAAGTTTCCTGCAAATGTTAAGGACGACATTGCTGG
CTTTGCAAAACCGACTAATGAGACTTTATCTAACATTCAGCAAACTAGTCGTGCTCTCT
CTCTTCTGTCAGCTCAATCACATAGTATATCAAGCCAATTGCTGGAAACTGCAAGTGCG
ACACTGACCAACAGAGGCAACCATGCAGATTCAAATCCCGGGCATTCTCTTAACCATT
CAGCTGGCATTTTTAATAAAGTTTCAGCAAGTGGCCCTCATGAACCTGGAATCTATTCA
ATGGAAGAGGATGGACCCACCATGATCAATCCTAACAATAGCAGCACTGTTGGTCTTC
ATCTCCCAAATGATGGCATTTCAAGGCCGGGTTTAAGAAACACTGATTGTCACCATCC
CAAGGAACGTGGTATCATCCTTAACTTGCTTCAATTGTCTTCGAATCTTCAAAGAGTTG
AGCAGCAAAAACACTCAACTGAAAATGAAGCAGGATAA

>CqSPL20 AUR62007890-RA 423
MDSWKHMGCEFKPMFNGETYPLISNTQALERMDLMDLGFADDIRKSYSISSEPSGEVFSDEI
GTDYPQFGEESNHQQDFPLVDLKLGRLEDGGIDKDSEFSKFNAAESSVRLSSPAKKARSSMS
SNRSSNCQVLGCNKDLSSFKSYYKRHKVCDVHTKTPKVIVDGIEKRFCQQCSRFHLLAEFD
DTKRSCRRRLSAHNKRRRKPQLASEPGSGIIDFSSSLAFPEVMPSVYLGPSKYEEGIDNSIKSS
NFLLQMGCVGEKINKFPANVKDDIAGFAKPTNETLSNIQQTSRALSLLSAQSHSISSQLLETA
SATLTNRGNHADSNPGHSLNHSAGIFNKVSASGPHEPGIYSMEEDGPTMINPNNSSTVGLH
LPNDGISRPGLRNTDCHHPKERGIILNLLQLSSNLQRVEQQKHSTENEAG*

>*CqSPL21* AUR62042853-RA 2151bp
ATGGATTTACCTCCGTTGACTGGCGGAGGTGAAGAATCGGGTGCGCCATTTGAGTGGA
GTGATCTATTTGACTTTACAATCGACGATCAATTACTGCTTAATCTGGATGTTTCCGATC
ACCCGAAGGAGCAGCCACCGGTGCTACTACCGCCGGTAACGAACGCGGAGGATAATG
GAAAGACCGTTGCGGTAAATGGAGGTGAATCAGGGTCGTCCGATCGGGTGCGAAAGC
GAGACCCGAGGATGATTTGCAAGAATTTTTTGGCGGGGAGGGTGCCGTGCGCTTGTCC
AGAGTTGGATGCATTGATGGCGGAGGAGGAGGAAGAAGAGGCCGGTCCTGGAAAGA
AGCGGCCGAGGATGGGGCGGACACCGGGTGTAGCGAAGTGTCAGGTGCCCGGGTGT

GAGGTCGATATTCGTGAGCTCAAAGGGTATCACCGGCGACATAGGGTTTGTTTGGTGT
GTGCTAATGCTACCTCGGTTGTGATTGAGGATTTTGATGAAGGAAAACGTAGTTGTAGA
CGTAAATTAGAGCGCCACAACAATAGGCGGCGAAGGAAGTCTGCTGATTACAGAGGA
ACTGTTGAAAAGGAGCCTCAGGGTGATGTGCAGACAGAAGATGTTTTTCTGATGGAG
AAGCAGGGAAAGAGAATGCATGGTCAGGTGGCCAAATTGAGAAGGAAGATTCCAAG
GATAAAACTTTGTCAAACCTTTGCTCTGCTCTGGAATCTCAGAACATTGAGAGTGACA
GCAATTTAACTTTTACTCCAGTGGATAAAGTAGTAGATAATTTGGAGCGAGAATATTCC
CCACCTTCTGATACTAAGAGTGCTTACACATCTGCGTGCCCTACAGGTCGTATCTCTTTT
AAACTCTACGACTGGAACCCTGCAGAATTCCCTCGACGACTACGGCATCAAATATTCC
AATGGTTGGCCAGCATGCCTGTTGAGTTGGAGGGCTATATCCGTCCTGGTTGTACAATA
TTGACTGTATTTGTTTCAATGCCACAATATATGTGGGTAAAGCTGTTTGAAGACCCTGTT
TCTTATGTACAAAACTCTGTTGGACATGGAGGAATACTGTCTGGGAGAGGCGCTGCCT
TGGTTTACTTGAACGATTTGAGATTTCGTGTTATGAGAGAAGGAGCTTCCGTGATGAAA
GTGAAGGTTGCTGTTCGGGCCCCAAGACTTCACTACATCTATCCTCCTTGTTTTGAGGC
TGGAAAACCAATGGATTTTGTTGCCTGTGGAAGCAATTTACTTCAGCCAAAATTTCGGT
CTCTCGTATCGTTTGCGGGAAAGTATTTGGCTCATGATTACTATGTCGCTTTTCCACGTG
GAAAAGAAGACAAGCCTGCCATTGATTATGATTATCAGCTCTGCAGAATATATGTGCC
ACACACTGAACCAAGTTACTTTGGTCCTGCGTTTGTTGAGGTCGAGAATGAGTGTGGT
CTATCCAATTTCATACCTATACTCATTGGAGATGCGCATGTCTGTTCCGAAATAAAGTTG
ATACATCAGAAGTATGATTGTTCGAATTGTAGAAAAAAGTCGCAATGTATGCCAAGTG
GTTCTTCATATGGAACTTGCGAAGTTTCATGTTCAAGACAAGCTGCATTGTCTGAATTT
ATGCTGGATGTTGCATGGTTGCTTAAGCAGCCTTGTTCTGAGAAGCTTCACTGCATATT
AACATCTTCTCAGATTCAGAGATACAATTGCTTGATAAACGCTCACAACAAAGAATTG
ATCTTGAAGCAGGGTGGGGAATGTGTCTCAAATGTTTACACATGTTTGGGAAGTTTCTC
TCGTGACGACATGCTATCTGTTGATCATACTGCTTGTCAGGTTACAAAATTGAGAGCAG
AGATGAACTGCACATTTTTGGAGCATTCAGATTCTCAGGGTGAAGGGGAATCTATTTC
ACTTATAAACAGGGAAGTTGCAATGAATGTTAATGACAGCAGAGATTGGCCAAAGAA
CTCATGTCATAGTGTAATTTCAAAGAAATTCACCAGTACTCGGCCTTTCATTTATGCCAT
TGCTGTAGTTGCTGTTTGCTTTGGTGTATGTGCAGTTGTCTTGCACCCTTACAAAGTTAC
CAAATTTGCAGTTACTATCAGAAGGTGTGTATTTGACAATTCTTCATAG

>CqSPL21 AUR62042853-RA 716
MDLPPLTGGGEESGAPFEWSDLFDFTIDDQLLLNLDVSDHPKEQPPVLLPPVTNAEDNGKT
VAVNGGESGSSDRVRKRDPRMICKNFLAGRVPCACPELDALMAEEEEEEAGPGKKRPRMG
RTPGVAKCQVPGCEVDIRELKGYHRRHRVCLVCANATSVVIEDFDEGKRSCRRKLERHNN
RRRRKSADYRGTVEKEPQGDVQTEDVFSDGEAGKENAWSGGQIEKEDSKDKTLSNLCSAL
ESQNIESDSNLTFTPVDKVVDNLEREYSPPSDTKSAYTSACPTGRISFKLYDWNPAEFPRRLR
HQIFQWLASMPVELEGYIRPGCTILTVFVSMPQYMWVKLFEDPVSYVQNSVGHGGILSGRG
AALVYLNDLRFRVMREGASVMKVKVAVRAPRLHYIYPPCFEAGKPMDFVACGSNLLQPKF
RSLVSFAGKYLAHDYYVAFPRGKEDKPAIDYDYQLCRIYVPHTEPSYFGPAFVEVENECGLS
NFIPILIGDAHVCSEIKLIHQKYDCSNCRKKSQCMPSGSSYGTCEVSCSRQAALSEFMLDVA
WLLKQPCSEKLHCILTSSQIQRYNCLINAHNKELILKQGGECVSNVYTCLGSFSRDDMLSVD
HTACQVTKLRAEMNCTFLEHSDSQGEGESISLINREVAMNVNDSRDWPKNSCHSVISKKFT
STRPFIYAIAVVAVCFGVCAVVLHPYKVTKFAVTIRRCVFDNSS

>*CqSPL22*   AUR62042654-RA   2124bp

ATGGATTTACCTCCGTTGACTGGCGGAGGTGAAGAATCGGGTGCGCCATTTGAGTGGA
GTGATCTCTTTGACTTTACAATCGACGATCAATTACTGCTTAATCTGGATGTTTCCGATC
AGCCGAAGGAGCAGCCACCGGTGCTACTACCGCCGGTGACGAACGCGGAGGATAAT
GGAAAGACCGTTGCGGTGAATGGAGGTGAATCGGGGTCGTCTGATCGGGTGCGAAAG
CGAGACCCGAGGATGATTTGCGAGAACTTTTTAGCGGGGCGGGTGCCGTGCGCTTGTC
CGGAGTTGGATGCATTGATGGCGGAGGAGGAGGAAGAAGAGACCGGGCCCGGAAAG
AAGCGGCCGAGGATGGGGCGGACACCGGGTGTAGCGAAGTGTCAGGTGCCCGGGTG
TGAGGTCGATATTCGTGAGCTCAAAGGGTTTCACGTGCTGTCAGATTTTGATGAAGGA
AAACGTAGTTGTAGACGTAAATTAGAGCGCCACAACAATAGGCGGCGAAGGAAGTCT
GCTGATTACAGAGGAACTGTTGAAAAAGAACCTCAGGGTGATGTGCAGACAGAAGAT
GTCTTTTCTGATGGAGAAGCAGGGAAAGAGAATGCATGGTCAAGTGGTCAAATAGCT
GAGAAAGAAGATTCTAAAGATAAAACTTTGTCAAACCTTTGCTCTGCTCTGGAATCTC
AGAACATTGAGAGTGACAGCAATTTAACTTTTACTCCAGTGGATAAAGTAATAGATAA
TTTGGAGCGAGAATATTCCCCACCTTCTGATACTAAGAGTGCTTACTCATCTGCGTGCC
CTACAGGTCGTATCTCTTTTAAACTCTATGACTGGAACCCTGCAGAATTCCCTCGACGA
CTTCGGCACCAAATATTCCAATGGTTGGCCAGCATGCCTGTTGAGTTGGAGGGCTATAT
CCGTCCTGGTTGTACAATATTGACTATATTTGTTTCAATGCCACAATATATGTGGGTAAA
GCTGTTTGAAGACCCTGTTTCTTATGTACAAAACTCTGTTGGACATGGAGGAATACTGT
CTGGCAGAGGCGCTGCCTTGGTTTACTTGAATGATTTGAGTTTTCGTGTTATGAGAGAA
GGAACTTCCGTGATGAAAGTGAAGGTTGCTGTTCGGGCCCCAAAACTTCACTATGTCT
ATCCTCCTTGTTTTGAGGCTGGAAAACCAATGGATTTTGTTGCCTGTGGAAGCAATTTA
CTTCAGCCAAAATTTCGGTCTCTCGTATCATTTGCGGGAAAGTATTTGGCTCATGATTAC
TATGTCGCTTTTCCACGTGGAAAAGAAGACAAGCCTGCCATTGATTATGATTATCAGTT
CTGCAGAATATATGTGCCACACACTGAACCAAGTTACTTTGGTCCTGCGTTTGTTGAGG
TCGAGAATGAGTGTGGTCTATCCAATTTCATACCTATTCTCATTGGAGATGAGCATGTCT
GTTCTGAAATAAAGATGATACATCAGAAGTATGATTGCTCGAATTGTAGAAAAAAGTT
GCAATGTATACCAAGTGGTTCTTCGTATGGAACTTGTGAAGTTTCATGTTCAAGACAAG
CTGCACTGTCTGAATTTATGCTGGATGTTGCATGGTTGCTTAAGCAGCCTTGTTCTGAG
AAGCTTAACTGCATATTAACAAGGTTGCTGAAGGAATATATTGCTCAGGCTAATGATTT
TCTTCGCCAAAGCGCTTACAACAAAGAGTTGATTCTGAAGCAGGGTGGGGAATGTGT
CTCAAATGTTGACACATGTTTGGGAAGTTTCCCTCATGACGACTTGCTGTCTGTTGATC
ATACTGCTTGTCAGGTTACAAAATTGAGAGCAGAGACGAACCACACATTTTTGGAACA
TTCAGATTCTCGGGGTGAAGGGGAATCTGTTTCACTTATAAATAGGGAAGTTGCAATG
AATGTTAATGACAACAGAGATTGGCCAAAGAAGTCGTGTCACAACATAATCTCAAAG
AAATTCACCAGTACTCGGCCTTTCATTTATGCCATTGCTTTAGTTGCTGTTTGCTTTGGT
GTATGTGCAGTTGTCTTGCACCCTTACAAAGTTACCAAATTTGCAGTAACTATCAGAAG
GTGTGTATTTGACGATTCTTCATAG

>CqSPL22   AUR62042654-RA   707

MDLPPLTGGGEESGAPFEWSDLFDFTIDDQLLLNLDVSDQPKEQPPVLLPPVTNAEDNGKT
VAVNGGESGSSDRVRKRDPRMICENFLAGRVPCACPELDALMAEEEEEETGPGKKRPRMG
RTPGVAKCQVPGCEVDIRELKGFHVLSDFDEGKRSCRRKLERHNNRRRRKSADYRGTVEK

EPQGDVQTEDVFSDGEAGKENAWSSGQIAEKEDSKDKTLSNLCSALESQNIESDSNLTFTPV
DKVIDNLEREYSPPSDTKSAYSSACPTGRISFKLYDWNPAEFPRRLRHQIFQWLASMPVELEG
YIRPGCTILTIFVSMPQYMWVKLFEDPVSYVQNSVGHGGILSGRGAALVYLNDLSFRVMRE
GTSVMKVKVAVRAPKLHYVYPPCFEAGKPMDFVACGSNLLQPKFRSLVSFAGKYLAHDYY
VAFPRGKEDKPAIDYDYQFCRIYVPHTEPSYFGPAFVEVENECGLSNFIPILIGDEHVCSEIKM
IHQKYDCSNCRKKLQCIPSGSSYGTCEVSCSRQAALSEFMLDVAWLLKQPCSEKLNCILTRL
LKEYIAQANDFLRQSAYNKELILKQGGECVSNVDTCLGSFPHDDLLSVDHTACQVTKLRAE
TNHTFLEHSDSRGEGESVSLINREVAMNVNDNRDWPKKSCHNIISKKFTSTRPFIYAIALVAV
CFGVCAVVLHPYKVTKFAVTIRRCVFDDSS*

Supplementary file S2
***Cqu-miR156/7* precursor sequences**
>*Cqu-MIR156a*
TTGACAGAAGAGAGTGAGCACAGAAAGGCAGAAATGGTATACATAGCAAACCCAAA
CCAACACCAGAAATCATACATTTAATTTCCAAAGATCGAAACATAGAGTTGGGGTTGA
AGATTATTGGTATACTATTCCTTTTTTGTGCTCACTGCTCTTTCTGTCAGCT

>*Cqu-MIR156b*
TGACAGAAGAGAGTGAGCACACATGGTGCTTTTCTTGCATCATTTTTATGCCTGAAGCT
ATGCGTGCTTACTCTCTGTCTGTCTC

>*Cqu-MIR156c*
TTGACAGAAGAGAGTGAGCACAGAAAGGCAGAAATGGTATATATATACATATATATAT
ATAGCAATTAGCAAACCCAAACCAACACCAGAAAATCATACATTACATTTAATCTCCG
AAGTTGAAAACATCGGAAGTCGAGGTTGAAGATTATTGGTATACTATTCCTTTTTTGTG
CTCACTGCTCTTTCTGTCAGCT

>*Cqu-MIR156d*
TGACAGAAGAGAGTGAGCACACATGGTTCTTTTCTTGCATCTTTTCTTATGCTTGAAGC
TATGCGTGCTTACTCTCTGTCTGTCTC

>*Cqu-MIR156e*
TGACAGAAGAGAAAGAGCACAACCCATCATTTGCTAAAGAAAAGCCTTTACCTGTGT
TGGGAGTGTGCTTTCTCTTCTTCTGTCAAC

>*Cqu-MIR157a*
CTGACAGAAGATAGAGAGCACTGAGGATGATATGCAGATTAAATTTGCTGCATCTCAA
CTCCTTTGTGCTCTCTATGTTCTGTCACC

>*Cqu-MIR157b*
CTGACAGAAGATAGAGAGCACAGATGGATGAAATGTTTGAAATTTGCATTTTATTACC
TTTGTGCTCTCTATCTCTGTCATC

>*Cqu-MIR157c*
CTGACAGAAGATAGAGAGCACTGAGGATGATATGCAGATTACATATGCTGCATCTCAA
CTCCTTTGTGCTCTCTATCTTCTGTCATC

>*Cqu-MIR157d*
TTGACAGAAGATAGGGAGCACAGATGGGTGAAATGCCCATGAAGTTGCACCTCACTT
CCCTTTGTGCTCCTATCTTCTGTCACC

>*Cqu-MIR157e*
CTGACAGAAGATAGAGAGCACTGAGGATGATATGCAGATTAAATTTGCTGCATCTCAA
CTCCTTTGTGCTCTCTATGTTCTGTCACC

>*Cqu-MIR157f*
CTGACAGAAGATAGAGAGCACAGATGGATGAAATGTTTGAAATTTGCATTTTATTACC
TTTGTGCTCTCTATCTCTGTCATC

>*Cqu-MIR157g*
CTGACAGAAGATAGAGAGCACTGAGGATGATATGCAGATTACATATGCTGCATCTCAA
CTCCTTTGTGCTCTCTATCTTCTGTCATC

>*Cqu-MIR157h*
TTGACAGAAGATAGGGAGCACAGATGGGTGAAATGCCCATGAAGTTGCACCTCACTT
CCCTTTGTGCTCCCTATCTTCTGTCACC

>*Cqu-MIR157i*
CTGACAGAAGATAGAGAGCACAGATGGATGAAATGTTTGAAATTTGCATTTCATTACC
TTTGTGCTCTCTATCTTCTGTCATC

>*Cqu-MIR157j*
CTGACAGAAGATAGAGAGCACTAAGGATGATATACAGATTACATATGCTGCATCTCAA
CTCCTTTGTGCTCTCTATCTTCTGTCATC

>*Cqu-MIR157k*
TTGACAGAAGATAGAGAGCACAGAGGATGAAGTGCATTGGGATATTTGGATTTGGGGT
ACTACTTAAAGCTTAGCTTGCTATCATTATTATTATTACTGCTATTGCACTTCACTTTCCT
TCGTGCTCTCTGTGCTTCTGTCATC

>*Cqu-MIR157l*
TTGACAGAAGATAGAGAGCACAGAGGATGAAGTGCATGGGGATATTTGGATTTGGG
GGTACTTAAGCTATCATTATTATTATTATTACTGCTGCACTTCACTTTCCTTCGTGCTCT
CTGTGCTTCTGTCATC

>*Cqu-MIR157m*

TGACAGAAGATAGAGAGCACTAAGGATGATATGCAGATTAAATTTGCTGCATCTCAAC
TCCTTTGTGCTCTCTATCTTCTGTTGT

Supplementary File S3

3.0 kb promoter sequences of *CqSPL* genes

>*CqSPL1*

TTTTAATTGGACGAAAAACGTTAATGGGTAGTGTCACCCAAACATTTTCAAACTTGCG
GGGAGTTCTAAGCGAATAAAAAAAAAAAGGGTAACTGTTCACAAATAACCTTATCCTT
AGGTAGGGTAGTTCTACACAATTTTCTAAACAATATTTCAAACTATACATATAAGAAA
TGAATAAAAATATACAATAACAATATTTCATTGATTTGTTGTGGATTACAATTCTCTTT
TCATTACTCTCTCAAAAGGTTAAAATTTTGAAATGTGTTTATAATACGTAGCCTTGTCG
ACTTATTTCATATTATATGCTTATAATGCATGTTTGACAACAGGGAGATGAAGTCTTTG
TGACTTAGGGCTTGTTCTGTTCAGTTTAATTTTAGTTCAATATAGTTTAGTTAAAATCA
GTTCAGTTCAATTCAGTAAATCGGGACAGGCCCTTATTACATTTGAGTCTTGTTAAAC
CCTAAGAACAACCTTTTACATATAGAGAGTTATATAGTAATAAACAAGATGACTATA
AATTACAGCGCATTTTGTGTGCGGCTGACCACATCATATGACCACTTATAAAAGAATA
AAGTAATTCTTATAACATTGACTAGGTAACCTTTATAATATCGACCAAGTAACCCCTA
AAACACTACCAAATAACCCCTAAACGAGTAACCCCTATAACCCTAACCAAATAATCC
CGATCACATAGTTGTGTATGGTTAAGTTCATGTAAAAAAAAAAAAAAAATTATTTCTAA
AAAAAAAAAAAAAAAAAAAAAAAAAAAACCACACATATTGTATGAATATTACTAGCT
GTCCTTACATAATCCCCCCAACCAATAATTCTCTTTCACCTGTCTTTCTCTCTCTTCAGC
TTCACTCCTCTCTCTCTCCCTTTCATGGCGTCTTATTGAAATTGGTGAAAAGTGGAACA
CTAATTATTAAATAGACCCAGATTTTTTTTTTTTTTTAATTTTTTAAAAAAGGGTAAGCT
TTCTTTGAGCTTTTCTTACCTAAAATTGATGAGATTTATAGAGAGAGAAAGAAAAGAT
TATTATTGTTGGCTAAAAGGTATGAACTTCCAGACTACTTATTTACCCTTTTTTTTTTTT
TTTTTTACTAAATTTGGGCTGTTCTTCTTCTCTTCTAATTAATCCAACTAATTTAATATT
ATTTTGATTCTGATTCAGTACTTTTTTAAGTATATATTTATGAGTAATTTCTGGGTTATT
TATATTATTCTCCTGTGTTTATTCTGCTAGTTCGTTTTTTTTTTTTCAGTGCAGATTGATA
AGATTGAGATAGTTACTTATTTAGGCTAAGCATAATTTTATGATTTTATTTCTTGTACT
GGGTATTTTTTGCAATTTTGGTCATGATTTTCCCAGAAAAAAAGTTTTAATTTTTTATTC
ATTGATTTCTGTGATTTGGGTTGTTATACTTGTATTTTTCTGGGCTAGTGAACATGAAA
ACGGTGTCGTTTCGCTGCTTATTTTATTTTATTTTTGCGAGCTAATACAGTTTATTTTTTT
TTTTAAAAAAACAGTTAAGCCCTTGAAGTAGAGCAAGAGGCAAAAACCCAGAATTT
GGGTTTTTGTAAATCTTTTATATTAACGATGATTTGCGTGAACGTGTGATGGGTTTTTA
TTCTCTCCAAAAGCTGGGAACTTGACTTTTTGACTAGTATTTTTTAAGAAATAAAATGT
TGGGATTATGGGAAAGATCATACTCACATTGTAATGTGTTTAATATTATAATTTTTATT
AGTATATACTTGTAAAAAAATAAATGGGAATCCACTTTGTTCATATTTAAAATTTGTC
AATTGGGTATTTCAAGAAAGGGTAAATTGTTTGCTTAATATTTTGATGGGTCATTAAG
CATTAAAAATCTAGTAGAGTTTTTGGGAAGATGGGGTCATTTTAGATCTGCAATGATT
TGGTGGGCATCCTGTTTTAAAAGGGGTTTTTGGGTTAAAATTTAGTAGCGTTACATCTC
ACTTTAATAGACCACATTGCGCTTGCTTTCGTATTCGAGAGTAGTAAAAGTCGAGTAA
AGGTAAAAACTATCTATTCATTATAGGGTGGCTAGTTGCCAGTCTTGCCACAAATAAT
TGGAGAGAGAGAAAGTGAAGGTTATGGGTGGTTGTATGTTATGAAGAAATGAAACA

AGCTAGGCAACTCTATTCTATAAAATGAGGTTAAATTAGTTACTTTCCAAATAGGTTA
ATTGGGGGATGGTAAAATTTCTATAAATGGGATTATAAGAGTACAAAAAGCTTAGCA
ATTTGCAATATAAGTAGTACCTTTCCCAAGTTTGAAAGTGGGTTATATTATTGCTGGC
ATTTATTAAGCAAGGTGATTAAGTTTTATTAGTAGGGGATTGGATAGAATTCATCTTA
TTATTGTACTTGAGCTTGATAACTTTGAGAAAATAAAAGTTGCAGAAAATGATTGGTA
CTGTTCATTGCATTCTATTGACTTTGGTTTTGTGTTCTTATATTGTATTTACTTTTGTTTTT
GGGTTGTAAAAATGTAAACTAATATTTGTTGGTTTTACACAAGAATAGGTTCCTTTAA
GTTGTTTCCGAGTGCTACGATGCATCCGAGTTTCTGTATGAATTGAGGCGACGACCAT
GAGGGATTTGTGCTGCACAACTGCTGATTGTACTCTGCCTCTTTTTCTTTTCAATTAAA
GGTATGACTTATTGTTGTGCATTGAGGTTTAGTTATGGTGTTGATCCGTCGTGAATTTG
TGATTATAATGGTTAACTCCTGTCTGAATATCAAATGTTTGTTCATTTATTTTGTTTCTT
GTTTCTTAACACCAATCATGTTAGTTGTTTTTAAGGCCTGCTTCAAAAAATTTGAAGAT
CTTGCTGTTATATTGTTTTTTGCAGTTTCAATCAGTATTCGATGATATTCAGCCATTCAG
GTTAATTAGTGGAGAGATCTAGTCTGTCCTGAAGCTTCAGGGTTATTCTGCTTTCAAA
GATTTTTTTTTG

>*CqSPL2*
GTCGAGGCTTCTCCACCAGGCGCCGTTACCACGGTGGCGGCGTCCTGGCTAGGCGCC
ACAACCACGGCATGGCCTTTTGGCTAGGCGCCAGCCACCATTTCGTCCTTACGATGCG
TTACGAACCTCGTTTTCCGATGACGACGGCGATTCTCCGGTGATCCTCGGCGATCCGG
TGCTGCTCCACATTTTTTTAGTGCGAAAAATTTATCTTCGGTTAAAATTATAAAAAGG
GCAAATTTGTCAAAAAATTATAAAATGTGGGCTTTATGTAGAGATTTGCGGGCGATG
AATAACAAGTCCCAAAAAAAAAAGGGGAGGGGTCTTAATTAACACCCCTTATGTAGTA
ATTTTATACATATACTATATTACTCATAACTTCATAACACTTATATCTGGTATCTTATTA
AATGAGGGTTTATCGTCTTTTACTTGCCCTATTCACTTCACATAAAAAAAAAAATTAAA
AAAATAAAATAAAATAAAATAAAATTGTAATATTTAGTAAAATTAATTTCAATTCAA
AATAATTAATTTCAGTTTATTAATAATAAGTTCAATTCATTAAAAATTATTTCATTTCA
ATAAAAATAAGTAACTTTAAAGAGTAAATACAGCTTAGCAAAAATAATTTCAATTCA
TTAAAATTAATTTTAATTCAGTAAAATCAATTTAAGTTCGGCAAAAATAATTTCACTG
AGGATAAAGTAAGTAGGGTCTAAAAATAAAAAATAAAAAAAACTTATTTCTAGTAA
AATTATAGTGATAATGGCTTCTTAAAAAAAAATAAAAAATCCCACACATTTTGTATG
AATATTATTAGCTGTCCTTACATAATCCCTCCAACCAATAATTCTCCTTCACCTGTCTT
TCTCTCTCTTCACTCCCTTTCTCTCTCACCCTTTCATGGCGTCTTATTGAAATTGGTGAA
AAGTGGAACACTAATTATTAAATAAACCCAGATTTTTTTTTTTTTAGAAAAAGGGTA
AGCTTCCTTTGAGCTTTTCTTACCTAAAATTGATGAGATTTCTAGATAGAGAAAGAAA
AGATTATCATTGTTGGCTAAAAGGTATGAACTTCCATATTTATTTTATACTAAATTTGG
GTTCTCCTTCTCTTTTACTAAATCAAACTTATAAAATATTATTTGATTCTGATTCAGTAC
TATTTTAAGTAAATATTTATGAGTAATTTCTGGGTTATTTTTATTATTCTCCTGTGTTTAT
TCTGCTAGTTCGTCTTTTTTTCAGTGCAGATTGATAGATTGACATAGTTACTTTTTTAGG
CTAAACATAATTTTATGATTTTATTTCTAGTACTGGGTATTTTTTGCAGTTATGGTTATG
TTTTTCCCAGAAAAAAAGTTTTAATTTTTGATTCATTGATTTCTGTGATTTGGGTTTTTT
ATTTTTTACTTGTATTTTTCTGGGTTAGTGAACATGAAAACGGTGTCGTTTCGCTGCTTC
TTTTTATTTTATTTATAATTTCGAGCTAATAATTTATTTTAAAAAAAAAAAAAAAAACAT
TTAAGCCCTTCAAGTAGAGCAAGAGGCAAAAACCCAGAATTTGGGTTTTTGTAAATC

TTTTATATTGACGATGATTTGCGTGAACGTGTGATGGGTTTTATTCTCTCCAAAAGCT
GGGAACTTGACTTTTTGACTAGTATTTTTAAGAAATAAAATTAATAAAATGTTGGGA
TTATGGGAAAGATCATACTCACATTGTAATATGTTCATTATTATAATTTCTATTAGTAT
ATACTTGTAAAAAAATATAAAATAAATAGGAATCCACTTTGTTCATATTTTAAATCTG
TCAATTGGGTTTTTCAAGAAAGGGTAAATTGTTTGATTAATATTTTGATGGGTCATTAA
GCTTTACAAATCAATTAGGGTTTTTGGGAAGATGGGGTCATTTTTAGATCTGCAATGA
TTTGGTGGGCATCCTGTATTAAAAGGGGTTTTTGGGTTAAAATTGTATCAGTTGATAA
AAATATAGTGTTACGTTTCACTCTAATAGACCACATTCTGCTCGCTTTCGTTTTCGAGA
GTAGTAAAAGTCGAGTAAAGATAAAAACTATCAATTCATTATAGGGTGACTAGTTGC
CAGTCTTGCCACAAATAATTGGAGTGAGAAAAAGAGAAGGTTATGGGTGGTTGGATG
GTCTGAAAAAATGAAACGAGCATGGCAACTCTATTCTATAAAATGAGGTTAAATTAA
TTACTTTCCAAATAGGTTAATTGGGGGGATGGTAAAATTTCTATAAATGGGGTTATAA
GAGTACAAAAAGCTTAGCAATTTGCATTTAAGTAGTACCTTTCCCAAGTTTGAAAGTG
GGTTATATTATTGCTGGCATTTGTGAGCAAGGTGATTAGGTTTTATTAGTAGGGGATTG
GATAAAATTCATCTTATTATTGTACTTCAGCTTAAAAACTTTGAGAAAATAAAAGTTG
CAGAAAATGATTGGTACTGTTCATTGTTATATTGACTTTGATTTTGTGTTCTTATATTTA
CTTTTGTTTTTGGGTTGTGAAAATGTAAACTAATATTTGTTGGTTTACACAAGAATAGG
TTCCTTTAAATTGTTTCCAAGTGCTACGATGCATCGGAGTTTCTGTATGAATTGAGGCG
ACGACCATGAGGGATTTGTGCTGCACAACTGCTGATTGTACTCTGCCTCTTTTTCTTCT
CAATTAAAGGTATGACTTATTGTTATATAGTGAGGTTAAGTTATGGTGTTGATCTGTCG
TGAATTTGTGATTATAATAATTAACTCCTGTCTGAATATCGAATGTTTGATCATTTATTT
TGTTTCTTGTTTCTTAACACCAATCACTATAGTTGTTTTTAAGGCCTGCTTCAAAAAATT
TGAAGATCTTGCTGTTCTATTGTTTTTGCAGCTTCAATCAGTATTCGATGATATTCAGC
CATTCAGGTTAATTAGTGGAGAGATCTAGTCTGTTCTGAAGCTTCAGGGTTATTCTGCT
TTCAAAGATTTTTTTG

>*CqSPL3*
TATATATGTCGTTTCCTATTTTTTATTTTACATAAAATGAAGACAAACGAGCCCTTAGA
TATTTGTTTGGTTTAATTTGGACCTTTACGTTCCAAGTTGCAATAAGAGAGTTTGATTT
ATGTTTTGAATCATAATTTGAATTTAATTTAGTTTGAAAACTACTTGCCTAATGTGATA
GAAACTAAGTGTTCGAGGACCAACTCGATCAGCAAATGTTTAAGTTCATAGTTGAAG
TCCAAGATTGGTTTTTATTCTCTATCACGCTCCCTCACATAAGAATCTATGAGATTTCA
AATGTGGATGCAACATGGGACTCCTCATACCCAGCCCAATGCTAAATATTCCCATGA
AAGGAGGGGTGGTTGAAATTAAAACCCATGACCTATTTGTCATAAAGGTTCTATAAT
ATACCATGATAGAGGTCCACTCAACCAAAAGCTTAATTTTATGGTTGAAGCCCAAGA
TCGGTTTTAATCACTAAGATTGACTTTTATTAGGATTGTGAATGCTATTCCGCATAGGG
ACTAGATTCATAAAAGCAGGCAAACTTAGGAAGCGAATGGGAGAGAGTTTTTGTAGG
CACCTTGTTGTGGCTATGGAGAAGGCAAAACAAGAGGTGTTCTTATGCTAATCTTAAT
AATCCAACTAACCAAGAAAGCTTGCTCTAGTGGTAACAATCAAAATAGCCATGAAA
AGTGAGATTTTAATATACTGCATAATAGATCACAAAGAAAATAAGTACATATTAATT
GGTTGAGCAGTTAGATTTTTTATTTTATTATTTTATTTTATTTTATTTTATTTGTTTTTTT
GGTGAAAGAAAGTTAGTGCTCTTTATTAGTCATGGTTGAGCAGTCAGATTAGGTGAAT
GTGATTATATACAGATGGTGCAGCAAAGGGTAACCTAGGAAGGGCAAGGGCATGAG
GCTTGATTAGAGATAACAGTTGAGAGCTTCATAAGTTGTTTGCCTTGTATTCTGGCATT

TACACTTGTACAAAAGCCGAGCTTTTAACTATTTAATTGGGCAGCTAATCAAGCAGTG
GATTGGTCAGCTAATTATGAACTTAATTCGGAACAAAAACTCGTGTTATATAAAGCG
GTCCTTAAGAATCTTCATGTTGTATTATTAGAGGATCTTAGCGTAGTGGCTTGGCTGAG
ATTGGTGCCTGAGAAGCCAGGACAATTGTTTCTTTTTCTTCCTTTCAGTCGTATTGATTT
TTTTTTTTAGTTTCTTCTAAGCTTGTTATTTCTTTAGGGTTTGCCCCTCTCGTTCAAAAAA
ATAAAAAATCAAATTAGACACATGCCCAAGTATTAGCGATACCCATAGATAATGTTA
GAAGAGAATAAAATTCCATAAATACGCTACATTTTAATAAAATTCCCTGCGTACCGTT
CACGTCATCTTTTGTAAACTTGATTTTTTATAAAAAAAAATAAAAAGCTTGAAAAAAC
CGCTAATTCAATTAGCGGTTTATTTTTGCACCCATTAACTCGAGCAAGCCAATCAATA
TTACAGTACATAAAACCGCTAATTCAATTAGCGGTATTACATTATATGAAACCGTTAA
TTCAGGGTTCGTTTGGTATCGCTGTAGTTTTCTGTTTTTCAAAATCATGTGATATATATC
ATACAATGAATCAAAAATTAGTCAAACTTATCAAAATCATGATAATTTGGAAAACTG
ACAACAGAAAAGTGAAAATTTCTTTTTCTTATTTTCTGTTTTTGGTTTTAAAAAAAACT
AAAAACTGAAAACAAAAACAATGCCAAACAAGCCCTCAATTAGCAATTTTGTTTTAA
ATTTGGCGAATTTTTTTTTTAATAATTACACTTGCTGACGTGGACGGTACATCAGAAAT
TTTATTAAAATGTAGCGTATTTATGGAATTTTGTTCTCTTCTCACATCATCCATGGAAA
TCGCTCCAAGTATTTACAAGAAGTTAATTTTTTTTTTAAAAAAAGGCATAAAAAAGAA
TAACCTAACTAAAATGATGCTGACATACATCCCACAAACTTTTCGTTGCAAAATAGG
GTCCACTCCATTTGAGCAGTACTATGTATCTGGTTGGGAGCAAAATTTGTGTGCAAAA
ATCAATCTTCTTATTTTAGTTGTTTAGACTTCGAAACATATTTTTTGATGAGAGAGAAT
CAATAGCCCATAATTGTGGCCCTGTTTTGGTAGATTCGCCTAACTGCCATAGATTTTAG
ATAGCGGTTTTCATAATACTTTAATCAATTTTTGTACTGCAATACCAAATAGTCCAAG
ATTACTTTTTTAGATGAAGAAAAATTAAATATAATTACAAAAGAAAACCCCAATTTTT
TTTCAAAGAAAAAAAAAATAAAGATAACATTTTTGTACGATACTCGGTTTTTTTTTTGTT
GTTTTCTATAGTGGGTTTTTTTATTTTCATGCCTCCCTTCAATTTTGGTTCAATTTATCTC
GCTTTTTGACAAATTCAACATCGTCCTCCAATATTCCTCTCTCTACATTTTGGGGTTCTC
GTTTCTTTTTTCTTCATAATTTTAATTTTTGTTTACAGATCTTGCGTTTGGTTCGCAAGCC
AAAATCAAGGTTAGATTCAATGTTTTGAGTTACACAGTGTATTTTTTTATTTATATATA
TATTTGCACTTCAACTGTCTGTTGAATTTACTTTAACAAAGAACTGGGTATTGTAAAA
GAGTGATATTTATTGATGTTGTTGTGAAATGTGTTGTGGGTTTTCGATTTAAATTTATTT
GTGAAGGTTTTGATTCGTTGAACTGGTAATTTGGTAGCTTTGTTAGTATAAATTTTAAT
TCTTGCTCATGCATAGAAGCTGGTTTGTTGTTTTTGAGGGGTTTATTGAGCAATTCTTGT
TATATGTTTTCAAGGGAGCTTAGTTCAATTGGTGCATTCTCTATCTGATGGAATTGGGT
AGGGAATCAGGGTTTAAGATTAAAGTTGCATATATAATTGGAATTTGTGCTGTTTAGA
GGGTTATATCTGTAAACA

>CqSPL4

CAAACCCTCAAACAGTTCTACCTTTCATGCCTTGGTTTAAGGACAGAACCTCGGGTAA
AGTACGGACTCCTTTGAGTGCTTAAGGAAGGTGTTCTGAAAGAATAAGATAGGCTAG
TGTTTAGGATAGAGCATAGAAGAGAGAAGAGAACGTTAATTCATGCCTCGAACAAC
ATAAATGAGGGGGTATTTATATGGAAAATGAGCAGAAGGAACACACGGAACCCTGA
AAAAGATAAAAACCCTAAAAAATAATGACACGACACGTCAAAGCATACCACGACAC
GACAAAGTATGCCGTGACACGGTACACTTTAGTGTGACGTGGGGAAAACCGATGTGA
CGCGGGAAAGAATTAACCTATAGAGGATAAAAGCACGTGGAGGATTTAAGCGGAGA

AAACACGGAATGGCCTCGTGTCACGACAGGGGCATCGTGACACAAGAAAATACCCC
GTGACGCGGCAATATTTCCCGTGGCGCGGCACCTGTTTCTGTGACACGATGGAATTTA
TTTCCATTTTTTGCGTCCATATTCACTTTTACCCTAATTATGCTCGGCATTTTTAAGTGT
AGTTTCATAATTTCGGGCTATTTCAGTTTATTATTTAATAATTGGGCTAAATAATAAAT
AATATAAAATGAATAAAAAGGTTTTGGCCAAAATTGGACTATGCTTTGACTAAACAT
TAGGACTGATTTTATGAACTGAGTTTGATTGAATATGAATTTTAAGCACTCCCGGGAT
TAAATGTTAAACCAGACTAATAATGGCAGTGAAAGGTTGTTCATCACCGGGTTTGGTT
TCGGGGAGACATTTTTAGGTGTCTACAATAACATATTGGGATTGAGAGATGAAAGTT
ATTTGTACATTATTTGATTTTTTGGTTTATTGAATTTAGTTAGGGCTCGTTCAACATCCC
TATGGTTTTTAATTATTGATTTTTATTTATTTTATTTTATTATAAATTTTGAAATCATATG
ATATACCCTTATCTCTTAAAACGCTCAAACAAACCCTAATAAATATTGTGCGAACATT
TATACCCTTAAAAATAATGTTTGTGTACTCATCGGCCTTCATTAATGTTAACTGTCTCT
TTTGTACCCCTAATAAATAAATAGTGTACGGACATTTATATCCATATAAATATTTGTTT
ACATATTCTTTCTGATTAATGCTGCTAACAACTAAATTTATGACATAAAATCAACTAA
ATTAGTCTAATTTATTATAATTATCATATATTTGAAAACTAACAACAAAAAAGTCCAA
ACTAGTTTATGTCGTTTAGATATTTGTTTGGTTTAATTTGGACCATTAGGTTTCAAGATA
CAATAAGAGGGTTTGATTTGTTTTGAATCGGAATGTGAATTTATTTGACTTAGAAAAC
AGCTCACTTAATGTGATATAAATTTATACTAAGTGTCAGGGGACCAACTCGATAAAA
GTTTAAGTTCAGAGTTGAAGTCCAAGATTGATTTTTATTCTCTATCACGCTCCCTTACA
TAAGAACTTATTGAGTTTCAAATGTGGATGTAGCATCGGCCTCCACATATCCAACACT
AAATATTAAGCGGTTGATCTCTCTGATTGGATGAATGCGGTTAAATACAAATGGTGCA
TCAAAGGGTAACCTAGAAGGGCAAGTGGCTTGATTTGAAATAGCGGTAGAGAGCTTC
ATAAGATGTTACCCTTGTATTGTGCCTTTTATTAAGTATACAAAAGTTAAGCTTTTAAC
TATTATTATTAGGTAGCAAATTAAGCAGTGGATTGATTAGCCGATTATGAGGTTAATT
TGAAAGAAAAACTAGTGTCTGATTATTTTGTTTTCTTAGATTCTCCTAAGCCGTTTATT
TTCTTCAAGGTTCACATTTTTTTTTAGAAAAAAAAAAAAAAAATCAAATTAGACACATGC
TCAGCCCAAGTATTACAAGAAGTTAATTTTTTTGAAAAAAGAATAAAAAAGAAGAAT
AACCTAACTAAAATGATGCTGACATACATCCCACAAACTTTTCGTTGCAAAATAGGG
TCCACAGTCCACTCCATTTGAGCAGTACTATGTATCTGGTTGGGAGCAACATTTGTGT
GCAAAAATCAATCTTCTTATTTTAGTTGTTTAGAATTCCAAACAATTTTTTTGATGAGA
GAGAATCAATTGCCCATAATTGTGGCCCTGTTTTGGTAGATTCGCCTAACTTCCATAG
ATTTAGATTGTGGTTTTCATATTACTTTAATCAATTTTTTGTACTGCAATACCCAATAGT
CCAAGATTACTTTTTTTTTTTTTTTTTAATGAAGAAAAATTAAATTAAATATAATTACA
AAAGAAAACCCCTATTTTAAAAAAATAAAAATAAAGATAACATTTTTGTACGATTAC
TCGGTTTTTTTTTGTTGTTTTCTAAAGTGGGTTTTTTCATGTCTCTCTTCAATTTGGGTTC
AATTTATCTCACTTTTTGACAAATTCAACATCGTCCTCCAATACCTCTCTCTACATTTTG
GGGTTTTGGGTGTTTTTTTTTTTTTTAGATCTTAATTTTTGTTTTCAGATCTTGCGGTTGGT
TCGCAAGCCAGAATCAAGGTTAGATTCAATGTTTTGAGTTATACAGTGTATTTTTTTTT
TATGTATATAGATTTGCACTTCAACTGTTTGTTGAATTTACTCAAAGAAAGAACTGGG
TATTGTAAAAGATGTGATTTTTATTGATGTTGTTGTGAATTGTATTGTGGGTTTTCGATT
CAAGTTTTTCTGTGAAGGTATTGATTCGTTGAACTGGTATATTGGTAGCTTTGTTAGTA
TAAATTTTGATTCTTGCTCATGAATAGAAGCTGGTTAGTTGTTTTTGAGGGGTTTATTG
AGAATTTCTTGTATGTTTTCAAGGGAGCTTAGTTCAATCGGTGCATCCTCTATCTGATG

GAATTGGGTAGGGAAATCTGGGTTTAAGATTTAAGTTGCATATATAATTGGAAATTGA
GCTGTTTTGAGGGTTATATCTGTAAACA

>*CqSPL5*
AGAAGGGCAAAATCGTATTTTCCTTAAAAACACTTCTTTTATTCTGATTAAAACTCTA
AAAAGTCTACCAAAGTCAATTTATCAATGCTAATTGAATTAGGAATGAAAAGAATCT
TCTAGAAGACTAAAATCAATTCTAATTGCATTAGGAATGATAAATCAATATTAAGGC
CTCCTCTAGATCATTGGGCCGCACATAAATATCAAACTGGGCCCAGTTAGTAATTTGG
CCATAACTTCCTCATTATTCATCGGAATTGGGCTTATAATATGTCGTTGGAATTAGGGC
TGTTCAATTTCGGATATCCGATCTGATCCGAAAATTCGGATATCCGAAATTTTCGGAT
CGGAGAAATTGCGATCCGAATCCGATCCGATTGGATATTCGAATATTCGTATCGAAA
GTCGGATATTCGGATCGGATAGTCGGGTTTTCGGATCGGGTAGTTAAAATTTTCGGAC
AAAATCCGATCCGTATCCGATCCGAAAATCCGAATTTTTTTCGGATATCCGATCCGAA
ATATCCGAAACCTTAAAAACCGGATCGGATAATCGAAATTTTGGATCGGATATTTCG
GACCGGATATTTTTGAACAACCCTAGTTGGAAAGGTCTTTTCGAACCCAACCCAACTA
CACTATTATTGGACCCTTGCTATGGACCAAACCAAACTGGATTGGCTGTTTTCTTAAA
ATAGGTTTTCAAAAACAGAGAGAAAGAAAACTAACCGCAACACTAGAATTTCCACA
CTTTCACTTCATTCGATTATGATTTGACGTGAAAGGTACTCATCACCGGGTTTAATTTG
GTTTTGGGGAGACACATTTGGGTGTCTACAATAACCCAATCATCTCTGTCCTCTTCATG
TGCCATAGTAGCTTGCCAAATCTTAGAGTGACCATGCACTTGCAGGCCAACGCCTTTG
GAGCCATCATATAAAATGTTGTTGAATTCATCAGGATTACGCTGTAAATGGAATTGCA
CTAACAAAACACCAGGCCAACCCAACATATTAAATTAGCAAGGAGCAAACAGAAGC
ATGCAGAAATTAGACAAACCAAATCACATACATATAGCTATATCTCTTACATGCATA
ACAACAACAACAACTTAAAAATGTATATTTCATACCAACTTATCTTCCTGATTTCCTTC
ATAACAAATTGTAAACGTAGGTGCAAGATTAGCCATCCAAAATGCCTAAACATATTA
CTAATGTATATGCTTATACTTAGCTTATAATCATAATTCAGTGGCTGATTTGCATATGA
TAACAAACAATGCTATTTATTTATAGAACTAAAACATTGCTAGAAATGTATATCACAT
TAAATGCAATTAAATATAGCTTTGGCAAGAAAAGGCTTAAGCAATATTCTGTTTTCAG
AAACTGAAACTGAACTCAAATTCAAGATTCTTTCCCAAGAGTCATACTAGCTATAAT
GACAAGTCAAGTATGTTCAAAAGAGGTATAATAGTCTATATGAATACAACATTATAT
TAACTTACATTCCTCTTGATACATTACAAATAACTTACAAATGCAACTTATTTATCTCC
AATATGCTATGCAAAACTACATGTGTAATGCTCACTGGTAATTGCTAGCTAATTTAGA
TCGAGTCATCTTTCTCGGAACTGAATTGACTATCCAAGCTATGTATTAGTCAATCTCTA
CTATTACTTAACTAATGAACTTCATCCATTCATGGTCATTATTGACATTCTATACAAAT
GCTTAAGTAATACATTAATGTCTATGTATAACATTCCTTCTTAGACAACATAATAGAT
AGCTACTTATAAATTGTTACTCTATATTCAATGAGATTCAAAATAATAAGTAAATCAA
ATCATATAGACATATAAAACATGCAATCCTTTTTTTGACAGTTACAACAACACAAACT
TTTAAGATACATGTAAGTCAACAATACGTATTGAAAACTATGAAATAATGGTTATCTA
AATTGTTGACTTACTTGAACACATGTGATTACAATCCACGTGTGCAAAGGAAGCAAT
GTCAAACATATTTCAATTTTAAACGATTTGTTTTACAAAATGTATCTTGTATATTCAAA
CACAAAATAATACGAAGTAATAGGTAATCCAAATATATTTATTTAGTGTTTCCTTGTT
AGCGCCTTAATTTTAATTAGCAAAATGGTGAGCGCGGAGCACTAAACTAGTAATAAT
ATAAAAAGGCAAACAAGACGACGGATTGTACAAATGCTTGAAAACCAAATAAACTC
CAACTCAAACTTGCAGTCTGTATTGTAAAAATCTCCCATGTTTAGTTGTTTACTACAAT

ACCAACTCATGTGTTCTATACTTCCCAGTTTCCATATCATTGATAGCAACAATAATTAT
TCACTGAAATTTACGACAATACCCCAGGTGCACCTAAAGGAAGGTACACAACACTAC
AACAGGAGCTGAATTTATCTCACTTTACAAATTCAACATCGTCCTCCAATATTCCTCTC
TCTACATTTTGGGGTTTTCGTTTATTTGTTTTTTTAATTTTAATTTTTGCTTTCAGATCTTG
CGTTTGGTTCGCAAGCCAGAATCAAGGTTAGATTCAATGTTTTGAGTTAAAGAGTGTA
TTTTTTTTATGTATGTATATTTGCACTTCAACTGTTTGTTGAATTTACTCACAGAAAGAA
CTGGGTATTGTAAAAGATGTGATTTTTATTGATGTTTTTGTGAAATGTGTTGTGGGTTTT
CGATTTAAGTTTATTTGTGAAGGATTTGATTCGTTGAACTGGTAATTTGGTAGCTTTGT
TAGTATAAATTTTAATTCTTGCTCGTGCATAGAAGCTGGTTAGTTGTTTTTGAGGGGCT
TATTGAGCAATTCTTGTTATATGTTTTCAAGGGAGCTTAGTTTAATTGGTGCATTCTCT
ATCTGATGGAATTGGGTAGGTAAATCAGGGTTTAAGATTAAAGTTGAACATATAATT
GGAAATTGTGCTGTTTAGAGGGTTTAATTTGTAAACA

>*CqSPL6*
CCAATTGTGAAACTTGGAAGGTTCAATGATACAAATATTCACTCAGTCACTACATGTT
TTTAATGAAAGGTAGGAAATAAGGAATCTATGTTGGAATCTTGGCCTTATTGACTCAG
ATAAATTGAATGGATACACCAAAGAGTTGTCATTTTCTATTGGATAAGAAGAGTACA
AATGCATATGACATACTTATATATATTGTGCTTAAATTAATCAATTTAAAATGAAGTTT
AGTAATCATTTGCGCCAAATAAAACAGAATAATACATTTTTTTGAAGAAAAAAACAG
AATAATACATAACTGAAGGTATCAAAAGTAAAATTAATTAATATAAACAGTTAAAGT
AGGAAGAACAAATAAAAAAGTAAAAAAAAAAAAACAAAGGGAACATTATTCAATTC
AGTTAAGTTCAACTTTAGTTAGTTTAGTTCAATTCTATTATGTTTATATTAACTCAACTT
ATAATTCACTTTAAAAGAACATGCCTTAACTCATTTAATTTAAACGAATTTGCATCGA
TTTTCTAAAAGTAAAGTATGCTAATGAGTTTACATGAATAGTTACTACCAAACAATAT
TGTCCTTTAAGGGGAACCATGAACATCACTCCCGAATCTAATGCTTCTAAGATTCTAA
GATATAATTCTTTATCTTCACCTGACTTCCCCAACCCCCCCCCCCTCCCCCCCCCCCTC
CTTCTTTTTTGATCTTGGACAAATTAAATGTTCCTAACATAAATAAGTTTATATATTTC
ATTTTTTCTCTTTATTTATCCCATTGAATATTGTTAGAAAAATAATCTAGTATGATCAA
GTAGGACTAGTTGTTTCACATTGAAGAAATGAAGGAAGAATGACGGAGTAACATGTA
AGATTGGTGGGCGGAGGTTCTTTCCTTTTTTCATCAATGAGAAAGAATAATTGATTTTA
GGATGGAACTCTGTTAGGTTTATATGTGATCATATTATACTCTAGCGTTGGCTTGTGCT
AGCTTAGGTTGACTTTATGAAGATTTATCATGGTATCTGAACGAACGATCCTGACCCA
TTATCAACACCTAATAAGGAAGGATTTAGCACTTTGAAGAATACAAGATGTCAGATC
AAACAATCTCAGCCCATTATTAGCACCCATCAAGGAAGGGCCCAATGATTTGAACAA
TGCAAGATGAAAAGGATTAAAAGATTGAGAAGAATCTTTGATAAATATATATGAAA
AAAAAAACACATTTGTATGAGATTTTGTTATGGTAATACAAAGGATTTAACAAAAGC
ACCTTCTTGAATTTTGTGGACATATACACATGATGCAATAGTTTAATTCATTGTGTTGT
TCTTATTGTAGCTAAAAGCATGGGCTAAGCCACAACATGTATACACTTATGAGTGAC
AAAAAAAAAAAATAGGAATATGATAAACAAGGAAGCACAACGACGTACTGTCAGTTA
ATGAGTAATAACGTGAGTAAAAATGTTGTTGACAAGTAAAGGAACAAATCTGCATGC
TCTAAATGCTTCCAATTCATGGAATAGGACTCAAAGATGTAATCTTTTAAGTGCTATA
AGTGGTCCGAATTATGTATAAATGTGACCTTATTGTATGAATGTATGTATGTCTTTCAT
CACTCCTCTAATCCTCTTTAGACACTAGATGCCCGGACCCGCTCCTTTTGCTTAGATAT
GAAAAAGACATGATTTGAGCAAGATGATTAATTCTGCTTTCGAGAATTTGTGAATTGT

GATTCTTGTATGACACGGTTGTACGATAAGGATTTTTTTTTTATTTTTTTTTTAATGGGT
GACTTTGAGATTAAGATGTAAATATTGGGTGACATTTTTCATCTCGTGCTAGATTTTAT
TTTTAGGCTTTTTTTTTTTTGAAAATTAAAACGTTCTCTTAGACATATGGTTCAAATTAT
CAAAATGTAACAACACTTTATTTTAGTTCAAATTTATTTTTTTGGACTTTAGAGGGTTG
GAATTTCTTCTTTTTACTTTTGGATGGTTGTAATCCATGAGTGTTAACCTATAAAACTA
ATGACATTTAGTATTTCTTTGTTAGGGATTAGGAGTATAAGACTTCGCATCAAATGGT
GCTAGAAGTTGCAATATTGTTTAAAGTCGTGCATGTATCACATCAAAACAACCCCCTA
ATATAATAGACCCAACAATGAAATTTGATGCACACATTATACCCTAAGCTTATTGCGT
TATTGGGTGAGTTTTTTTCTATCTCCTAATTCCGATTTGATTCTCTCTGATCTCTTCACA
CGCAAAATCAATATCTCCAATAATAAATGTTTTTTAAGGTACACTTTCATTACATCTTC
CCCATTATTAAAAAAGGCTTTAGTTAATGATCTATAAGAGGTGGTACAAGATACACA
TGTATTTAGGACTATATTAATAATGACTTTAGAAGTAATAAAAATAATTTCTTTTGAG
ATTTTTTTTTCGATGGAAGTTGACTTAGTTTAGTCAAGATTACATTATTAGTAAGCTTT
CATTGCCCTTTTCTTTTGGGAAATTTTCAATGGTGACCCCCAACTTGTACTACGCACAC
ATGGTGACCCTCTTGAAAATAGATTATACAAAATGACCTATTTAAAAATTCTATCTCA
CTTATAACCTTATTTCATCAAATTGTAAATAAAAATTAACTATGGTTAGATTTTTGTA
AAAAAAAAAAAGAAAAAAAAAAAATTACAACCACCAACTATCCACCCACACCACCA
GCCAGCCACCATCTCCCTCTCCCACTTCACTACTAGCACCCTAGGTTCCCCCCCTCGTC
GGCGACGCTATCCCAGCCCCACCTCGACCACCGCCCACCATGAACCCCAAACCCTGT
CCACGAGGCCACTCTTCTCCACCCACCGCAACCCCATCTTTTTCTCCTCTGCCCAGATC
GAACCCAAACCGTCTCAGTTAGGGTTTCGACTAAGGTGGGGTGGTTGGGGTTAGGGT
TTCGGAACCACCTTACCTCAGTCGAAACCCTAAATGG

>*CqSPL7*

AAGATGCTCCCGTTCCCTCCTTTCGGTATGACGGGTACGTCGGGCCCTTAGGGTCCCG
TCTGGAGCTGTATGAGTTTCCACTTCAAATTCACTTTCAGTGTCATGGTCCGACTGAAC
TTGAGGGTCGTTCAGGGGACGCCTCTCTTCAGGAGTCGGTACAGACTTGGATGCCATC
TCAGAAGTACTCTCCCCACAGACGGCGCCAAATGTTTCGGGGTTTTCTGATGTCCTGC
GGGAGAGTTACAACACCGAGACTGGAACCGGACGCCTGTACCTGCAAAATAACGGA
GACTCCTGAGAGCTCCGCCTCCAACGGTCAAGTTAGTACCTGATTAAGGAGTAATTG
ATACGGAAATTAAAGTGGTTTAACCTCTCTAGGTGAGAGTGTTTAATGTATAATCCGT
GTGTTATTCGAGTTCTTTTTTCCTTAATGAGGATTAAGAGGAGTATTTATAGTCTTGAG
AAGCGTGCCAGTCTCTAGGTTTAGGGTAAACCTAGGGCTGACCGGGCCTCTAAACAG
GATTGGGCCTTGGTGGCCGGTTTTCCCATTTGGCATGGGTGGACACGTGGCGTGTCTC
AATTGGTCCGCAGAGGACGAATCTGTGATTGACACGTGGCAAGCTCCTATTGGCCTA
CGTGAAGGACACGTGGCGGAACCTGGTTGGTCCACTTGTCAGTCGGTGATTGGAGGA
TAAAAATCCCCTAATCAAGAGTTATACAGTAAACCACTAAATTACAATTATACAATT
ATATACTCATTAAATCAACTACAACTTTAATTCTTCTACTTCATAAATCAGATTAAATC
AGTTGCAAGAAGTACGAGGAAGAGAGATATCATACCTGGACAAAAAAAGAGAGGTT
TAGAGCAGCAACAACACTGATAAGCGAGGATTAGAGCAGCAACAACACCGATGAGC
AGCAACAACGAACGAAAGGCGCCGTGAATGAATTTGGGAGAATTGAAAATGAATGA
GAGAGACGACAAACGAAGAGGCGCCGTGAATGAATGAGTGAGATCTGAGAGGAGA
TGAGAGAGAGAAAGAACTAGGAGGCGTGAAAGTGAAAGTGGAATTAGGGTATCAA
AATGGTTGAGATGTAAATCTAACCATTGATTTTTTAATCTATGGTTGAGAATGGTGAT

CCGTGTGTGTTGTGGGGAAAAAAAGCTAACCGTAGATTGTTTTAATCTATGGTTGAG
AGTGGTGATCCGTGTGTGTTGTGGGAGAAAAAAATTAACCGTAGATTGTTTTTGATCT
ATGGTTGAGACTGATTAGATGATGTTTTGTGTGTTGTGTATTCATTTGATCGGGATCAG
TCTCCAAGACGCGTATTAAAACATCTACGTAGTATATCTTTATTTTCATACAAGTAAA
AATTATAAAAACGTTATATTAATAAAATATATAATGATACAAATTTAAAAAGATCAC
TCATGAAGTCATGAGTCATGACTCATGAGTCTCGTGACAAAATTAAGTTTTACTGTTTT
ATAGATTAGTGGTAATTTATGGTCAATATTTTCAAAGTTTGACCCCAAAAATGACAAA
GGGACAAAATAAAGAGACGGATGGAATATTAAAATAATAGTTAGAGATGGTGTAGA
TCATTATACTTAAACTACACGAACAATGATCCTAATTTAATTTAAAACAACAGCTCAA
GTTATTGATCCAATCAAAATAATGTTCACTAATTTATAACTTTGTACTAATACATACG
AAGTATTTAACTATGAGATGGTTAGATGCTATGCGTTAATGTGTACATACGATCGATG
GAACTAATCAATATTACCTTGTTAATAAATCATTAATGTTTTCTTTCATTACACAAATC
AAACTACTTTGTAATTTCTTCATAATTTCATGAAAATATATAAAGATAAACGAGCCAT
AATTATCAGTCTTGTTGATTAATCAAGTATCGAATTACCAAATTGCAAATTTCCATTTA
ATAAGAACTCATTAAATCATGACTATGACACTAGTATTTAAATGATTAAATAAGAAA
CATATAACAACCTAGTTGTAAAACAATAATACATTAATTCACTAACTTTTATAATCTA
CTTCGTAATACACTAGTAAGATCAATATGACTAAACTTGAAACAGAAGATGATATAT
ATTTATAAAATCAATGTGAATTTGTTTATAATATGATGCCTAAATATGAAATGATGAA
GTCTTTACTTTATATAAAACTTGTTAACCAGCAAATACTATCAAATTTCAAACCAAAA
CCATTTAACTGCCCAAATCCAAAGGACTCGATAAACAATTGATTTTTTGAAACCGAA
AACCGACCCGAAGCGTAATTTAGTAAACTCGGAATGGAACCGATCCGAATAACGTTA
AACCGCAGAAGAACCGAACTAGTTATAAATTGAACCAAAAAAGAACCGGATCAGTT
ATAAACTGAACCGAAAAAAACCGAACCAGTTATAAACCGAACCAAAAAAGAACCG
AACTGTTTCCAACCCGAAACCGCTTCCAACCCGAAACTGTTTGAACCGGAATAAAAA
CTGTTTACACCGAACCATTAACAAACCGCAACCCGTTTGCATCGAACCGTTTACTACC
CGGACTGTTTACAACCCGAACCGAATTAAAACCGATCAAGATTAAACCGAAACCGA
ATCGAACCGATAAATATTTAAACCGAACTGAAACCGACCCGCGAGACACTTAAACC
GATTTAATACCGACCCGATTTAATACCGAACCGATTTCAAACCGAACCAAACCCGAA
CTGTCAAACCGTTTGACAGTGCTTGATGGCCCATAACAAGTCGCTCTTGTCTGCTATCT
TTCCAATTCCCCACTAAAAAGAAACCAACACCACTCTTTTTAAAATCCCCCAAAAAC
CCAGTAAAAAAAATTCATTCATTCATTCATTCATTCATCCCATTTTCTCTCTCTAAAAA
ATATACCCACTTTCTCTCTCCTCAAAATCATGAAGAATTGGGCATAAACAACCATACT
CTCACTCACTCTC

>*CqSPL8A*

AACTCATAAAATCATAAAACAAACATAAGGTATGAGTTTAAACTATTCTAAACATAT
ACCTTATACCTTAATTATGCAAACACCCCTAATAGGATTTATATTGGACGAAAGTTTT
ATACATAGTATTTATTTACATAAACATTACTAAATAAATATTTTTTATCATTTTTTATGA
AGATAAACTTGTATTATTTCCTCCATATATTGTTACTCCGTATTATTTATTAAAATATA
ATTATAATGGTTTTATATTGTTTGTTTCTTTTTGATGATTCATTCATAAAGTCTTTGTGCC
AAAATATATGTGGGACCCATTTTATAAGACTTTTCCAGTTCCAAATCTCTGCAACCAA
AGATTATGGTGCATCACCTTTGCGATTCTCTCTTCCTTTTACTTCTATTACATGACAAAT
GCTCTTCATCTCTATCTCTCCTCGTTTATTCTCACTTGTTATTATCCCTCAAAAAATCAA
ACAAAAAAAAACAATATTATTAATTTTAGGCAACTCATCTTGTGATTTTGTTATAACA

AGCAGTTTGAGTATTACCATCCACAATATTTTTAACAACTTATTCTCAAAGGTTGATCT
GTATAATTAGGCGGACTGGTCAGTATGAGGTCAATACTAAGCTAATTTGAAAGTATG
AATTTGAAGCTAAGGTAGGACTTGTTCTCATTGAATGAACTCAGCTGAACTGAAGTG
AAATGAATATTTTATAAGAGAAGAAAATTAGGAGCTGAACTGAACTGAACTGAAAA
TAAACGTATGAGAATAGATCCGTAGTCAATCACTTTAAAACGCTTTATTAGAATATTC
TCATCATAAGAAGTACTCCGTAATAAATTGTGAATCTTTGTATTACCCCGTATTATAAT
TTGCATATAATTGCATATAAATCGTACAACTATATAGATAATATAGAATTCAGAGAA
AATAGCATCTTAAATTTGATTTGTGAAAGTAGGAGATAGGATTAAAAAGAAAAGAA
AAAAGGAGCATGAAAATCATATCAATAAGGACGCGTTTATCTCAAAGACTAAAATTT
CCACAAATTGTTGTAAAATTGGGACACTTTATCATATGAATCTATGATAAGGTTGTTG
CTTTTGTAGAGATTGGACTTAATTAATTAAGTCACTAGCTTAAAATTCACTCCATTTCA
AATCATACATTCATACCTGTAAGGTTGCAAATTTGTAATCCATTATTATTAAAACTTA
AATTTAATAAGCTGAATAATTTTCTCTATCAATAAAACTTACAAACTCTCCTCAATTTT
TCCTTTAACAAAATTTGATTTGTAGTCTGGATAGCAATATTGTACGATTACATTTATTT
ATCAAATTATTTATTTATGTACTCCGATCCGTAATAAAACACAGCAATAGCCAATAAG
TAAGAATTTGGAAGTATGCTTATATTCATGTAACAGTACAAATGTACAATGTATAATC
ATGCAATTTGCCAATTTGTCTGTAAATAGTTAAAGGTTTATTAACAAACAGAGAGAA
GTTTCCTTGCACTTGCATCTTCATGACGACAATTAAATTGTTAAATTTAAGAACTTTTG
GGATCATCCCATTCAATTCAATAGTAGTGATTATCCTCTAATCATTGATATGATATGGT
CCCAAAATTATATATCTATTCAACTCTAGGTTTTAGCTTCTTTTGATGAAATCGGGAAA
TAGTAATCAACACTAACCACAAATCGTACATTTCTACGGAGTATACTATTATGATCCG
TTTGATAATTGATATGCTTCTTTTGGTCGGAATATATATACATATTGTTACATTGCTCTA
AAAGGTTCCATCTAACTCTTTATAAATAAGGATACTTGTGCAACGTTTATATTTATTTA
GTAAGTAAATTCTCTCAATACAACATTGAAATTTTTTATATAATGACCAAATAAAATA
TATACGAGAATACGACTATTTTTTTGTACGTATTCAATAATGTTATGTATTTTTTAATG
ATACATCTCATCAATACACTTTGACTTCATAAATGTGTAGCAGTATAGCTTATTATGCC
TTATCCCTTTTGTTAACGAAGAGGGTACATTGTATAATTGTATATGCTTATATAATTAT
TATACTTCTTTGATTGTAAGGTTATTTGTATTTAGACGGTGATTTTTTTAAAAAATATAT
ATAAAAAAAAATTCGAAATGAGGACTTTGATGTAACTTTAAAGACGAAAAGAATATA
AGGTTTTTTTTGGGTGTTACAGCCGGTTCACCTTTTGAGTTAATCTGGATTTGGGGCTC
ATTTCTGGGTGGAGAAAGTATAAGTTTAATTAGATAAAATCATACTCAAAGACGTAT
ATTATATAGAGATCAAAGCAACTCAGATACTATATTGAATCTATCTACTCAATTTCAA
GTGCAGACCGTACGGTTCATTGTTGTATTAATATCCAACATTGTCCTGACCTATCCAAT
TGCCTCAAATCTGATTATTATTTTTTTATCAAGAAAGTCAAGTTAGGTAAACCCTTATC
GAACGATATAAAAAATAAAAATAAAGACAAATTAAATGAGTAATGAGTAGAGTCAA
TAATTGATTAAAAAGATACCTTCCCTTCTCTCAACCCCAAAATTACCTCTCATAAAGT
ACCATACATGAAAGAGAGAGATGCTTATAACTTGCCAATTGCCATTACTCAAGTGAG
AAAGCAAAGTGTGTGCAACTCCATAAAGTATTTTTTTCCATTTCTCTCATCTTTCTTAC
TACTACCATTACTACTTGTATTACTCTTCTATCTTTCTTCATTATTCCATGTTCATTCCCA
ACAACAAACTCCCCATGTTTCCAAACAATAATCCCATCTATTTCATCCCATATTTCTCT
TTCATCATCAACCTTATGCTCACTCTCTTCTGTTCAAACTAGGTTACACTACTAATCAA
CTAATTATTATTATTTTAAAATATCAATTCACCCCGCTCTTGTAGTATTCACTCACACA
ATACTATATTCCTAACAAAAAA

>Cq*SPL8B*
TTGCATATAAATCATACAACTATATAACCGATAATTCAAAACATAGAATTTAGTGAA
AATAACCTCTTAAATTTGATTTTCTGAAAGTACTTGTAGGAGATAAGATAAAGGAGAT
TAAAAAAAGAGAAGAGGAAGGAAAAGGGTAGGTGCATGAACATCACATCAATAAT
AAGGTGTTTATCAAAGACTAAAATTTCCACAAATTGTTGTAAAATTGCCACACTTTAT
ACTATGATAAGGTTGTTTTTGAAGAGATTGGACTTAAGTCACTAGCTTACAATTCACT
CCATTTCAAATCATACATTCATACAAATAGGAGTATTAATTAACTTACCTTTTTAATTA
AAAGAAAAGCTATCAATTAACTATATATAGTTAGATGAACATCCAATCACTTGGATG
TACTTATTGGAGGTAAAGAAAAGTTTAATTTGACATGATAGCAATATTGATATATGAT
TGATAAAGCTGATTCGTGCTAACAAAGTAACAACAATTCCTTCCATTATATATGTAAC
TGTTTAATTTTAACATGCACATGTTATCGTTAGTATATTAGAGTGATATTTTGGTTTTAC
ATTTAAATTTTTACGTTTTTGAATAAATTTAATTTGTAATCCAATAGAATGTACTAGTA
AGTAGAAGAAGAGTTACAGATAATAATAAAGGATACAAGAACAGTTGATATTATGA
AGAAAAAAAAAAACGCATAAATGAAGTGATGTTAAATTGTTAATAAGCTAGATTAATC
TCATACGATAATGTTAATGATATTATGAAGATACGTATTAATGAAGTGATGTAGATAA
GCTAGATTATTCTCCTACGATAATATTAAGTTTGTACACATGAATGGTAAGTGGATGG
AACAAAAAAATAAATAATAAAGAGATAATATGAAAACTTTCAATATATATATTGGGC
CGTCAATTATGTTCTGTTATACATATACGGAGGATGTATAATACTTCAATTCATTTATG
TATGTCATTATATGCATCTCATGATGGATTAGCAATATAATACATTTTAATCAAACGA
ATTCAAAATATCGACCCTAAATTTTTGTAAGCTTCATTGATTTTAAAATTAAACATGCT
CAAAAGCGAAAACATAAGGAGTACAACTATAGGAAGGCACAATTTGAGGTAATAAG
TTCAATCATTTGAAAGTTAATATTGGCTTAGCTTAACGATATTCTTATTGGAAATGGCC
ATCCACAACTCTATCATTATTTGATAAAATTTGGAAATAATTTATAGTTACATGAACTT
ATAGTTATAATTAAGAAAAATGATACGTAAAAAATACACTACACCTCTAATTGATTA
TGTATATATTTGTAAGACCGTAATCTATCAATTGTAATTAAAACTTAAATTTAACAAG
CCGAATAATTTTCTCTATCAATAAAACTTACAAACTCTCCTCAATTTTTCCTTTTAACA
AAATTTAATTTGTAGTCAAGATAGCGATGTAGTACGATTTATCATTATTTATTCATGTA
CTCCGTAATAAAACACAGCAATAAGTAAGAATTTGGAAGTATGCTCATATTCATGTA
ACAGTACAAATGTACAATGTATAATCATGCAATTTGTCTGTAAATAATTAAAGGTTTA
TTAACAAACGGAGAGAAGTTTCCTTGCACTTGCATCTTCATGATGACAATTGAATTGT
TAAATTTAAGAACTTTTAGGATCATCCCATTCAATTCACTAGTGATTATCCTCTAATCA
TTGATATGATATGGTCCCTAAATCAAATCAATTCAACTCTAGGTTTTGGCTTATTTTGA
TGAGGAGATCGGGAATTAGTAATCAACACTAACCACAATTGTACATTACTGCAGAGT
ATACTATTATGATCCGTTCCATGTGCTTCTTTTGGCCGGAATATATATATTGTTACATT
GCTCTAAAAGGTTCGATCTAACTCTTTATAAATAAGGATACTTGTGCAACGTTGTATA
TATTTAGTAAGTAAATTCTCTCTATACATTGTAATGTTGTATATAATTTTGACCAACCA
ATAAAATATATGACTATATGAAAGTGGATGAGTATTTTTTTGTACGTATCTAATAATG
TTATGTATTTTTTAATGATATCTCATCAATATACTTTGACTTCTTAAATGTATAGGTTAT
GCCTTATGCCTTTTATAAACGAAGGTTACAATGTATAACCGTATATGCATATATAATT
ATTACTACCTCTATTTTTAAAGGTTTTTTTTTTTTTTAATTTAGATGGCTATTTTTTCAAA
TATAAAAAAAATCGAAATGAGAGGACTTTAACGTAATTTTAAAGACGAAGAGAGGA
TAAGTTTAATTACATAAAAATCATACTCAAAGACGTATATTTTAGAGATCCAAGCAA
CTTAGATACCCTATTGAATCTACTCGATTGCAAGTGTAGACCATACAGTTTCATTGTTA
TATATACATCCGACATTGTCCTAACCTATCCAGTTGTCTCAAATCTTATTATATATTTTT

ATCGTGAAAGTCAAGTTAGGTAAACCCTGGTGACACAATAATAAAGACAAAGTAAA
TAAGTAATGAGTAGAGTCAATAATTGATTAAAAAGATACCTTTCCCTCTATCAACCCC
AAAATTACCTCTCATAAAGTACCATACATGAAAGAGAGAGAGAAAGAGAGAGATGC
TTATAACTTGCCAATTGCCATTACTCAAGTGAGAAAGCAAAGTGTGTGCAACTCCATA
AAGTATTTTTTTCCATTTCTCTCATCTTTCTTACTACTACTTGTATTACTCTTATTCAATC
TCTATTCTTTCTTCATTATTCCATGTTCATTCCCAACAACAAACTCCCCATGTTTCCAA
ACAATAATCCCATCTGTTTCATCCTTTATTCCTCTTTCATAATCATCCTTACGCTCACTC
TCTTCTGTTCAAACTACGTTACACTACCAATCAACTAATTATTTTAAAATAACAATTCA
CCCCGCTCTTGTAGTATTCATTGACCCAAAAAAA

>*CqSPL9*
GGATAGAATTTCAATGACCGAATAATGCAAAGTGGGTAGAATTTCATATTTTGGGCCT
AAATAGTATATGGAAAATTTCAAAAAATGGATAACGCATTGGATTGTTTATTAGGGTT
GTACACAGTTTGGTCTGAACCGTAACCAAATCGAACCAAACCGTATTTTAGCGGTTTG
GTTTGGTTTACGATTTATAAAAATGTAGTTTGGTTTAGGATTTTTGTAAATACGATTTA
CGGTTTGGTTTTGCTTTTCAAAAATTATAAACCAAACCGTAAACCAGTAAACCGAATT
TAATAATTATATAATTTTTAAATAAATCATAATAATTATATATATTAAATAAATTAAA
AATATAATAATAAAAACATTAAAATCAAAATACAACATAGGGTATACGTATAATATT
GTATTATGGCGATGATCAATTGATTGTTATTTTTAGTATGTGTTACCACTTGATTGTTAT
TTTTATTTGTAATACAATTTAATCCATTTGAGCTAATTATTTAAACTTTGTTATAAGTTA
ATGAAGTATTATTTGGAAATTTTTAGGAAAAAATGTCGTCTCATTATAAACTGCAAAC
CAAACCGTATTATTACGGTTTGATTTGGTCCAGTTTGGATATTAACACGCTTTGATTTG
GTCTGAAAAATTAACTGCAAAATTGGTTTGGTTTGGTACTCAACCGCACCAAACTGG
ACCGTGTTCACCCCTATATGTTTATAGCCATTTTTTTTCCTTCCAAGGATCACCCTTTTC
ACTTTGTCTTCTTTGACCCTTTGATATGTCTTCTTCACTTGATTCCTCCTCTTTCTTGACA
ATCTCTCTTATTCCCATCAATTTAATTTATATGGAGTACTTTATTTAGTTATCTTCTCTTA
TATTCCAAAACTTAGATCATATAACTTCAAATTCCCATTTAAAACTAGGTTTTTATGCA
CCTCATATCAACAAGGTTCTATTTGTACCTCCATTTCAAAGGACATGTTTAATGACTGT
TTAAAGTGTGTTATTTTAAGCTATTTTTCTTTTATTTGAATTGTATATAAGAGTATTTGG
TTGGGTGTAAGATGCTTTCCACAAAAATATTTTTCTCATTTTCTTGCATTTTTCTTTAGG
TTTTAATTCTTTTTGGGTGTTTGGTTTAATTTGTACATGAGTAAAAAAAATAGTTTTTCA
ATGGTGGACAATAATTCCATTCCTCACCTCACTACCTCCATTTTTTTTTCTCCTTCATC
CTCACCCTTCCACTTCTATTTTCTTTCTCTATAACTTTTCTTATACGAAGTAATGGAGAA
AAACAAAGGAAAACTACTTTACAATTTTGTTTTTTTTCTTAAAAAACCATTTTCATTGA
GATTGTATTATGTCAAATCATATGAAATCTAACTTAAACCTTATTATTACTTAATTTAT
CTTAACTTAAAATTCAAAGATAAGAAAACCTATAAGATGAGAAAAACAAAGCTTAA
ACTCAAAACTTCCATCTTCCATGATTGAGTTTCATCATCATAAAAATCGCCGTTAAAA
TCCGGACTAAAAAAAAGAACTACTCCATTAGATTAAACAAATAAAATCAAAGGGAA
AGAGGGGACCATAACGTCCATAAGTATTATAAGTATTAATTAATTGAAGTGAATGGC
TAATCTTCATAATATATAATAGATTAGACCATATTTAAATATTAAAGCACATATTCAA
AGTACAAAAATCTTCTGATTTTCTTTTCATTTTCAATTCTTTACAAATTTTCATCTTTTTT
ACCAACTGTCTAACCTCCACTATTTTTATATACTTTGGACATTAATTTTTTAATTTTCTT
ATACTCTTGTTAGTGAATTTCTCTAGTTATTTACTCCACTTAACAAGTACGGAGTAGTT
AATTAATAATATAAGAGTATAAAAAAGCAAACAAACAACTGCTTGAAAAAGTAACA

AAACTTGGAATTTAGAAAAAAAAAAAAAAAAAACAATTGACAAAATCTACAAGTTAA
ATGGACGACAAACATAAACAAAGCTAAAGCGTACAATCTTCGTATTCATTGACACTT
TTTTGATTGTTGATTCTATTTCTCTCTTTACACAAAAAAAACAGCTCGGAGCAACTGGC
CAAACTTTTTCGACTTTTAGGTGTAACTCTTTATACTCCCTTTACTCGAGGTCATCTGC
AACAAAGGACAATATTTTGGACTTTTAGGCAAAACTTTCAGAGGAGTTTTAATTGAGT
TTTGGTGGGTTTAATTAAGGTTGAAAATGAAGGGAAAAGGAGCGGTCCCACTTAATT
TTGTTTTGAGTGGGTTTTAGTTAGTGGGTAATAGGTGGTGGAGGGGACCACTAACCAA
AAAAATGGGTAATAGTGGGTAATTGATAGTTAAATATTACTCAAAAAGAAAAGAAA
AAAATTTGTTGCAAATAATATAGAGCACTCATTTTAGGTAAATATTGCAGATAATTGC
GGACGGAGGGAATATTAGATTCGAGTCGATTTCTATCGCACACACTTTTAACTATGCA
CCATATAAAGCATACGATATACGAGTACACATCTTTTTATTG
ACCCCACCATTCAAGGTGTGCCATCATCCTTCCTTACCTTACCTAACGACTGTCGATT
AATCGATTATATTATCTTCCATTTTTCTCCCGCAACAAATATGTCTCTCTGAATGAAAC
TCCACAAATTCAGAGAGACGAAGAACTAAGATTACTGTTGAAAGGTTTAATTTTTTAA
AATTTTTTGTCCAAAAAATTGAGGAGAGAGAAAGTGAGAGTATATGTACTGTTAACTC
GAGTTGTCATGGCCCATAACAAGTCGCTCTTGTCTGCTATCTTTCCAATTCCACACTAA
AAAGAAACAAACACCACTCTTTTTAAAATCCCACATAAACCCAGTTAAAAAAATTCA
TTCATCCCATTTCTCTCTCTAAAAAAATGTACCCAATTTCTCTCTCCTCAAAATCATGA
AGAATTGGGCATAAACAACCATACTCACTCACTCTC

>*CqSPL10*

CATTCCGGGCGCTCATGTGTGCCTCTGTTGGGTTATTTGCTGAAGGCCAGTTATATGCC
TCCTCATCCTCTTGAGGGGCATCCGGCTCCATTATAGACTCAGTTGAGTCAGATTGCT
GGACGGTTTCCTCACCGCCTGAGCTCCCGGACTCTTTTAGAATCTCATCTCCGGGCGT
GCTAGGTACGCACGGTTGGTCAAAAGGAGCATCATCAACCGGAGTTCCGGCTGAATC
ATCTGATTGGTACTCAGGGTCAACAACCGTTTTCCTTGTCCGAGCCATTCTGTCCTACA
CATGAACAGAGACGGTCGGATTCGTAGAAGAGTAAGGGTGGTCCAAACACCCCCAG
ACGGCATCCCCAATCTTAGACTTAGCCGTATCCGATGAACGGTTGACAACTGCCCTA
GGCCGTGCCGTGTGTGTGTTGGGAGAGCCCATTATTCTCTAACAACCTATACAAAAA
ACTCAAAAAGTTGCGAATGGCAGAGACGGTACCTTGAAGTAGTAAAAAGCTTGCTAG
CAGACGGATCTGTGAGTTGTTACTTGCAGATACCCCAAGCGGTACCTCTTTGAAGAGC
AACCGAAGTTTGCAGAAGGCTTGACCGTTTTTCCAAAATGTGGCTTGAAGATGCCAC
AGGCGGAAGAGTCCGGTTGTGTATGTTTCAAAAGCTGAAACACAAAACAAACAGTC
AGGCGTTTTCGAAGAAAATCACTTAAAGATACCCCAGACGGTATCTATAGAATGCAT
TTTTTGGAAGATTGCTTAAAGATGCCCCATGCGGTATAAGACCGGTTGTGGGCCTATG
GAGCGCAAATTATGAAGCTGCTTGAAGATACCCCAGACGGTATAAGACCAACTGTGG
GTCTATTGAGAGCAGTTATAAAGAAAATTGCTCAAAAGTGCCCCAGACAGCATGAGC
CCGGTTGTGGGTCTATTAAATGTATCTTTTAAAGAAAACTGCTCAAAGATGCCCCAGA
CGGCATAAGTCCGGCTGTGGGCCTATGGAATGCAATTTTTGAAGACAATTTTTTGAAG
ATGCCCCAGACGGTATATGACCGGTTGTGGGTCTATTGAATGCAAATTATGAAAGAA
ATTGCTTAAAAGTGTCCTAGACAGTGTAAGACCGGCTGTGGTTCTATTGAAATCAAGA
AGAGGAATTTGACAAAGTCAAACGGTACCTTAGTGTGAGGATCCAAAGAGCACTCA
ACCGGTATCCGGATGCGTCTGAAAGGAAGAAGCTGGTTACACGAAAGAGCAGCCGG
TATCAGAGCTTTTTTCTCTCAAGAATTTTTTTGTGAAAATTGGCAAAGTAACAAATGA

GAAGAGGGAGTCTCCAATTTATAGGAGGGGGCAGAAGGCGAATCGTCACCTCCCCCT
CCAATTCAAATTCGAAATTCGAATTCAAAAAAAAAAAAAAATGAGGGAAGGAGACGA
ACCGTCACCTTCCCAGAATGTGACACGTGTCACAGGTGCCACATGGCAAGCCACGTC
ACACCTATGAGAGGCAAATGTTTAGGGTAAAAAACCCTATGCCACGTGGCCCAATAG
CAAGCTGACACATGGCAGCCCTTGAAAGGCCTAATGGGATCCAATCGGATGCTGACA
CGTGGCATTGTAACCCTAATGAGGGAGCAGATCTTCATCTACTATAAATAGAGGAAA
CTCCTCATTTATTACACATTCAAAAATCCTCATAAATACAAACACCAAACACCGAGT
AATATTAATTGCAGAATCTCTCTGAATCAGAGAAAAAATCCATTTACTTTCCGCACTG
TTATACATCCGGAGACGTACTAACTTGATCGTCGGAGGCGAAACTGCACCCAACCGG
AGTTTCGTGGTTTTGCAGGTACAGACAGTCGGCGCGCATTTCCAGAATTGTAACTCTT
CCGAGGGACTCCAAAAACCTCCGAAATAGGTAGTAAAAGTCAAAGGATGGTGTATTT
TAGTAGTTGTAAAAGTGAAATTTTCCAAAATAAAAAAATAATTGAAAAATTTTGATA
TACTGTATTTAGTCCTCATTAAGAATGAAAATAAAGAGTGGTAAAAGAAGACAAGAG
ATGGAAAGGAAAGACAAGAGAAGCAATTAGAAATAGAAAAGTAATAAAAGGGAGA
CAAAGGAAGGAGGTGGGAGTGGTAAGGTGCGAGAGACACGCAGTGTGGAAGTGGA
CCCAATTACACCATCGCCCACTTCCCATTACTTTGATTATTATATTTTCCCGTGTTGGC
CCCATTCCTAATGTACCAAATATATTATTTAAATAATAAATTGGTACGTACCAGTAAA
TTTGAGAGTAGAGAGTTTTATACTTTTATGCCTTTGATTAAAATCGGAGTATGAATCAC
TGTGTCAACTGTCAACCCATCCTCAACAGGACAACAGATACCACTATTTACCACTACC
AGTCGTACAATTATTTACTCCTGCTCCTACTGCAGTAGACTCATAAAAAAAAAAAAGG
GAAAAGAAAAAAAAAGCATCAGATTGCTTGATTGATCGATCACCCACCTTTCTCTTCC
GAACCTTTCGTCCCCATCTCTCACTTTCTCTCTCCTCCCACTCCCCTTTTCTACATCTTCT
TCTTTTCTATTTACAGCAACCATTTCCAACCTCTACTCTTTCTTCTTAGCCCACCGACTT
CGTTTTTGCTCCTGTTTCTTCTGCTCCACCAGGTACCACTTTTTAAATTTAATTATTTGG
GTTTTCAAATATTTGTAAATTATCTGAAATTGGGTGTTTGATTTTTGATTACGAAGGTG
GTGCTCAGATTTGGCATGTGGGCAGCTATTCTTTATTGCCTATGTATGCTGGGCTGTAC
TGTTTCTGGACTTTTCGGGAAGAACCCCCAGCACCTTTGATTCATCGATCTGTTTTTGG
GGTTTGAGCTCCTTCTCTTTGTTTTTCTTTGTTAGTTATTTGAATTTAGTGTTTATTTGAT
GAAATTATTCATTGACAGAGCAGTACAGTAGGATTCATTGACAGAGCAGTACAATAG
GATCC

>*CqSPL11*
TTTGTGGCGTATCATGACAAGACCCACCACCCACAGACCACAGGAGTGCAAATGGA
GTAGGACCATGGCATGTGCAACCCATATGGAGCTATGGCTTCGTGTCATATTGATAAT
TAATTTTGTTATCTTAGTAGGTGGCTGGTTCAAATTAATCACAAATTTGTTGGCATTTA
TTACAAGTTTGTAGGTTTCTGTAAGAAGTTGCTGTGTTAATCTCGTGATTGATTTGTGTT
TGGCAACATCAACTAATGGTTTCTATTTCTTGCTATTGTGCACGAGGGATTTTTTTTTT
TTTTTTTTTTTTGTTCACGGTTCTATTTTTTGACTTGTGTATCTGACAATATTGACTTAAT
GACTTTGTGTAGATTATGGCAATGGAGATAGGGACACACCAGATCAGAGTTAATGGA
ATTTCCCGTGGCTTGCACATCCAAGATGAGTACCCAACTTCAGTGGGGAAAGAGCGT
GCGGAGAAGTTGGTGAAAGATGCAGCACCTCTGCACAGATGGCTAGATCCTAAGAA
GGATTTGGCTTCAACAGTTAGTTATCTAATAAGCGATGGCTCACGATATATGACTGGG
ACTACAGTATTTGTCGATGGAGGTCAGTCTCTGACTAGGCCTCGGATGCGATCTTATA
TGTGAGTCTGCTAGCTGAAATGGCAAGCTAGTCTACTTGGAGTCAAAATCTACAATTG

CTGAATATCATTTTATCATCACAGTTAGATACAACCTTTCACTATAGGAGGTATACTT
ATAGGAGGTTTAATTGTTACTGTATTTGTCAACTCATTATCTTTTTTGATGTAAAAAAA
AAGTTGCCATTAATATAATATAAATGATCAAGTTAATCATCCAGCTTGTTGGGCCCTG
GTTTTTGAGTAGGTGCCATTTATATCATTTAGAAAATTTGCTGATCGCTGATGCAACTT
TGAAGAAGCAATGTATGGCTTGGAATTGGAAAGGCAGCATTATATTTTAAAACATTT
ATCTAGTTTCGTTGTGCATCTTTTGAATTTTTGGTAATCAAATTTATTACTGATTACAGA
GCACTATTTACCGCGATTTTGAGTTAGTTTCGTATATTTGGACCTCCTTGTGAATAATA
AAAGCACGTGAATGTAGATGGAATTCTATTGCTACGACTTTCAAGCTTTGGACGAACT
CATTCACGATTAGTTGATTACCATTAATGTTATTTGTTAACATATATGGCACGTAGCAC
GCATAAAGGTGACATGAGATAATTATATGCCTTCTCTCACAATTATAATTTGCAATAA
CTAGTGTACATGTTTGTGAGTCTCCATGATGTCTAGCATTTTGTTATTTTGTTGCAAAAT
TGGTAGTAGTTTCAACTAGTTTATCAGAGGTCCCTGCCTTCTTCTGCTGAAGTGAAAG
ATGAAAACTACCTCACCCTATCTCTGTGTGGCCCGCTATTCATGCCTTTTTCTTCCCCT
GTTTCAATCACCCTTTTTGTTTGTGTGTTCTCTTCTGTCTTCAACTCACTTGTTCATTGAA
CCAAAAATAAAATTAAATGAGAGTACTAATTTTTCTTTTATCTACCACTTTTCAGAGA
AAATGAAATATATAAGACTTAGCTTAATCAACAAAGCATCCTCCTGAATGTCTTAAT
AGTGTTGTGCACTTAGTACAACCGGTTGTATAGTGGAAATTACCCGAGTAAGTGGTAT
GGTATGAAAATACACGTACAGCATACAAGTAATGGTTAAAAAAGAAAGTTACCCAA
ATAGCTCCCCTAAGATGGAGCAGGTATGATCTATTTAAGAATGGAGTGACTAAAAAG
TGGGAATGATTAGAAAGTGGAAAAATAGTGAGTCAATGGTTCCCAACCCCACAGTGG
GCATTGAAAAAAAAGGTAGATTCGATTAAAAATACTGTAGATTTGAAGTTCATACAA
CTGTTATTTCTCCTGTACTTGTAAATAAAAGAGGCCTACTGCATGCTTTATGCAGGGAT
TCAGAGAATTATTATCACCAATATAGTCCAAGTATCCTTGCAGAAATTTTTTATAAAA
AATAAAATTACGTGTATTTTTTATGTATGTATATACTTTGCAGGTTCTCTCCTATCATAT
GATCGGCCTCATTAAGTTCACCCTCTTTAGGAGAGTATCCTGATGGTTTTATATCCGGA
ATTATATACATAGGTTTAGATTATTACTCTATAAATACGGAGTTAAACCCGAAACCAC
ACTTTGCATCCCTCTAAACCCAGTGATAAGACAAAAGAGCTTGTCTTTTTACTTTCTAC
TGTAGTATCTAATGTGGGGGACAAAAATACTTGTGTGTCCTCTTCACCCACAACTAAT
GAATTGTCCTTGATTTCTTCTTAAGGGAACACAATTTTTTTTGCTCCCCCCCTACCCAC
ACAGTCATTTCCCTATCTCCCTTCCCACCTCATATTATTTCTCATAGTTAGTTTACCCTA
TGATCAATGGTTTTTCATTATCCTGATGTCCATGTCAGAAATATGGGTGGCTTAGGAA
GCCCTAATTTAGTGCCCTTCTGCTCAAGAACTTGATTGTAAGTTTACTCTCTTTTGATC
GCTTTTTCGATATTTAATTCTCTTCTTCATTGCTTTGGTACTTGGTTTTTATTATATAATC
ACATTTTGTACTGTCCATGCACTTTCTTGCAACTACTTTTGCCTTGGTATTTCAAAAACT
ATACTTTATAATGTGTGAAGTTTTGTATTCAGTGTTTTCAGTTATATGTATGATTTTTAC
AGGCTTTAGGTCTTACTTTGTATGTTGGTATTTTAGTATAGTTAGTAATTAACTTGCAGT
TCTTTTTCTTACTGTATTTTTGTATGTTGATGTTCAAGGTTTGTTTTCTGATGTAAATGGA
GTTATCAGGTATGGTGATTTAGACTGTATACCGACATCGGTCTGCAGTGGGGTCGTAA
ACATAGCAAGTTGTGTGGATAAAGGTTTATTGAGTGGATATCATTGTAGTTTGTAGGA
AGAGGAAAGGGGTA

>*CqSPL12*

GACAGCTTGATTCCTTGAAAAAGCATTAAATCCTGTCCCGGATTGCAACATACGAGA
GAAAATATCCATGCAGAATAGGAAAAGATACGGAGAGATAGGATCACCCTAACGAA

GACCACATTGTAGTTTATAGGTATCCGAGGTTGTACCATTGATCAAAGATTTGTAAGT
AATCGTAGCGAGGCATTGTTGGATCATTTGTAACCAACGAGCTAGGAACCCATAGGC
TTTTAAGACCTGCAAAAGAAAACTCCAGTGAACTGTACTATGTCATAAGATTTGCTCA
TATCAATTTTGATCGAAGCAAGCCAATTAGTGCCTGTTTTGGAACAATTGATTTTCTCC
ATTAATTCATGACTCGATAAAATATTATTCGTCATAACGCGGCCTTGAACAAAGGCGT
GCTGAGAATATGAGATTAGAAGAGAGAGATATTGGCGCAATCTAGCCACCAAGCATT
TAGAAGCACATTTGTACACCATGTTACACAGACTAATAGGATGTAAATGCCCCATCT
CTGTTGGATTACCGATCTTAGAAATCATGACAAGGAGTGACTGATTCCATTCTTTCAA
GAGGAATCCCATTTCAAAGAAACTTTGAATGACGAAAACCACTGGTTCTCCCACAAT
GTCCCATTGAGTTTTGAAAAATTCTACTGTAATTCTGTCTGGCCCCGGGGACTTATAGT
TTCCTAACGAAAACATGGCAATTAAAATCTCAGAAGCAGGAAGGGATGTTCCAAATT
AGCAATTGAGAGGAATTGAACCAAGGAAGATCCAACTCCCGCAGGACGGTATCAAT
TTCCGGATCATAAGGAGGCATGTAATCAGTTTTAAAAGTGTTCTTAAGAGATTGCACA
ACAACATGCTGAATATCCGTCTGCCCATGTTGGAGCTGAGAACACCAAGATCATCAA
GTAACGAGCTAATATCATTTTTCTTCTGTCGACATTTGACCTTTGAGAATAGAAGTTTA
GACGGGCAATCACCTTTCAAAATCCAGTTTTTTTTTCTTACAACGTTGTCTCCAGAATT
GTAGTTGCAATAAGATACTCTCCTTTGAAGTAACAACATTTGCCATGTAGAGAGAGG
CATCATGAACCGTGCGAAGTAACTCTCCCAAGATCCTTTGAGAAATGTTTCCAATTGA
CACCCCATAACTTTTTGTTATTGAAACACCAATTCCTGATTAGTGACTTGACGTGTTCC
AGATTATTAGAGAGAACGAACATGGGAGAGCCAGAGATATATATTTAATTATTTATA
CCATATCTCAATAATAAAATATGGAGTACAATAAATATGTGCAAAATAGCAAATCAT
AATAAACATAAATAACCAATTCTAACCATAAAAGAGTGCAAAGTTTAATCAAAATCA
TGTTAAACCTCAAAATAATACGACTATAATTTAAAGGTAATTTTATACATTCAACTCT
AAGTAATCCTTCTTAATCTCAATTTATAATGAAAATTACTATAATTGATGGATTGATTG
GGTCAAAAGACGGTCAAATTTAACATTGAACTAGAATATTTTTGAGGATGCAAACTT
ATTAAAAGGTACCAAGTGTTCTAATAAATTAAGGCCTTGTTTAGTTCAACTTATTTTA
CTTATTTCAGAAAAAATAAGTTCTGATCAGAAAAAATAAGTTCAGATCAAATCATCG
TTTTACATGCAATTTTTTTATTGCTAAATACTCTCTCCGTTTTTAAATACTTGCTCCAC
TTTTCTTTTGCGGCAGTTTTTAAATACTTGCTCTATTTCCTTATTTGGAAAGTTTTTATAA
TATTAATTCATTTTTCTCAACTAGTGGACCCAATTTTATTACTTTTAGATTCTCTCTCCT
CCAATTTACCTTCCACCAACCCCGCTATCTGCATATTTTAATACTTTCTCTAATTTCTCT
TAAAACTCCGCGCATGAAGAAATGGAGCAAGTATTTAAAAACGGAGGGAGTATCTTT
AATTTCATAATAAAAAAATTATGAAAATTACATATTATTAAAATACACATTATAACG
AATTTAACCAGATCTCACTTGATATATTTTTTCCCTACAAGTTATCGTACACTTAAGGT
TAAAGTTTGGTCAAAACAAACAATCAGATCTATTCAGACCAAATCGTTTATAATCAA
ATCAGACCATTTCAAATATAATCAAATCAGACCAGAACAGATAAGTTCAGATAAGCA
AAAATAACGGTGTATTAAACAGGGCCTTAATCCCCATATATATATACTCGAACACCC
TCGTATGGGCAATGGATAATTTGTCAAAATCTTTTGACTAAAACAGCCTAACATTATG
CCTGAAAGTTGTGGAAATTAAGATCGTTTTTTTTTTTCGGCAAAGAAAGAATTGACAA
AAAGCTCACTGCACCCTTCTCCTTCAATTCTATTACTCCCCTGCCTCCCAAAAAGCCA
CTAGAAAAGCAACCAGAAAAACCCTTGATTAAGCTTCAGACAATTGAAAATTTAAA
AACATAAAAAATTCCAAAAAAGAATAAATATTTATTCCTAGTACCTACTCCTTTATCT
ACTGTTTGCTTCATTACTCTATCCAAATACTGGTAATCTCTTTAAGTGCTCTTTTTATCC
AGTTTTGAGCTTCTTCTTCATTGCTTTTGGTTTTGTAAAGTAATTATTTGTACATTGAAT

GATCTATCTCTGAATTTTGACTTTTTTTCACTGAATTTTGATGGGGTTTTGCTTCTTGCT
ATCTGGGTTCTTTTGATGTTGCCTTTTGTATAATTGGGTTTCTTCAGAATTTTTATTTTTT
GCAAATTTTCAGCTTTAATTTGATGACTTTTGGTGTTTAACTACTAATTGATGGTTCTTT
TCAGTGGGGTTTGCGATTTTGTACCAAATTTTAGCTCAATTGTATGATTTTGATTGTGTT
TTCAGGTGCTAAACATCAAATTAAGAGAATTAATTGCAAAATTTTGTTGGGGAATTTT
TATGTGGTGTTTGGAATTTTGATCTTGTTGTATATC

>*CqSPL13*
AAGCAACCGCTAATATACAACAAAATAACAGTAACCATGATATACATACATGTAAC
ACTAAAAAGTAATCCTAACAAACCATTAAACAAATACAGACCTCAATTAACTCATAA
GAGACACTAAACTCAACAAAATTAAGCATTTGGAACAACAAAATACCAGTTACCAT
GATATACATACGAGTAACATTTAAAAGTAACCCTAAAAATCACTAAAAAAATAAAA
CCTATCAGAGGCACTAAACGGAATACAATTAAGCATTATCAACAGCAAAACCAAGT
CAAAACATAACAATCGCAAAAACAACAACAAAAACCCTAACATACATAAACGTAA
GCCTAAAAAATAAAACAACAAATAAAGACAACAACTAAGTAATCAAGTAACTCCTC
AGAACCACAACAAAAGAGAATTAAACATTTGCAACAACATAAAATAACTAGAAAAT
ACAAACAAAAACAAAATCTAAACAAAGATCAAAATTCAGAAAACAAATAGAACGA
GATATTGTGAGTGAAATCGTAAACCAAAACTTAGGTCAAACACTTGAAACCTAGGTC
AAAACACTCCCAAAAAACAATATGGAAAAAAAATTGAACAAAATACATATGATCTT
AGGTCAAAACACTCGCAAAAACAATAACAATGTCCAAAATTCTTAAAAAGGTACAA
ATTAGGATTTAAAAAATACTTGTTCCATCAACGATTTTACAGCAAGACGTTCCTTTCTT
TCCTTTGGGATTGATGATCTTCAATTTAATCATTTTCAATAATTCGAAAAACTATAGAG
AATTTAGAGAGAACAAAAAGAGAGGAAATTGCAGAAGGATAGTGAAAAATGCAGG
AGGAACAAGGAAATGGAACTAAGAAGTCTATAATAATGGTTGTTTGATTGAAGTGAC
TAGTCTCTAATAATGGCGGTTTGGAGAAAAGAAGATGGAAAGTTGCAATTTCAAATA
AAACGGTTGGAATTCTAAAATAACGGAGAATGTAATAAGCCCGTTATTTTTTTAATTG
AGGTCCATAATATGTTGGGCCTAAACACTATAGGCTCGTTATGTATTCTGGCCACACC
ATGTGGCCAGCCACATACAAAAATATGTAAATCCTTACATGTCATCTACGTAGCGCG
ACAATTTGTGAAAAGTTAAAACACTTTTTCTCATGACCCGACTAAAAGTTTAGCTTTA
TATATGAGTTATATGATGATTGTTGTAGCATTAGGAGTTTAGTATTAAAAGAAATATT
ATTGTGATAATATTTTAGAGGATATCTTATGAAGGAGATATGTTTTAGATAACCTAAT
AATTTAGGTAAAAAATTAAAGTCATTGTGTTATTATAAAAGTCATAAGAGGTTAAATT
TTCGTAATGATATTTTTTATATATTGGTCAATTTAAATAATAAAAGTCACTATATTAAA
ATTTGGGGCTCTATGTTTAACATACGTCATTCAGGTTCGCAATAACATAAAAGGTCAC
TCAATTTTTAATAATCAAACAATGTTAAAATCAAAGTTACTTTTTAAAAATTAGGGTC
ACTATATTTGATATAACTTTAGTTGCATGCGTATCGATGTTAGGTCCACAATAATATTA
TTGTGAACCACGTCCACACAAGTTTTTGTGCCTAATTTCATCTACATTTTTGTGAAGAA
GGTTTTTTTAGGAAATAATTCTTAGGAATAACCATTTTGGGTATTCTTAGGCATCAAAC
ATAATATTACCCAAGTTTATGCGTGCATTTTTTATGCCCACCTAAGGTCCCCTATTAAA
TACGGAGTACCTTTGTGCCAAAAAAATAAAATAAAAAATAAAACACAATACTTTTCA
ATTTTTTTGGGTTGAGCTAATGGACTAATTCAATTTATTTTTGGAATATATACCATATCT
CAATAATTAAATACAGAGTACAATAATTATGTGTAAAATAGCAAATCTTAATAAACA
TTCAAAAAAAAAAATTAACCAACTCTAACCATAAAAAGAGTGCAAAGTTTAATCAA
ATCATGTTAAATCCTTCCTAATCTCAATTTATACAAAAATTACTATTATTATTGGATCG

ATCGGGTTAAAAGTCGGTCAAATTTAACTTTGACCTAGAATATTCTCGGAGAATTCAT
CAAAAGACACCTATTTTTTTTCTTTTAAGAATAGTAGGCTAGTATTTATTTATTTTTTAT
ATCAAAAGGCACCTAGTAGACTAGTATTTTTTTTTATTTTTTATCTTTATCAAAAGGCA
CCTTTTTTTTGAACGGGATTTTTCAAAAGGCACTTAGTAGGCTAGTATTTTTTTTTTAAA
AGAAATAGTAGGCAAGTATATATAAACTTGCTTATTGTAATCCCCAAATATATACTCA
AACAGTCGAACACCCTCGTATGACAATGGATAATTTGCCAAAATCTTCTGACTCAAA
CCGCCTAACATTATGCCTAAAGTTGTGGAAATTAAGATCTTATTTGGCAAGAAAAGA
ATTGACAAAAGCTCACTGCACTCTTCTCCTTCAATTCTCTTACTCCCCTGCCTCCCAAA
AAGCCACTACAAAAGTAACCAGAAAAACCCTTGATTAAGCTTCAGACAATTGTAAAT
TTAAGTAATATAAGAAAATTCCAAAAAAGAATAACTATTTATTCCTAGTACCTACTCC
TTTATCTACTGTTTGCTTCATCACTCTATCCAAATACTGGTAATCTCTTTAACTGTTCTT
TTTATCCAGTTTTGAGATTCTTCTTCATTGCTTTTGGTTTTGTAAAGTAATTATTTTGTAC
ACTGAATGATCTCTCTGAATTTCGACTTTATTTTTCTGAATTTTGATGGGGTTTTGATTT
TTGGTATCTGGGTTCTTTTGATTTTGCTTTATTTATTACTGGGTTTCTTCAATCTTTTTGC
AAGTTTTCAGCTTTAAATTCAAGAATTTTGGTGTTAAACTTCAAATTGATTGTAATTGA
GGTTCTTTTTAGTGGGGTTTACAATTTTTGTGCCAAATTTTAGCTCAGTTGT

>*CqSPL14*
TGTCACCAGGAAAGAATCGAGGTGATTGAGGCTATATTAGTCAAATTCTGGGTAGGA
GTTGAATTTGATTCCAGTTACCTCAGTTTCTTGACACTCCTCTTCTAGTACAATAAACT
TCCTGTACTGCAATGTCTGCAATTAAACTTTTGGACTCAAATTCTATTTGTTTCTGTCCT
CCCTTCACCGACTCCTCTTCGTTCTTTTCTTTCTTAATTTATAATATTCCTAGAAATTAC
TGAATACAGTTAGTGGTTATGTTAAATTATCAGTCCGTTTCCCTAAAAAATAAAAGTG
AAATTTTTTTGCTTTCTTAAGATGTTGCTGACTGTTGTGCTATGGCTAGTATAATGGAC
CTAACAGCCCAAAATGCTTTGGCTGCAAAATACAAGACTATTTCTTAAAGGGTTTAGT
GTGGAGTTCTATTTTTCTTTCATTTTCATCAATTTAATTTCAGATATTGAAGTACTGGTT
TGGTAAATAGCATTTGTTGTTTCACCACCTGAAAGAATTACAGTTGGTTTTTCTTTTGT
GGCGTATCATGACAAGACCCACCACCCACAGACCACAGGAGTGCAAATGGGGTAGG
ACCATGGCATGTGCAACCCATATGGAACTATGGCTTCATGTCATATTGATAATTAATT
TTGTTATCTTAGTAGGTGGCTGGTTCAAATTATTCGCAAATTTGTTGGCATTTATTACA
AGGTTGTAGGTTTCTGTAAGAAGTTGCTGTGTTCATCTCATGATTGATTTGTGTTAGGC
AACATCTACTAATACTTCTATATCTTGCAATTGTGCACGAGGGATTTTTTTTGTTCACG
GTTCTATTTTTTTTTGACTTGTGTATCTGATAATGCTGACTTAATGACTCTGTAGATTATG
GCAATGGAGATAGGGACACACCAGATCAGAGTTAATGGAATTTCCCGTGGCTTGTAC
ATTCAAGACGAGTACCCAACTTCAGTGGGGAAAGAGCGTGCGGAGAGGTTGGTGAA
AGATGCAGCACCTCTGCAAAGATGGCTAGATCCTAAAAAGGATTTAGCTTCAACAGT
TAGTTATCTAATAAGCGATGGCTCACGATATATGACCGGGACTACAGTATTTGTCGAT
GGAGGTCAGTCTCTGACTAGGCCTCGGATGCGATCTTATATGTGAGTCTGCTAGCTGA
ATTGGCAAGCTAGTCTATTTGGAGTCAAAATCTACAATTGCTGAATATCATTTTATCAT
CACAGTTAGATACAACCTTTCACTATAGGAGGTATATTTATAGGAGGTTTAATTGTTA
CTGAATTTGTCAACTCATTATCTTTTTTGTTGTAAAAATAAAAGTTGCCATTAATATAA
TATAAATGATCAAGTTAATCATCCAGCTTGTTGGGCCCTAGTTTTTGTGTAGGTGTCAT
TTATATCATTTAGAAAATTTGCTGATTGCTGATGCAACTTGGAAGAAGCAATGTATGG
TTTGGAATTGGAAAGGCAGCATTATATTTTGAAACTTTGATCTAGTTTCGTTGTGCATC

TTTTGAATTTTTGGTAAACAAATTTATTACTGAATACAGAGTACTACTTACCGCGATTT
TGAGTTGGTTTCGTATATTTAGACCTCCTTATGCACTTTTTGGTGAATAATAAAAATAC
GCGAATGTAGATGAAATTCTATTGCTACGACTTTCAAGCTTTGGTATTATTAGTCACG
AACTCAGTACTTTTTTTTAATTACCATTACCGTTATTCATTAACGTATATGGCACATAT
CGCGCATCATGGTGACATGAGATAATTGTATGCCTTCTCTCACAATTATAATTTGCAA
TAAGTAGTGTACATGTGTGTGAGTCTCCATGATGTCTAGCATTTTGTTATTTTGTTGCA
AAATTGGTGTAGTAGTTTCAACTAGTTTATCAGAGGTCCCTGCCTTCTTCTGCTGAAGT
GAAAGATGAAAACTACCTCACCCTGTCTCTGTGTGGCCCGCTATGCATGCCTTTTTCTT
CCCCTGTTTCAATCACCCTTTTTGTTTGTGTGTTCTCTTCTGTCTTCAACTCACTTGTTCA
TTGAACTAAAAATTAAATTAAACGAGAGAGTACTAATTTCTTTTATCTACCACTTTTC
ATAGAAAATGAAATATAAAAAGACGTTGCTTAATTAACAAAGCATCCTCCTGGTTGT
GTCGTGTGCATAGTACAACCGATTATATAATGGAAATATTACCCGAGTAAGCGGTAT
GGTATGAAAATACCACGTACACCATACTAGCAAGGGTAAAAAAGAGTTACCCAAAT
AGCTCCCCTAAGATGGAGCAGGTATGATCTATTTAAGAATGGAGTGACTAAAAAGTG
GAAATGATTAGAAAGTGGAAAAATAGTGAGTCAATGGTTCTCAACCCCACATTGGGT
ATTGAAAAATAAAGGGAGATTTGATTATAATTTATAAATACTGTAGATTTGAAGTTCA
TACAACTGTTATTTCTCCTGTACTTGTAAATAAAAGAGGCCTACTTCACGCTTTATGCA
GGGACTCAGAATTATTATCACCAATATAATCCAACTATCATTGCAGAAATTATTTTAA
AACAAATTATTAAAAAAAAAGTGTTTTTTTATGTATGTACTGGTATATACTTTGCAGGTT
CTCTGATTCTGATATCATATTATCGGACTCATTATGTTCACCCTCCTGAGGAGAGTCTC
CTAATGTTTTTATATCCGTGATTAAGTACATAGGCTTAGATCACTACTCTATAAATACG
GAGTTAAACCCGAAACCACACTTTGCATCCCTCTAAATCCAGTGATAAGACAAAAGA
GCTTGTCTTTTTTACTTTCTACTGTAGTATCTAATGTGGGGGACAAAAATACTTGTGTTT
CCTCTACACCCACAACTAATGAACTAGTCCTTGATTTCTTTTTAAGGGAACACAATTTT
TTTTGCTCCCCCCCTACCCACAAAGTCATTTCCCTATCTCCCTTCCCACCTCATATTATC
TCTCATAGTTAGTTTACCTT

>*CqSPL15*
ACAAAACAATTACGAAGCCTCACATGAAATTAGAAATTGACATTTGACAAGGTGTTG
CGTGGGTCCCATTACAAATGTCAAGTGGCCAGGACCACTTTTGGGCCTTTTCTCCTCC
AATTAAATTCACTTTGGTACAACTTTTTTTTATATGGATAAAACTATAAAAGTACACTC
TACCATTTTTTTTTATTATTTTTTATTTTCAATTTTTTTTTAACTGTTGACAACTATACTG
GCACTGTTCTACTGCCTATTCTTATCAACTTCTAAGCAAATTTTAGGCGCTCGTTTGGT
TAGACACGAAAAATCCCATAAAAAATTGTATTTTATAGAAAGTTTTATTTTTTATAAG
AATTTTCACAAGTTAATTTGGTTATGAATATAAAAAATTAAAATTCTCATAACTTTTAT
ACTATGGGGACCTTGAAAATTTACTTGATGTTTCACTTCTTCCATTTGATTTTGTCTGTC
AAACAAATACTTAGTCTAATTGTCACTTTATACATCAATGTAGGATCAATTTTAGAAA
CTTTAAAGTGAGAATTTTCAATTAATCTAGTTTATTAATTTGAATTAAGTTAACCTATA
TGAGATTTCGATTTAATAGGACTCATCTTATCATCTACTCCGTAATAAATACTTGTATC
TAACAAATAAAAATTATATCTAATCGTACTATCTATAGGAACTTAGGTTGGCCTTTAA
AGGGTATGCACGTGCCTTGTGTTGCGGAAGCGAGGTGTGAAGTTGTCAAGTAAAAGA
ATAAAGAAAATGTAAGGTGAAAAGTGAGTAAAAATTAATCCACTTGTACTAATCAA
ACCAATGCATATGGTTAACTAGTTTTGGTCTTTTTATTAAGGTTGATTTTGGAAGCTCA
TGCACGCATGTTTTTTGGACTAGTTAGGTCACTGTAATTATGTCAATATCATTAACTCT

ATTATGCGGCTTATCATTTAAGGTTTCAATCAGGTTTAAGTTTTAGGCTGATTTTTTTTT
GTTTTTTTTTTGAGTGATTTAGTTAGGTTCAACAAATTTATTTTCTTGCAATTTGGTTTA
AGGTAAAGATGTTACATTGTTACGGAGTACTCCTTAAATAAATACGAGTATTTCGTAT
TGAAAATCTATATACCTATATTAAAGTGGAGCTTTATTTGGAGGAAATTTTCCCTCCTA
AACTCTATTTTTTTTTTCAAAATTATTAATTTATTAATTTATCTATAAATTTCATTATTTG
TTTATTTATGGTTGAATATTGACTTACATTTCTATAAAAATTTCTATATTTCAATTAGTA
ATCTATATTCATTTCAAAAACTATATACTATAAATTAAAATAATAACAATTATTTTTTA
TCTATTATTTGTAGGTCTTGAAAGTAAGGGAAACTTGTAATCTATACTATATATTAAA
AGGTATTACTAAACAAGTTTAAATGACACGTGACACTCTCCTAATTTAAATGAGTTTT
TCATTTCATATGTTAAAAAATATGGAATTTATAAGATTTGAACTCACAACCTCTTTAA
ACTTAAATTCTAACCTGCAATGTTAGCCACTAAGCTATTACCATATTATTTGCCATTTT
ATCAAAATTAAATATTATTAAGGATTTCTATCACGAAAATAATGCAGTACCTCAAAG
CATTGCCCGTGCCTATAAACTAGTTTCATGTTTAAAAGGCGAATCTCCCCCAGCCAAC
TGCTCTCTACACCAAACGTATTTGATTAAAAACAACTGAAAGTGAGGAAAACTTGAA
ATTTTAAGGTTAAAAGTCGAGATTTTCTCCAACCAATTACACCAAACCTTACTGGATA
AAAAGTTGCGTAATTGTCAACTTAATTTGGATAGAGACCTTGCAAATAAAAAGAGTT
CTCCTAGTTTGACGGTCTTCCAACACTAGATGATTTGAAAATTTTGAAGTGCACATGA
ATGAAGGTGAACAAAAAAAAAAAAAAAAGAAAAAAAAAAAAATAAAATTGTGATTAA
TCGGGCTCCAATTTGTACGAATGATTCACAGTATTCATAGTAAACGCAAAGTTGTACT
GTACTATCCTATAATTAAATAAAAATAATAAAAAGCGGTCGATTATTTAATTTAATTG
TATGAAATAATAATAATAATACAATAATAATATTTGAAAGGTTTATTCTATGTAATTT
GGTCAGACAGCTGCATCTTTTTCTCTCTCCTCTTTTTCTCTCACACATTCATTCATTCTT
CCCCCTTTCGCTTTGTCCCATTATCTCCTTCTTCTTCTTCTTCCTTCAGATCTCTTTTCTCT
CTTCTAGGGTTTTGAAGATCCAACTCTTTTATTTTTTTTTATTAATCCACTTTTTATTTTA
TTTTTGGTGTCCCTGATTTCTGATTTTTAGGCATTAAATGTGGAGATCTCATATTTAATC
TGTAATTTTTCTATTTTTTTCTTCATTTTTGTTGTTAATTTTAGGGATTTTAGCTCGTTTTT
ATGTATTAATACTTCTGATTTTGTTTTTGCAGCTGCTTTAATTAACTTGGTAGTGGATGA
TTTCTAGGTTCAACTCCTCTTTCAAAGGTCAGCTCTCTCTATTTTATTTATTTCAAATTT
AATAATTAAATGATAGTATAAGCTTGCTGATTTGCTGGTTTTTTAGAGATTGATTTCAT
GTGAATAAATCCTAAATTTCTGCTACTCTTTGCCCTATACTTTAGCCTTTCTAATTGGA
TATTAGTCTTTGTGTGACTCTAATTTTAGAAAATTTATGAATTTATTATTATTAAGTTTT
CAAAAATTGAAAAAAAAATGGGTTAAAAAGATTGAGAAAAGGCTAAAAAAAATTGAAT
CTTTTGAGTTTTGATGGATGTGTTGGACCATTTCCATTGTGTGTATTTAGAGGGTGATTT
CGTCTGCTCAATTTAGATTTTGTGAATTTTCAGTGTGTTTCTGGAATTCTGGGAAGTTG
GTATAGTAATTGATACACAAGAAAAGCCCGAGCACGAGTGAAAGAGAGGGTGTTAT
TGGAAA

>*CqSPL16*
ACTTATTTTTGTAATGAAATTTCTATAAATTTATCAAATTAATTAGGTTATAAATATAA
AAAGTTAAAATTTTTATGAGGGTCTATGTACATTTATTAAATTGAATTGAGCCGAGCT
GAACTGAATTAAATTGATCAGAGCTAAACTGAACTAAATAGAGCTGAAATGAATATT
TTATAAGAGAAAGAAATTAGGAGTTGAACTAAACTGAACTGATTGGAGTTAAATTGA
ACTAAAGTAAAATAAAATTAAGCTGAAATGATCAGACCTTAAATTTTCATTGGCTAA
GGAGCTTTGGTAATTTACATAATTTTTCATTATTTTGTCAGTCAAACAAATACTAAGTC

TTATAGTTAATTTGTATTTCAATATGAGATCAAATTTATGCACTTTAAATTGAGAATTT
TCAGTGAACTAATTTTTTTTATATTTAGGGTATGTTCTCTTACGTTGAAATTCAGTTTAG
TTTAGTTTAGTTTAGCTCAGCTCAGTTCAGTTCAGTTCAGCTCCTAATTTCCTTCTCTTA
TAAAATATTCAGTTCAGTTCAGTTCAGCTGAGTTCAGTCAATGAGAACAGGCCCTTAC
TCTGTATTTCATAATACGAAGAAATCAAAATTCCTTAGAGAATAACTGAAGGCATTTC
TGATCTAGACAGTAGTTGACCTATATAAAATTCCGATTCAATAAGACCCATCTTATAT
CATCTATGGATTACTCCGTAATACGAGTACAAGTATATCTAACAAAGGAAAATATCT
CTAAAAAAAAAAAAAACTAATTGTACTATCTATAGGAAGGCTGGCCTTTAAAGGGTAT
GCACGTGCCTTGTGTTGCGGAAGCAAGGTGTGAAGTAAAAGAATAAAGAAAATGTAT
GGTGAAAAGTGAGTAAAAATTACAGTAATCCACTTGTACTAATCAAACCAATGCATT
TGGTTAACTACTTTTGGTCCTTTTATTAAGGTTAATTTTGGAAGCCCATGCACGCATGT
TTTTTTTGCTACACATACAATGGTTGTATGTGGGTTATCACATACAATATCATTGTATG
TATGCCACATCAAAAATGGACCAAAAAAATAAGAGGGAAAAAAGGTGGGCTCCAA
AATGCAAATCAATTAAAGGCTACCAACCATTGTATGTGTAGCCTTTTTCGTTTTTTTGG
ACTAATTTGGTCACTGTAATTATGTCAATATCATTAACTCCATTATGCCACCTATCATT
TAAGGTTTTAATCAAGTTTAAGTTTTAGGTTGATTTAGTTAGGTTCAACAAATTTATTT
TTTTGCAATGTGGTTTAAGGTAAAGATATTGAGGAGTAATCATTAAATAAATATGAGT
ACATATTTTCCCTTTTAAACTCTACCTTTTTCTTTTTTGCGAAATTATTTATTTATCTACA
AATTTCATTATTTATTTATTTATGGTTGAATATTGACTTACATTTCTATAGAGATTTCTA
TTTGATGAATTTCAAAAACCAAACATGGCTCGGGAATTATATGGTATAACTAAGAAT
AATAAAACTTTTAATAAAAATCTATTTATTTGTAAGTCAAACAGTTGGAAGTGAGATT
GCGAGAGAAACTTGTAATTTAATGGTAAAAAGGTGAATCTCCTCCAACCAATAATAC
CAAACCTTTTACAGTTATACTCCTATTTGATAGAAATGGTTGAAGGTGAGGAAAAATT
GTAATTTCAAGGTTAAAAGGCGAATCTCTTGCAACCAACTACACTAAATCTTGCTCGA
TAAAAAAAAAAACTACATAATTATTTAATCGATCTGAATAAGAGAGAATTTGCAAAT
AAAAAGACTTCTCCTAGTTTGACGGTCTTCCAACACTAGTTTATGGTTTGAAAATCTTG
ATGTGTACATGAATGAAGCTGAACAAAAAAAAAAACTGTGAATAATCCGGGTCGAAT
GTACGAATGATTCACAGTAATACAGTATTGATAGTAAACGCAAAGTTGTACTATTCA
ACTATAAATAGTAACTTGTCAAAAAAAAAAAAAAAAAAAAAACTATAAATAATAAAA
ATTAAAAAAATTAAACAAAAAGCGGTCGATTATTTAAATTGTACTGTGTATGAAATA
ATAATAATAATAATAATAATAATATTTGAAAGGTTTATTCTATGAAATTTGGTCAGAC
AGCTGCATCTTTTTTCTCTCTCCTCTTTTTCTTTATCTCTCACACATTCATTCATTCTTCC
CCCTTTCGCTTTGTCCCATTATCTCCTTCTTCTTCCTTCTTTCTTCAGATCTCTTTTCTCTC
TTCTAGGGTTTTCAAGATCCAACTCTTCTTCTAACTCTTTCATTTTTACATTTATCCTTTT
TTTATTTTGTTTTTGGTGTCTCCGATTTCTGATTTTCAGGCATTAAATGTGGAGATCTCA
TATTTAATTTGTAATTTTTCTACTTTTTTTCTTCTTCATTTTTTGTTGTTAATTTTAGAGAT
TTTAGCTCGTTTTTATGTTTTAATTCTTCTGATTTTGTTTTTGCAGCTGCTCTAATTAACT
TGCTAGTGGATGATTTCTAGGTTTGGTTCTTCAACTCCTCTTTCAAAGGTCAGCTCTCT
CTATTTTTTTTTAAAAAAAAAAATTAGATAATTAAATGATAGTATAAGCTTGCTGATTTG
CTAGTTTTTAGAGATTGATTTCATGTGAAAAAATCCTAAATTTCTGCTTCTCTTTGCCCT
ATACTTTAGCCTTTCTAGTTGGATATTAGTCTTTGTGTGACTCTATTTTTAGAAACTTTA
TGAATTATTATTATTAAGTTTTCTAAAATTGAAAAAAAATGGGTTATAAAGATTGAGCA
AAGGCGAAAAAAATTGAATCTTTTGATGGATGTGTTAGACCATTAACATTGTGTGTAC
TTAAAGGGTGATTTCGTCTGCTCAATTTAGATTTTGTGAATTTTCAGTGTGTTTCTGGA

ATTCTGGGAAGTTGGTATAGTAATTGATACACGAGAAAAGCCGAGCGCGAGTGAAA
GAGAGGGTGTGGTTGGAAA

>*CqSPL17*
GATGGTGAAAACAAAATTTGTAGTAATCATAAGAGACTTTGGAGTTTCACACGGTTG
ATTGAGAAATGAAGATACCTTTAAAATCCAGTATTATCTCCCCTATTTAAAGTCATTA
ATGATTGATGAGGAGAAAGAAAGGATCCTTTGTTGTGCACAATATAGTGTTGTCCGTA
ACTATGTGAATGAAAGAAGAAAATGTGTGAGCCTACCACTCTCTTCCACTTGATAAT
ATGATGGCATTCAAATTTATTACCACCTCTCTTAAATTCGTACGTTGTATAACAGTTCT
TAAATATACATATATGTTTCCATACATGTGTTCATGAGGCCATGAACTATGCTGGTGC
GTTATGATCTCTTGATTGTATACATTGTATGTCCTTATATATGTGCAAATTTTCTTATAC
ATGTGCCCTCTAAAACCATGTGTTTTATGAGTGTTTCAAATCTTACACTTATGTATTAT
TTCTCCATCCCCTTAATATTGCAAGATAGAGGTTAGACAGATATTTTAAGAAGAGGAA
AAAGATAGAGACCTATGTTATGAGAAAAGAGAAAAAGTGAACATGTATAAATAGGA
AAATGTTACAATATTAAGGGAATGGAGGGAGTATAATTTAATAAGTATAGACTAAAA
CACATATGTTTGGGTGCATGATAATATGGTATAGGCAGTCTATTATCAATAATTGGAT
GTTCGTGAAAATTGCAAGTATTAGCTGACCATTTATGTTGATGGTAATCATCGCCGTG
TTCATTGTCAACCTAGCTCTAATAAATGGAGTAAAAAGCTAAATTTATTGGAAAGGCT
AAAGACAAAGACAAGTACTTGTATTTTGAATTGAAAGGAAGTAAAGATTTTAGTGAT
CAACCATTAGAAACATCTTAGGAAGCCACGTCACTGATTTCCTCTTGTACGAACTCGT
AGTTTAGTCACCAATGCATACCCCATTGGAGATGAGCTCTGATTCTAAACTCTAAAGG
GGATTTTGTATTCTTGCAAACCTTCCAATTTCCATAGTAGAAGTATTATTATTTATTAG
TATTAATTATTAATAAGTTGGACATCTAAATAAAACTAAAACCAAAAAAAGAAATAT
GGGGAAAGATGAAAGAAACAAAGAAAAAACCCAAAGAATGAGACATCATCCAATG
TGAGACACACTAAACACAAGAAAGTACTCAAGACCCATACCCCCCTTTTCTTTCCTCC
CCTTTCTTTGTACTTTTCAATTTTTTTCACCCAAAAAAGTTGAAAGCAACAACACCCTG
TCCCTCTCCCCTCCACTCATTTTTTTAATTATTATTTTTACCTTTCAAAATCCCATTTATT
CATTATTTTACTAACTTACATTTTTCTACTTACTGCAAGGCTACAAACCCTTATATAGG
AAGTCCTAACTCCCATATTTGTGAACATTATTTTACACATTACTCTCTACTTTCTACTTA
TATTTTCCTTTTCTTTTCTTTTTTATTTCAACTTTCTACTTATATACTCTGAACAAAGTAA
GCCTAACTCTTTTCTTTTTTGCCTTCCTTAACTTGAACTATTTTCTCTCTCTTATTTGCTT
ACTTTTAACTCTTTTCACTCTCTAATTAGTAAGTGTAGTAGTCTTAATAAATTTTCTGCT
TCTACGTTAAATATAGGCTTAGCTTTTCTTTTCTCTTGTCCTGCTAAAAAAGAAAGGCT
ATACTATTAAGCACAGCTCTTGCTTTGCTTTCTTCTCTATCTCTGTCTCTCTCTTCCTGAT
CCACACTGTTGTCATATTATTGGTTTGTAATGGAATCACTGTCAGAGAATGAATGGTT
GGGAAGCCCAGATCAGTGTCCTTGTTCTGCAGCTCTTTTTTGTAAGTTTTTTTTTTTTTT
TTTTTTTTTTAAATAAAATTATAAAAATAAAATAGGCATTATTAGCTGTTATACTAGTT
TTACATTACATCCCCTGCTTTCCTCTTTTTATTTCCTAATTGTTTGGGCTTTCTCTATCCT
TCCAACTTTCTTGGATTATCTCTGCCCCAATGTTGACAGTAGTCTAGAAGTTTGTTATA
CAATCTGCTTGGTTTCTGAGAAGGTTTTTTTTTTTTTTTTTCTATAATTTCCCAGGTATC
TATTTTTGTAAATTTTACAGCTTTTCTTCAATCTTCTTCTCTTTTATATCCTTAATTTAGT
ATGCTTACTACTCTGATACCTGCTGTTATTCTTCTTTTTTATGTCTCTCCTGAAATTTGCC
TATTTTGATTCCATTTTCTCTCCAAAAAAACCTTTTTGTAGTTTTGTGTAACTGGGTTTT
TGGGGATGCAGAAATATGACTGTGGAATTTGGAATTAAGTTAATAGCAATAATAGAG

GTCACTCTCCAATATTGCCTATTGAAGCCATACCCATTCTTAGCTGTCCTGTATCCAGC
ATAAAATTACTTTCTGGTTTATTTGTTGTAGTTCTTACATTTTTCATGTGGTTTGATACTT
ATCTTGGGTGAAAAAAATCATGTGTACCAGTCGAATTAAGTAACTTGATTTTATGTAT
TTATAGATTTACTTCTATGTTCAACTAAACGACTACGTTTATAATATTACTTGCACTTC
ACTTATGACTGGAGGAGGGCTTTTTGGGGCTCTTTCTGTCTCTCCCTCACTTTGTCTTA
ATCTTTATTGGTTTCTTAAGATTAATTTAATTCAATTTTGAAAATGACTGACTTTAACA
TATCTTTGTTGTTTTTATGTTAGTACTACCTCAGTGGAGCCTATATATCCTAATCATTAC
AAAGATTAGGTATTCACCAAATCTAGTCTAAAAATCTTGGATTAGTAAAGACTATCA
ATATATGTCTGTGAACTATTCTTGTATTGGCTTTTTTGAGACAGAGCAATGTTGAAGAA
TTTCTGCTAAGTATAGGGTTAATTTTCCTCATATCATATTAATTTATTGGTGTTACATTT
TTATTACAGGAAGAAAATAGTAGGGTGGACAAAATTTGGGAGGGAGAAGGTTATTAT

>*CqSPL18*
GAAATGAAGATACCCTTAAAATTCAGTTTTATCTCCCCTATTTAAAGTCATTAATGATT
GATGAGGAGAAAGGAAAGGATCCTTTGTTGTGCACAATATAGTGTTGTTGTGTTGTCC
ATAACTATGTGAATGAAAGAAGAAAGTGTGGGAGCCTACCACTCTCTTCCACTTGAT
ATATGATGGCATTCAAATTTATTACTCTCTTAAATGGGTACATTGTATAATAGATTAAT
AATTCTTAAATATGCACATACTTCCTATGTTTCTATACATGTGTTCATGAGGCCCTGAA
TTATGCTGGTGCGTTATGCTCTCTTAAATTTATACATTATATGTCCTTAAATATGTGTAG
ATGAAGTGTCTTATACAAGTGCCCTCTAAAACCAAGTGTTTTATGGGTGCTTCAAATC
TTACACGTATGTATTACTCCATTCCTTAATATTGTAAGATATGGATTGGACACGGATTT
TAAAAAGAGGAAAGAAGTAGAGAGGAATATAGATAGAGACCCATGTGATGAGGAG
GAAAAAAAAAGAATGAAAACATATATTACTAGGAAAATGTTGTAATATTAGGGGAT
CAGAAAAAGTATAATTTAATAAGTATAGACTAAAAGTTTTAAACGCATATGTTTGGG
TGCATGATAATATGATGTAGTTAGTCTATCATCAATAATGTTTTACGTAAATCAATTTT
AGATAGATAATTAGATATTGGATGTTTGTGAAAATTACAAGTATTAGTTGACCATTTA
TTTTGGTGATAATCATCAGTGTTCATTGTCAACCTAGCTCTAATAAATGGAGTTAAAA
GCTTAATTTATTGGAAAGGCTAAAGACAAAAGAGAAGTACTTGTATTTTGAATTGAA
AGGGAAGTAAAGATTTTTGTGACAAACCATTAGAAACATTTTAGGAAGCCAAGTCAC
TGATTTCCTCTTGTACGAACACATAGTTTAGTCACAATGCATACCCCATTGGAGATGA
GCTCTGATTCTAAACTCTAAAGGGGGTTTTGTATTCTTGCAAACCTTCCAATTTCCATA
GTAGAAGTATAATTATTTATTATTATTATTAATAAGTTGGACATCTAAATAAAACTAA
AACAAGATGTAGGGAAAGATGAAAGAAGAAAAAAAAACCCAAAGAGTGAGACATC
ATCCAATGTGAGACACACTAAACACAAGAAAGTACTCAATACCCATACCCCCCTTTT
CTTTCCTTCCCTTTCTTTGTACTTTTCAATTTTTTTCACTCAAAAAAGTTGAAAGCAACA
ACACCCTGTCCCTCTCCCCTCCACTCATTTTTTTAATAATTTTTTAAAATATTATTTTTTT
TCCTTTCAAAATCCCATTTATTCATTATTTTACTAACTTACATTTTTCTACTTACTGCAA
GGCTACAAACCCTTAAATAGGAAGTCCTAACTCCCATATTTGTGAACATTATTTTACA
CATCACTCTCTACTTTCTACTCATATTTTCCTTTTAATTTTGTTTCAACTTTCTACTTGTA
TACTCTGAACAAAGTAAGCCTAACTCTTTTCTTTTTTGCCTTCCTTAACTTCAACTATTT
TCTCTCTCTTACTTGCTTACTTTAAACTCTTTTCTCTCTCTAATTAGTAAGTGTAGTAGT
CTCAATAATTTTTCTGCTTCTACGTTAAATATAGGCTTAGCTTTTCTTTTCTCTTGTCCTG
CTAAAAAAGAAAGGCTATACTATTAAGCACAGCTCTTGCTTTGCTTTCTTCTCTATCTC
TGTCTCTCTCTTCCTGATCCACACTGTTGTCATATTATTGGTTTGTAATGGAATCACTGT

CAGAGAATGAATGGTTGGGAAGCCCAGATCAGTGTCCTTGTTCTGCAACTCTTTTTG
TGAGTTTTTTTTTTTTTTTTCTTTTTTGGTTTTTGAAAAATTATAAAATAAAATAGGCATT
ATTAGCTGTTATACTAGTTTTACATTACATCCCCTGCTTTCCTCTTTTTATTTCCAATTGT
TTGGGCCTTCTCTATCCTTCCAACTTTCTTGGATTATTTCTGCCCCAATGTTGACAGTAG
TCTAGAAGTTTGTTATACAATCTGCTTGGTTTCTGAGAAGTTTTTTTTTATTTCTCAGGT
ATCTATTTTTGTAAATTTAACAGCTTTTTCTTCAATCTTCTTCTCTTTTATATCCTTAAAT
TAGTATGCTTACTACTCTGATACCTGCTGTTATTCTTCTTTTTTATGTCTCTCCTGAAATT
TGCCTATTTTGATTCCATTTTCTCTCCAAAAAAACCTTTTTGTACAGTATTTTTTTTGTA
ATTGGGTTTTTGGGGATTCAGAAATATGACTGGAATTTGGAATTAAGTTAATAGCAAT
CATAGAAGTCACTCTCCAATATTGCCTATTGAAGCCATACCCATTCTTAGCTGTCCAG
TATCCAGCATAAATTGCTTTCTGGTTAATTAGTTGTAGTTCTTACAAATTTCATGTGGTT
TGATATCTTGGGTGAAAAGATCATGTGTACTGGTAGAATTAAGTAACTTGATTTTATG
TATTTATAGATTTACTTCTATGTTCAACTAAATGACCACGTTTATAATATTACTTGCAC
TTCACTTATGACTGGAGGAGGGCTTTTTGGGCCTCTTTCTGTCTCTCCCTCACTTTGTCT
TAATCTTAATTGGTTTCTTAAGATTAATTAATCAATTTTGAAAATGACTGACTTTTACT
TATCTTTGTTGTTTTTTATGTTGGTACTACCTCAGTGGAGCCTATATATCCTAATCATTA
CAAAGATTAGGTATTCACCAAATCTAGTCTAAGAATCTTGGATTAGTAAAGACTACC
AATATATGTCTGTGAACTATTCTTGTATCGGCTTTTTTGAGACAGAACAATGTTGAAG
AATTTCTGCTAAGTATAAGGTTATTTTTCCTCATATCATATTAATTTATTGGTGTTACAT
TTTAATTACAGGAAGAAAAAGTAGGGTGGACAAAATTTGGGAGGGAGAAGGCTATT
AT

>*CqSPL19*
TTTGCGACCGTTTTTCATACGGTAGTAGAAAAACGGTCGCAATTTAGCCGAATTTTAG
TAGTGATTCCTCCAAGATAAGAGGAGTTACATGGATCAGTAAATTAGAGATGAAGAG
CTTTAATTTGCAAAAGATTTGAGCAGATTTGGCTGAAAGGAAACGACCAAGCTCAAA
GTTCGGAGCAAAACCAAGCAACAGTGGCAGCAAGAGCAGAATAGGGAAGACTCGA
CTCCAGCACTAGACGACAAGGCTGTCGTCTAGCAACAACATCTGGCGTCTAGAAACA
TATTTCAGCGTCTGGCTCTAGATCCCAGCGTCTGGCGACAGAGCCAATTCTTGACTCC
AGAGATATTTCAGCAGCGACAGAGCTCGAAGCCTCTAAGAAATGTGATTTCGACTTC
ATACAGCAATTTCGGGTTCCTTGGTACGCCAGTTAGCCCCTATATGCAATTTGACATG
ATTGAGTAGTTGGTTTGATTACAGTCTAAGTTATCGACCTAACGAAAGATCGTCTCAC
TTAGCTTAAGCGCCTAAGTATTGGCTCATCACAAGCGCCAAATCATCGGTTCAAAAG
CAAGCACTTAAACGACAATATTTCCAAATATAGATTGTCGCGCATTCTTATTAAACGA
CAAAGATTTCCAAATATGGATTGTCACCCCCAACTGACAATATTTCCAAAAATGGATT
GTCGTGCATCTTCTTCAACGACAATTTTTCCAAGATTGGATTGTCATCTCTGGCAAAGT
TTCCAAGCGTGGATTGTCGTGCCCCTTCTTTGTTTGGCAATTTTTTCCAAGTTCGGATTG
TCATCGCTGGCAAGTTTTCCGAAATTGGATTGTCATACACCCAGTTCCCTATAGCAAA
ATTTCCCAAAGGATTGTTGTAGGTTTCTATTCATCATAAATATCGACAAAAGTTCCTC
AACGTGGATTGTCATTCATTTCAGTAAGTGGAAAATTTTCCCGAAGTGGATTGTCGGT
CGACTCTTTTTTGCAAATGTTCCAAGACGCAGATTTTGTTATTAACGTGAAGTACTTTA
ATTGATTAATTGATATTATGATGTAGAAATCATGTTTATATTGTTATTATGATATTCCT
AGTATCATGTCTAGGTTAATTTTAATATTAAATATGATTCCTATGATGCTTCCTATGTT
CTTCCTAATATTTATGATTGTTAGTATCATGTTAGGGTTAAAGATTTGATTTTTATTGCT

TAGTGACATGCTAGGGTTTTTTATTAATCTTATTAGTATCATGCCTAGGATCTTGCTAT
GTTTATTATGCTTTAATATTAACATGAACATGATTATGATTATAATTGTTATAATTTAT
GATGATTATGATTATTATTATTATTATTATTATTTTGATGATGATATTATTATTATTATT
ATTATTGATTATGTTGCTTGATACATTATTTATTCTTTATTGCTTGATCACCTTTTATTGC
ATGTTTAAAGGGAATTGGTACTTGCAATCACGCTTAATAAGATAATTAATAATCTTGT
TTAGGGTTAATGTGATTATAATTCAAGTGATCGCCAATAATAGATTTATTTGTGCATTA
ATATTCAACCAACATAGATTAAACAGGTTTAGGGTTAATCCTAATGATTGCATGTTAA
TCAGAAAGCGCACATGAATACGATCAGAATAAAGTAGAGGCCTATTTTTAGGGCAGG
TTCAATCTCTTTTAGGTTGGATTTGTACCTTCATCCCCGAACCCTTATTTTGTTCTAGAC
CTTATGGAGTTGATGATCTCTTGGATCGTTAAGGGGTGTAACCTTTACCTCGTGAACG
GTTTACAAGTTCCTAAAAAACTTGTTGGCGACTCTTTTGCTCTATCTTCTTCCCTTTTGG
ACGCCACCGTACTTTAGTGTATGATCACAGTGGGGTTTAAGCTAGTGGGATTTTGAAG
GCTTGAAATTATTCCCTACATTTATTTATAGAGAAAATAAGCCAAAAAGATAAAATA
TCAAAACAGAGCATTTGGCGCACGCAAAACGAGGTTGGCGCACCTAAAATAGAGCT
GGCGCCAGCACCAACTAATTGGCGCCAGGAGCCAATGGCTAGCACCAGGCGCCAGG
CCTGTTTTTGACGCGTTTTCGAAAATCAGATAAGTACTGGAGGATAAAATCGTATCTT
TTCCTCGAAAATGCTCCTTGATTTTAATTAAAACTCTGAAAAGTCAACAAAAGTCAAT
TTATCAATGATAATTGAATTAGGGATGAAAAGAATATTCTAGAAGAATCTAGAAGAC
TAAAACCATTCCTAATTGAATTAGGAATGATAAATCATATTAGGCCCTCCCCTAGATC
ATTGGGCCATACATAAGGCTCAAACTGGGGCCCAGTTTAGTAAATTGGCCATAACTT
CCTCAATATTCATCGGAATTGGGCTTATAATATATCGTTGGAAAGCTCTTGTCAAGCT
CCATCCAACTAAACTATTATTGGACCCATATCATGGACCAACCAAACTGGATTGGCT
GTTTTCTTAAAATAAGTTTTCAAAAACAGAGAGAAGATAAAAGAAATTAAAATTATC
CGCAACACTAGACTTTCCACACTTTCACTTCATTCGATTATGATTCGACGTGAAAGGT
ACTCATCACTGGGTTTGGTTTTGGGGAGACACAACTGGGTGTCTACAGACATTGTTGG
TTGGATTGTCATTGGAGAGAAGCCGTGGTGTTGGGTGTTTCAATGATAGAAGAAGAC
CGGGAAAGACGTGCCAAGTGAAGGTGTATCATGAATCTCACATGGTGGTTGTGAGTG
GTGAATGCACATATGGTTTTAGCATGTGTGAAAGACAAGTATGGGTGCTTGTTGAGGT
GGGAGCATGGTAGGAGTGTTCCATCAACAAAAGGAAAATGCCCAAAGGCTTGTGTTT
GTCACGGCGGGTGAGTTCGTGTAGAGTGCGGTTGGAGTGCTCGGAGAACTAGGAAAA
TCCCTTGGATAACGTGATGTTTATCAAAGTTGGTGTAGTCTTTG

>*CqSPL20*
TGCCTTGGCAATAACATCACCCATTTTTTATTGAATCAATTTGGAACTTAATTTATTGA
AGATTAACGAAGACAAATATTTATCTTCTATTATATAAGACAGTATGGGTAGTATGAT
TTTTTCATTTGGACACCACATTCCCTCTCATTGGCTCACAATAAAAGGATTAATAGGC
AAAAATTTGCTTATAATTGAAGGACTTTCAAACATAAAAATTGATTTAGGGTGACAAT
AGTGATATAATGAAAACTTTTTCAACGGCATTATTGTGGTAATTTCAATTAAATGGGG
AAGCTAAGAATTGTGAAAGTCGTAGAACGAGATAAAAGGTATTATTAGAAAGGAGG
CAAAGACATTGTGTTTGTCTATTGAATGTTTTTTCTCCCCTATAATGTATTTCGTATTTT
AAAGGGTTTTTATAGATTTTTTAGAAACCAGTGAACTGATAAATTTGATCTAATTCGT
AGCCTAATGATTGGTTGAGCTTTGTATGAAGGACATATGTCCTAATAAGCTGTGTTCTT
ATTATCATTTTAAGGCAATAGCCCAAGATTATATCATAACAAGATTTCCTTGGGTTAT
GATTTCAGTGGATTGCTTAGATGTTGCCACTTATCAAACTTGTCATGTATGATTAATGT

CTAGTATTATTGGCTAAGAGGCACATTAGACGTAAGTCTAGTTGTTGGAAATATTTTTT
TATTTCCTACACCAATTTCCATACTAAAAGTACATGAACAATTCACTAAAACACTTGG
AATTGATATATTGTTACAATATGTCAAATGAGGGCACATTCCCCCAAAGTAAAGGGT
GAATTTGTTACCGATGTGAACGATCCAATGTTATGAAGCCAAACTCATTTTAACCAAG
AAGGGGTTGTAACTAGCATTATGTGTTTAATAAGACATGTAGGTGGACATGTTCATAA
ATTTCCATTTGCGGCAGGCATTGTTAATCCATTCCTAAATGCGCCAAAACCATATCAT
GTCATCTCAATTACGAATGCACACCAAAAAAATTATTCGATTTGAAATTGGGATCAA
CAAGAAATGGATTTGAAATCTTACCATCATATAATAGTAATTAATTTTTAATTAAACT
TTTGATTTTATTTATATGGTGTGAAGTTAGTATATAGTGAGAAATTTTTATCATAAGTA
GTATAATAGACAGTATAGCGGGTCAAAAGTAAGATAAAATAGACAATTAAAAAAAA
ATGTATCTTGACATTCATGGAAAAAAATGTATCAACAAACCAGATACGATTTTATAA
GTAAAGGATGTGTCGTCACTATGTCAGTTAGTTAGAAGGAACATAAGAATTGAGGTA
TCCAAAACTCCAAAAGGGCTACAACACACAAGCCTTGCTTAAATTAGCAAAGGCCAT
CATGATAATTAGATTAATTACAAATATAGTAGTGAAAATGTCGAATTCATCTATCCAA
AGTGCGTAAAAACTATAATATAACATCCAAAGTAGCAGATTAGTGCATCGTTTGAAT
CTCACACACAGAGTTGTAATCTACTTGTTTATATAGAAGTATACGTTCACACATAAAA
CATTCCCTCTAGTATTTAAGTGCCCCATTTTAACTTTTTTATTATTCACATATACAACTT
TGACTATTGTCGGTGCTTTATACATAAGGTTAAACATAATTATCTAAGTTTTTTTAGAA
TCGACTCAATATACAGTTTTCGAATATCAACTTTACATAATTTATACTTATTGATAATT
AAAGATATAATTAATCAAAATTCTACGTTGACAAATACACTTGTCAAAAGTTAAAAT
GATATTTTCTTATATCATTAGTATATATTTATATCATTAATGCATTCATATTGGCCCAA
AAAAAAAAAAAAAAAAAAAAAAAAAAAAACATTTCACAATGCTATAATATAACGCC
CCTATAAGCAAGAATCTAGAACCTAAGTAAAAAGGGGTTGAAAATTCAAACCTTAGT
TCTATTGTGGGGTTGAAAAAGACATGTTGTGGTTAAGGATAATTATTGGACACAAATA
TTAGTGGACCCCACCTAGAAGTGGACATACATCTGCCTCTTTAATGGGCGAAACCCG
GTATTGTGGCCCTAATATCTTTAATTCCAATGGGGTATCCTCAAACGAAACAGAATTT
TGACATTCCACCTAAATAACTGAATTAGTATCAATCTTATTTCTTAATCTTGTTTTTTCT
ATACCTTTCAATTTCATCATTAACTGAATTTAGTGGGAAAATTAATCCTTAAACATCCT
TTTCTTTTTCCTTTTATTATTCAGTTCGATATAAAATACTCTGTGTCGTCCAAGGGTTAA
AATTAAATCAAGATTTTATCTCATGTGTTGGATACCACCATGCAACATTTTACTTTTTA
TTATTTTTTTCGACATAAAATACTCTGTGTCATTTAAGAATTAAAATTAATTGAGATTT
GATATCATGTGTTGGGTACCATCACACCACGGTTTGGTTACTGTCCACAATTATTTTTT
GTTTGCACTGTTAATTTATAAGTTGATTCTAAGTGATATATGTGAGAATGACGGACAT
GAAAGATCCTTGAGACATGCTTCCCCAATGAGATTTTCTCAGAAATCTGCCTCCACTT
TCCTTCACTACCTTTTACTTTCTTTATTTTAAAGAATAAAATCCTCCCTTTTTCTCTTAC
GCTCTGTATTTCACTCCATCTTCTTCCTTGCTTCACTATAACTCCGGTATGTACAAAAC
CCATCTCCAATTTTTCATTAATTTCATTGGGGTTTCTCCATTTATTATATTTATTCACTCT
AATTTATGAAATGGGTTGTTAAAAAAATATATGATTTTTTGGGGTTTAAATGGTCAATT
TTGAGTTGAAATTATGAAATAATTGAGTATTTTTGGTGGGTATTTGTTTATTATATAAT
TTTACTGATAATTTCTTATTTTTATGGATTGTATTTGTTTTGATTATGCTTGATTATAGAA
TTATATGATATATGCAGGTCAATTGTG

>*CqSPL21*

TATTATACATTCCACAACCCTTGGAATACCCCGTATGGTAGAACCTCCTTGCAAAGCT
TGAAACAGAATACATAAGTTTTTTCTAGCACTAAGGAACACCAATCATATGACTCAG
GGTCCACTGCTGCAATTGCCGCTGGAAGTAACTTAGGGTACAACCTATGGGATGTTGT
AGGACACGGAACATCCACTAGGGCTAGCAGCAAGAACGCAACCTTAAACTCAAACA
CATCTTCAAGTGGAGATTCAACAGCTTGTACTAAGTTTTTCCTCGGAATCCCATTCATA
TTGCTCGGGGTAGTCGTACTGAATTGAGAAAGGACACCTTCAAGAAACTGCTTTGTG
GCATCATCCATAGCTTCTTTCGGAACCGGCAAATACCCCTTAGGAATCCCAAGAACC
CAATGGACTTGAACGGGGTCAACTGTAAACTCTAGCCCATCAGGATTTATCAAGCAA
TATGACAGAACATCAACCCTCGTAGCAAGCCAAAAATACAACTCCCTATGAAGTTGC
CTGTCAACTAAACCAAACATTCTAGGAAATCCCATCTCTTTTCTTTTCACAAAACTCA
TTAAAAATCATACCAAAATACTCCACCAGTAAATTTACATTTTCAGGCCGCATATAAC
AAAAAACACATATCAATATACACATGTCATGGATGCAACTGATAATTGAATTAACAC
CATTTTACCCTTGTGACCTCCACCTTGCAACGTTCAATTTATTTACCAATTTCCTTCATC
TTTCTGCCTTCACTAAGTTTATTGTCCGAAACAAAAACAAGAAAAAAAAAACAAAAA
TAAATTCACCTACAATGGCAACACTTGGACAATACAACGAAAAACTCACCCGGTACA
CGGCTTCATCAATTTTCTTGCACACCTTAAAGAAAAAAGCAATTAATTATCATCGCCC
TCCAAACAATTAGAGATCAACTTCTACATTTATGTATGTTACCGATAAATGTGATACC
CTTACCTTTTTGCTACCGACCAGCAGATTCCACTTTAGAATTTAATTACTTCTCCACCA
TCCTTCAAATTGAGTACACAAACAATATTATAACCAAAATATACAAATGCAAACAAA
AAGCAACCAAAATATACAAATGCGTGAACTTTTTCACAAAATATACAAATGCACGTA
ACTTTTTCACAAAATAAAATCCACCCCACCAACTTACGCATGTAAGTTAGAAAAATG
TATGATGACTCAATAACATACAACTATCAAGTATGGCCACACCACTTATAAATTAAA
ATGCCACCAAATAAACTATAACAAACTTCTTAAATAGGACTAGACATTAAAATGATG
AGAATGAGGGCCACCATTCTGAATAATCCTTCAACTCTAATCAGATCAAACTTTCAGT
ATCACACCCCTCCTCAAAATGGCCACAACCACCATTGATTTTAAATGCCATTTTAATA
CGTACTAATCATCATGTCCAAAATGTCCAAAAGAATATAAGACTTTCATTTAGCAACC
ATTCATTAGTCAAAATCTTTTCAATATACTATATAAACAAATATTCAATAATAATTATT
ACAGTAATAAACTTCATTCAACAACTCACCATTAAATCTTTTCAATATACTATATAAA
CAAAGTTCATTCAAAAACCACTATTAAATAGCAACGACAAAAGGAGATAAAAAATC
AACTACGGATGCCAGGCACTTAAATTAAAGTGAAATCACCAAACTTAGAAAAATTG
AAGGGTGGTAGCCACTATGCCAATCAACACCATTTTCGAATATTCAAAGTTAACAAA
TCAGATTACTAACTGAACAACTCATAAGAAAGCAAATTATCTGATATTTATTTAACTG
AACATGTTTAAGCATTCGTAGATATTCATTCATAATTCTATACAAAGAATAAAAATAC
ATTTTACTCAATTACTAATAAATTACCTGAATACAACTTCTTAAATTCACCGTGTTGTA
CCTTTTCGACTACATGACCGCGCGTACGTCCACCTCTTCTTGCACCCCTACGACCACC
CCTTCCACCCCTCATATAACTTGCTACAATTAACCCCTTAGTAACATAAACCCTACAA
TTCTTATGAATCCTTTATATAAAAATAAATAACTTGTATTACCTTACATGCCTCCATTG
TTGCGAGAATGAGGTCTACACCACCCACCTTCGAATTACAGCAAACACAGATCTCTT
CAACAAAAAACCTTGAATTTCATGACAAACCTTGTTACGATATAACCTAGACTAAAT
TCGACACTTTTGAGGCCATTTTCTGACGCTAGGTTTTGACAATTTTTAGCGAGATTTAG
GCTAGCTCAAATACTTACTATTATATAAAATAAATTAGGGAGACTTTGTATTAATTAA
TTAATGACTTGATGTTAGAATTTTTATTTTAATCACAATTTTGAAATTTTTTATTTATAT
TAAAGCTTTATTTTTATAATTGTATTAGGCGCTAAAAAATATTGTATTATAATTCGGAT
TTTCGGATCGGATCCAAATAAAAGAATTTTAATTTGGATTGGATCAAAAGAAAATTTG

GATTGGATCGGATCAAATTGAAAAGTGCAAACCGAATTATATTTTGAATTGGATCGG
ATTTGGACTAAATCCAAATCGAAAACCGAACAGACACCCCTTGAGAAATTCTCGTTG
GTGTGTTGGGCGTTAACACCACGCATAGTCTAGGATAGAAAGGGAATCAATCCATCA
ATTTATGAAATCAAATCAAAGAAACAAAAATGTCGTCATAATTTTAGCTGAATTGATT
TACTTTTTAGACCAAACAGATAAAATAAAATTCAGAAATTCAAAAGTCAATCATATT
TTCAGACCAACAAAACCACCTAATCGCTGACTTCTCACCTACAACTTCACCTGCTGCT
CATTCTTGCTTTCCACCCTTTCCAATTTTCTCCATCTAATTACCTCTTTTACTATTTCCCC
CAAATCTCCTAATTATTCGATTAAGGTTAGGGTTAGGGTTTGTGTTATTCCGCC

>*CqSPL22*
CCTCTTTGGAGAAAAAAACAAAGCTCTTTAAAAAAAAAAACCCTCAAGCTTCCTCTT
CAAGATTCTCCATTAATGTGGGATTGTACTACTCCTCCGCTCGCAAAAGTACAAAAGA
TCAACTCTGCTGAAGGTATATTCCTATTTTTTGTTGTGTTGTTCTTGATTTTGCCATTAG
TGGCTTCTTTGATATAGAATTTTGGTCTTTTTTTTTTTTTTTGAAATTTTTTTTTCATGATT
TGGAGACAACATTAGGGGTTGGTTACACGTTCATGTCTTACCAGCAGAAAGGGTTAC
TAGCTGCAGTGTCTTCTGTGTTCCCATAAGCTGAGAATAGGATGTGTGCTAGGCATGT
GTACTGTAACTTCAAAGCGGTTTTTGGTGGGGACCTGGAATTCAGAAAACAATTATGT
ATAATGACAAAAATTAGCACTGAGAATGAATTTTTCAAAAACATTGAAGTTATGAGG
TCCATTTTGTAGATGCTGCAGAAGATTTGCTAAACAGAAATTACAAGAAATGGTGCA
AGGATTTCTACAATTCAATGTCATGTTGTGATAGTGTTGATAGCAACATGAGTGAGGT
TTTTGATGCCTACATTCTCACCTCTAGGCATAAGCCAATCATTACCAAGCTTGAGGAC
ATTAGAGAAGGATTAATGGATAGACTACACAAAAAAGAGATGAAATTAGAAAGAAA
GAAGTCATCTTGTGCCCAAGGATACAACATAGATTAGAAAAGAACAAGATTTGGGCT
AGGGGCTAGAATGCTTTCTAGGATGGGGCCTTCTGCTATGGGGTGAGGCAGGGGGCC
ACATAGGAGAAATTTGTTGTGAACTTGCAGGAAATGACTTGCAGCTGCAATGCTTGG
AAAGTTAGTGGCATACCCTACAAGCATGTTGTGGTAGCTATTTGGAACAAGGTGGTC
CAACCAGAACACTATGTGAATGACTATTTCAGCAAAGAGAAATACATGAAATCATAT
GACTTCCTGCTAAAGCCTTTGAATGGTCCTCAAGAATGGCCACCAGCTGATTCCTTTG
TGATTCCACCTAAATTGAAAAAGGTGCATGGCAGACCTAAGACTAAGAGGAGATATC
AATCTGGAGAGACCACAACTACTGGTAAACTGAGCAAAACAGGAAATCCTTATATCT
GCATCTTATGTGGCTTGAAAGGGCATAACAAAAAGGGCTGCAGGAATGCTTCAAGAG
AAGAAAGCCATCACAAACCCACAGGAGAAGTGTCAGCAACTCATGCAGATGCAACT
AATACATCTATTCCCATACATATGAGAGGATTTGGGGTCTACACATACCCTAATGACT
GTGAAAGAGTTGCATCAATAAGTATCTCTTACTTATTCTTGTATATATGGTAATTCTTG
CAAATTTGTTAATTTTATAAGAGGTCTCAAATAAACACATACCCTCTCAACAACAACC
ACAAAGGGCCTTCTACATTGACATTGAAAGAGAGCAAACAGTGTACTCCTTCAAGTC
AGGATTAATTAGTACCCTATGTCAAGTCAACAACAAAGCCAAAATACCAACACCATT
TCAAGTCAAGCATTGCAGATATAAAATTAGATTTCCACCACATAAACAAATTCATTTT
ATCGTTAATAGAGTAAATTCCTACACTTAAGTCATTTTTCATTACAGGATTTAGCTAGA
AAGAAGAAAATTAGCAAGCAATAGCTGCATCAGGTACAATCTAATACTTTTTAATGC
TTAAATTGTTCATTTCTTCAATGCAAACCAAATTCACGCCACAACCAACACAAGAAC
AACCTTCCATAGGTCACAATTAATCCCCAAATTTATCCTAGAAGCCCTTCTTATTTTCA
ACATCAAATAATCACTTTCAATCTTCAATTCCTTGCATACTAGTTTGCTTGCTTCAATT
CCTTCCTCATAGACATAACTTCATTCTTAAATGAGTTCCTGCTTCTCTTGCATAAGCCC

CAATATAACCTCAGTATGCCAAGCATTGGGGTCTTCCATGTCCACCCACTTAAAAAC
TTGCAGCCCCTCCTTTCTGTTTCTGCATCATAGTCAGGGCAAGCAAGGAACCTTCTACT
AGGGTTGTCCTTTGTCCAGGCCCTGCTCTTAGACAATAACAAGCCACAGTGACATCTA
CTTCCTGATATGCTCAGCCTTGACATGTTTAAAAAAAATTGTTCAATTTTATCTGTAAT
TTTTTGAAATGCTTCTTATAATTATTTATTTATTTTTTTCCCAATTTGATGCAGTGTCTA
TCTCCTTGTTGAAGATGATTATAGGGCATCATATATATGGAAGCAGCTAATTTTTTGA
ATTCCAACAACTACTTTGTAACAGAAAATTGTGAAACTCTCCAAACATTAGTACCTTC
TAATAAATGTGTTAGTAGTTTATTTATTTATTTATATTTATTTATTTATTTATTTTTTGGC
AAATGGTAACTGGTTGTAATGGGGTAAATACTTGTTTGAGTACTCTACTTGCAACTCT
CCAAACGTGAGTATTCTATCTCCAACTTTTCTCAAATATCACTCTCCATTTTAGTTTGC
TCCAACAACATTAGATAACAATACCAGCCTAAAGGCTAGAGCGGCTAATATTTTCGG
TTATTAGGGTCCTAAAGAACGCGCGTTATAAAAGGTTGAGAAATTCTCGTTGGTGTGT
TGGGCGTTAACCCCACGCATAGTCTAGGATAAAAAGGGAATCAATCCATCAATTAAT
GAAATCAAATCAAAGAAACAAAAATGTCGTCATAATTTTAGTTGAATTGATTTACTTT
TTACACCAAACAGATTAAATTCAGAAATTCAAAAGCCAATCATATTTTCAGACCAAC
AAAACCACCTAATCGCTGACTTCTCACCTACAACTTCACCTGCTGCTCATTCTTGCCTT
CCACCCTTTCCAATTTTCTCCACCTAATTCCCTCATTTTCAATATCCCCCAATTCTCCTA
TTATTCGATTAGTGTTAGGGTTAGGGTTTGTGTTATTCCGCC

Supplementary File S4

Similar SPL sequences found in Bacterium TMED181 and *Micromonas commode*.

>tr|A0A1Z9KGZ5|A0A1Z9KGZ5_9BACT SBP-type domain-containing protein
OS=bacterium TMED181 OX=1986654 GN=CBD41_09240 PE=4 SV=1
MRKPPGQPPRSERGRKRKRLRSRRRCAHGPGPFPRVSTHVPACFHPRRAPHLELARSPPPT
RRVRRVVGHRPLFLARGSHSRLFPRRGGVPKPKNSPLTASLRTLPIVPQCRVKGCGATCHT
VHEVRARACASHCAALYVQLDDQPEGTQSRFCYQCHKFHTMDEYVTPDGSLLQRHNCY
KSQQRRLRRRRDKSNEKAAASKTNGGRGRGRNQPNQPGANPTAYFLSRAAGVQARRVA
GADGQSQSRGGAGGTAPVTPSPLYATQKSGDMPPPPPRRPSGGVLIEPVTYANYEEPVAKT
YQELLPSAMQAAEAEASGAGAAGSAMNTNPGGEGGGAGGGAXMTEEXMRRLRNEAVR
AHNAQTLRFGSFTESSLKPRTAPSVNPA

>XP_002508836.1 pe-pgrs family protein [Micromonas commoda]
MANEGVDWALSFGDGADVDDDGPDVNAQAPGPASRGGAGGANEKAPQEPKTCRVTGC
DAICQTVHEIRARACASHCAALYVQLEEEPEGTQSRFCYQCHKFHTMDEYITPDGSLLQRH
NCYKSQQRRLMRRRNKSNEKAAANKTHGGRGRGRNQGANPQAYYLSRAAGANRRSSV
AGGAEGGIGAEGGGGGAPGTPSPMYAHARERNEGPDAGAGGMMPPPPRRVSGGVLIEPV
TYANYEEPVAKTYQELLPPGMRAEAEEASRNTAAMDAMPAGPGEMTEEMMRRLREEAV
RAHNAQTLRFGSFTESSLKPRTAPSAS

Supplementary file S5

***SPLs* genes identified from *C. pallidicaule (*A sub-genome*)* and *C. suecicum* (B sub-genome)**

>AAA00870-RA

MPYDNTPPQIPLFSDPRGFPTAGSSSLLTLESLSSVMQIPKTEPDLFTNRPIGLNLGRRT
YFSADDDLVSRLYRRSRAVEPALHSPKCQAEGCNADLSHAKHYHRRHKVCEFHSKASTVI
AAGLTQRFCQQCSRFHLLSEFDNGKRSCRKRLADHNRRRRKSNNQRITSTAPSMSSGENA
QSSPSQTILRSPSDSGMNSSSSVTIAVSPPRVSFDSSFMQVRSFE

>AAA12591-RA
MEAEMGGKTDVYCSAVMPVSDPKAVGKKSLEWDLNDWKWDGDLFLAAPLNTTHTPLD
CRSKQLFPLGPELASNNNVGSEESNKTNEKEKREMEKRRRVVVVPHEELSDEGRPLNLKLG
EQVYPIAEDEADKLEGKSVKKSKSAGTGTSQPACQVEGCTADLSNAKDYHRRHKVCEVHS
KVSEAFVGNVMQRFCQQCSRFHALPEFDEGKRSCRRRLAGHNKRRRKTLPETSPNVGSLT
DEKSSGYLLVSLLRILSNLHSNGSDETKDQDLISQLLRNLASQVSGSNLPELQQGSQSLHNA
GISIGVPEKNPSSAQELCQAVPSAEARIGLLTRENQHQKEKTQCASQPGIFHPTDGSIAT
KGSVPGAYLGIDLNNVYDDSQECVDIPGNGHGSIACAFRSEHLQKSSLPQTSGNSDSNSG
HSSSSGSDSQGRTDRIVFKLFGKDPNDLPNQLRTQILDWLSHKPSDIEGYIRPGCIVLTV
YLRLNKSLWEELCYDMSSSLSRLLSLSDDPFWKTGWIYIRVQQSAAFICDGRVVLDTPLP
FKSRGSRISSISPIAVPTAETVQLVVKGSNLSGPTSRLLCAIEGKYLVQDSCYSLVESTA
AEHDEIQSLSFHCSIPNVVGRGFIEVEDYGLSGCFFPFIVAEPEICSEICMLERVMEIAG
TDEGIERRNDALEFIHEMGWLLHTNRLSSMSGQTNIHLDLFPFVRLKWLIDYSMDHDWCA
VLRKLLDLLFSGIVDTGNHASVENALSEIPLLHTAVQRNSRSMVEFLLRYIPKKVKNTIG
SEQKQSYYVPSSFLFRPDVTGSNGLTPLHLAASCAGFKNMLDALLEDPGMVGIGAWENAR
DSTGLTPKDYAYLRGHNHYIDLFQSKVNKNSSGKHVVVDILGLSNLEYKQKQSDELKLAK
FNSLYTEKRQISQNCKLCEQRPNYGFRGTSLTCRPVVMSLVTIAVVCVCTALLFKSMPRV
CYVFVPFRWDSLKYGAM

>AAA00069-RA
MTEQAHRLSELRKRGIEWDLNDWKWDGDLFIATPSNSQGQQFIPLVPAPGNSSNTSSSCS
DDVDDGTGRRDLERKRRVFVVDQDSLEEAAPLTLKLGVPHRERDHWETSTAKKTKLPSSS
STRAVCQVEDCEADLTKAKDYHRRHKVCELHSKATKALVANVMQRFCQQCSRFHALQE
FDEGKRSCRRRLAGHNKRRRKTQPETAVQGNSIDDQTNSVLLMSLLRILSNMHGNNRAN
QTTDQDLVAQLLKSLANSSGLHSGKGLSGLLHESQKLLNGGMANGNGHSEKMSAYLSND
QQDTPRVIDQHVQLPDSEIPRKGLYPANSRGSEIQAVSLEEPKSLFPIKDSPPAYSETTEGRM
KLNNFDLNDAYVDSDDGMEDLERSPVNENFATGSVDFPSWARQDSHQSSPPQTSGNSDS
ASAQSPSSSSGEAQGRTDRIVFKLFGKEPNDFPIVLRGQILDWLAHSPTEIESYIRPGCIILTIYL
RLAESSWEELCSDLSSRLTRLFDISDDTFWRMGWVYVRVQNQIAIVHNGEVVLDTS
LSLQDNNCCRILSVMPIAVSMDEQVQFKVRGFNLSQSTTRLLCALEGKYLDQEASQESEV
GDFLEDDDETEHANLSCIIPKATGRGFIEVEDHGLSSSFFPFIVAEKDVCSEIRTLENVLELKK
TDEEAYEINNTLESWCQAMDFINEMGWLLHRSHLKSRLADLDPNTVIFSFRRFKWLMDFS
MDHDWCAVVKKLLDILFAGTVGLGEHSSLKVALSEMGILHRAVRRNSRPMVEFLLRYAPL
NVSEEFISSNDGGQVKFFFRPDAQGPAGLTPLHVAAGRDGSEDILDALTDDPGKNGIDAW
KNARDSTGATPEDYARLRGHYAYIHIVQRKIYRSSTSGHVVVDIPGEQSVAPRQDGALSFEV
GRSASLALNQSCKLCDRKKMSVYYGSRSRASLVYRPAMLSMVGIAAVCVCVALL
FKSMPNVVCLFQPFRWEMLNYGSS

>AAA03639-RA

MTEQAHRLSELRKRGIEWDLNDWKWDGDLFIATPSNSQGQQFIPLVPAPGNSSNTSSSCS
DDVDDGTGRRDLERKRRVFVVDQDSLEEAAPLTLKLGVPHRERDHWETSTAKKTKLPSSS
STRAVCQVEDCEADLTKAKDYHRRHKVCELHSKATKALVANVMQRFCQQCSRFHALQE
FDEGKRSCRRRLAGHNKRRRKTQPETAVQGNSIDDQTNSVLLMSLLRILSNMHGNNRAN
QTTDQDLVAQLLKSLANSSGLHSGKGLSGLLHESQKLLNGGMANGNGHSEKMSAYLSND
QQDTPRVIDQHVQLPDSEIPRKGLYPANSRGSEIQAVSLEEPKSLFPIKDSPPAYSETTEGRM
KLNNFDLNDAYVDSDDGMEDLERSPVNENFATGSVDFPSWARQDSHQSSPPQTSGNSDS
ASAQSPSSSSGEAQGRTDRIVFKLFGKEPNDFPIVLRGQILDWLAHSPTEIESYIRPGCIIL
TIYLRLAESSWEELCSDLSSRLTRLFDISDDTFWRMGWVYVRVQNQIAIVHNGEVVLDTS
LSLQDNNCCRILSVMPIAVSMDEQVQFKVRGFNLSQSTTRLLCALEGKYLDQEASQESEV
GDFLEDDDETEHANLSCIIPKATGRGFIEVEDHGLSSSFFPFIVAEKDVCSEIRTLENVLELKK
TDEEAYEINNTLESWCQAMDFINEMGWLLHRSHLKSRLADLDPNTVIFSFRRFKWLMDFS
MDHDWCAVVKKLLDILFAGTVGLGEHSSLKVALSEMGILHRAVRRNSRPMVEFLLRYAPL
NVSEEFISSNDGGQVKFFFRPDAQGPAGLTPLHVAAGRDGSEDILDALTDDPGKNGIDAW
KNARDSTGATPEDYARLRGHYAYIHIVQRKIYRSSTSGHVVVDIPGEQSVAPRQDGALSFEV
GRSASLALNQSCKLCDRKKMSVYYGSRSRASLVYRPAMLSMVGIAAVCVCVALL
FKSMPNVVCLFQPFRWEMLNYGSS

>AAA17771-RA
MESWWFDSLDKGFESNEAISQSDAIVRGKNVLIGWEHKSTNEDSVLTPSQQSVENRSYSE
LGIAEMVRRQCPTDSTRNELEDNGSVGDFYSSFVTTNAFSGDDESSSKFSSSVMDSSSRE
SSLIDLKLGGFGDNPNSSSTRTATAHVLSSADSPTPPKRARVSSQAVHCQVYGCHKDLSS
AKDYHKRHKVCDVHSKTPKVIVNGIEQRFCQQCSRFHLLGDFDDGKQSCRTRLAGHNER
RRKPQVGFSNRNGRSFQSYTGSNFQGFTPTSTSFICQDILPRGISHTVKYGTNDWVKHIKV
EDGTDCTQMPTYSCINRQLQPKSILPPHDFKKQFPFIDNTNNTGTQFPFGKSVNQHTPEI
VSHSLFQTNSPRNEDLALLDAASTVQELAGISESGCALSLLSFQSQNSSGHSSAMPGCHP
VIIPSSSPQYSVNEVSEKIFGNGAQALKSEVQNRYSSVIVSSTGRNQPSFMSVLNYGYNA
HSEFGNEIHHRSKFMNNKDHLLCEDGTTIDLLQLSSQLQRMENQKLSEPLKQETENYFCL
RMT

>AAA00083-RA
MGYKFTKNYCDLGELKKGGIGSISAIFGSSSFDTEKAIGERLVDLKLGRLGEFGKGLVDN
FKGHSNSIMDPFSPTAGSSKRPRTPGTTSQVVSCLVDGCKADLSKCRDYHRRHKVCEMHS
KTPRVTIGGNEQRFCQQCSRFHPLGEFDEGKRSCRKRLEGHNRRRRKPQPEPLPVNQGNF
FSACQGSRFLAFSNQPIIPATSVVTTAWSGAVKSESNPTLYDSAAPSSYSQAYRSRQFPF
LQVPESPLTGVSSAVIDSDRALSLLSSNAETPEMGLGHTFHHPSPVNPTRPMLSSLHYNS
VPSQYPGSSQGHGMEGHPTGFGLFSDLRSSNSLCQDVFQNDTGASSTSEGHQTLSFSWE

>AAA01884-RA
MDSWKHMGCEIKPMFNGETYPLISNTQALERMDLMDLGFADDIRKSYSISSEPSGEVFSD
EIGTDYPQFGKESNHQQDFPLVDLKLGRLEDGGIDKDSEFSKFNAAESSVRLSSPAKKAR
SSMSSNRSSNCQVLGCNKDLSSFKSYYKRHKVCDVHTKTPKVIVDGIEQRFCQQCSRFHL
LAEFDDTKRSCRRRLSAHNKRRRKPQLASEPGSGIIDFSSSLAFPEVMPSVYLGPSKYKE
GIDNSITSSNFLLQMGCVGEKINKFPANVKDDIAGFAKPTNETLSNIQQTSRALSLLSAQ

SHSISSQLLETASATLTNRGNHADSNPGHSLNHSAGIFNEVSASGPHEPGIYSMEEDGPT
MINPNNSSTVGLHLPNDGISRPGLRNTDCHHPKERGIILNLLQLSSNLQRVEQQKHSTEN
EAG

>AAA13210-RA
MEEVGTQVASPLYIHQNIGGRFCEGALIGAKRSLCYNSGSNHHQQQHQHSQIHRSGHGW
NPKDWEWDSSHFLARSRPLESDRLRLGSLHEVSTPNNKEVVNPVLSSSFTSKSSSPPHDDQ
RGGNSLRLQLGGVGDNNGTGTSFNKTSVGNVQSSNSTEDPVSSSRPNKKVRSGSPGGGNY
PMCQVDSCSEDLSKSKDYHRRHKVCELHSKATKALVGKQMQRFCQQCSRFHPLAEFDEG
KRSCRRRLAGHNKRRRKTQAEDTTSPAIQPTDSHKTGYGNLDIVNLLTVLARGQGNAEQN
VPPCPSLPDKNQLMQILSKINSLPLPTDIATNPHTQPCSTKNGFEQGALGQSNMDVDAASR
STTDLLAVLSATLAASSPGSLAFFSQKSNPGSCVDKNKSTTMDKYTGSDACKKPSIELQS
LGGERSSSSYQSPTEDSDSQVQDTRMNLPLQLFSSSPGDDSSPNLVTSRRYFSSDSSNPT
EERSHSSAAPVTRKLFPLETASESARPVRMSFSEEANVNVEASRTETSASRMTLELFTMG
NKAASNSLQNLPHQAGYTSSSGSDHSPPSFNSDPQKDRTGRIIFKLFDKDPSQLPGALRT
QIYNWLSNSPSDMESYIRPGCVVLSIYVSMSSAAWEQLEESFLQRVEALVQDSDFECWRS
GRFSVNIGRQLAVHKDGRIRVCKPWSTVSSPELFVVSPLAVVSGQDTSLVLRGRNLTSPG
TKIHCAYMGGYSSKEVLKSSDQGLPCEEIRLSEFNVHAAASSVLGRCFIEVENGVRGNCF
PIIIADAKICQELRLLEREFYEAKDSDVITDDQAQYVASPHSHEALHFLNELGWLFQRQI
SSDIAVNDFMLHRLQYLLTFSTERDYSVLVKTLLDIFVEAESRMDGLSTECVEALSNMHL
LHRAVKRSSRKMVDMLVHYSAPCGSDSSKKYIFPPNLRGPGGITPLHLAACTSGSYDIVD
ALTNDPMQIGLHSWRSLLDDGGQSPYSYALMRNNHTLNSMVAQKLSDKRNRQVSVTIGN
EIVESLVPAEVRQRPSLPLDTKQKSCSKCAMRSYSRMPGSHGFLHRPFIHSMLTIAAVCVC
VCLFFKSLHVNSVTPFMWDNVDFGAM

>AAA13530-RA
MMENSGKRVLLTSNGDDVSVNIAYNLAKQGCRLVLMGDEGCLRNIGDRIADSIKDKNAIE
VIGLDMEDDREAVFHGAVEKACNVLGKLDAFVNCYAYEGKMQDPLKMAEDDFKKTLKV
NFMAVWFLVKAVGIKMRDQRTGGSIVLLTSIIGAERGLYKGAAAYGSCLAGVNQLVRIMA
MEIGTHQIRVNGISRGLHIQDEYPTSVGKERAEKLVKDAAPLHRWLDPKKDLASTVSYLISD
GSRYMTGTTVFVDGVCRKRIGVMEWDSKSCAWNGVSKVEFQDNGYHHHLATLAGSSGT
GISNMFSVDLKLGRLGDMGDRGRPRNSGAQSTVICSVDGCASDLNQCREYHRRHKVCER
HSKTPVVLVGGKEQRFCQQCSRFHSLEKFDEVKRSCRKRLDGHNRRRRKRQPETFNMPDS
AGGVLSSQTADGMLQYSCPQMHPVNWPTMSQPDLYFQNNQETIYQPLLNDVAPTGSRRG
GLRVSPDNSGCALSLLSRYSSQSSDIRVGPVMQPTVMSSATAQGSSTSLHLNNSYQLPCSQG
LDDIESSASLSSSSNTNAPNIGGFHTGHAGFRENTRIFPFGWE

>AAA17066-RA
MDLPPLTGGGEESGAPFEWSDLFDFTIDDQLLLNLDVSDQPKEQPPVLLPPVTNAEDNGK
TVAVNGGESGPSDRVRKRDPRMICENFLAGRVPCACPELDALMAEEEEEETGPGKKRPRM
GRTPGVAKCQVPGCEVDIRELKGFHVLSDFDEGKRSCRRKLERHNNRRRRKSADYRGTVE
KEPQVDVQTEDVFSDGEAGKENAWSGGHIAEKEDSKDKTLSNLCSALESQNIESDSNLTF
TPVDKVIDNLEREYSPPSDTKSAYSSALFEDPVSFVQNSVGHGGILSGRGAALVYLNDLS

FRVMREGASVMKVKVAVRAPKLHYVYPPCFEAGKPMDFVACGSNLLQPKFRSLVSFAGK
YLAHDYYVAFPRGKEDKPAIDYDYQFCRIYVPHTEPSYFGPAFVEVENECGLSNFIPILIG
DEHVCSEIKMIHQKYDCSNCRKKLQCIPSGSSYGTCEVSCSRQAALSEFMLDVAWLLKQP
CSEKLNCILTRLLKEYIVQANDFLRQSAYNKELILKQGGECVSNVDTCLGSFPHDDLLSV
DRTACQVTKLRAEMNHTFLEHSDSQGEGESVSLINREVAMNVNDNRDWPKKSCHNIISK
KFTSPRPFIYAIALVAVCFGVCAVVLHPYKVTKFAVTIRRCVFDNSS

>BBB04154-RA
MQIPKTEPDLFTNRPIGLNLGRRTYFSADDDLVSRLYRRSRAVEPALHSPKCQAEGCNAD
LSHAKHYHRRHKVCEFHSKASTVIAAGLTQRFCQQCSRFHLLSEFDNGKRSCRKRLADHN
RRRRKSNNQRITSTAPSMSSGENAQSSPSQTILRSPSDSGMNSSSSVTIAVSPPRVSFDS
SLMQI

>BBB14488-RA
MEAEMGGKADVYSSAVMPVSDLKAVGKKTLEWDLNDWKWDGDLFLATPLNTTCTPVD
CRSIQLFPLGPELATNINVGSEEISKTNEKGKREMEKRRRFVVVPHEEVNEEGRPLNMKLGE
QVYPIAEDEADKLEGKSVKKSKSLGTGTSQPACQVEGCTADLSNAKDYHRRHKVCEVHSK
VSEAFVGNFMQRFCQQCSRFHNLPEFDEGKRSCRRRLAGHNKRRRKTVSETLPNGGSLTD
ENSAGYLLVSLLRILSNLHSNGSDETKDQDLISHLLRNLASQVSGSNLPELQQGYQRILNA
GISVGIPEKNPSSAQELYQAVPSAEAHIGLLTRENQHQKEKTQYASQPGIFHPADDIPGN
GHGSIACAYWPEHLQKSSPPQTSGISDSNSGHSSSSGSDSQGRTDRIVFKLFGKDPDDLP
HQLRTQILDWLSHKPSDIEGYIRPGCIVLTIYLRLNKSLWEELCYDMSSSLSRLLSLSDD
PFWKTGWIYTRVQQSAAFICDGRVVLDTPLPFKSRGSRISSISPIAVPAAQTVQLVVKGS
NLSGPTSRLLCAIEGKYLVQDSCCYSLVDSTAAEQDEVQSLSFHCSIPDVVGRGFIEVED
YGLSGCFFPFIVAEPEICSEICMLERVMEIVGTDEGIDRRNDALEFIHEMGWLLHTNRVS
SMSGQTNIQLDLFSFVRLKWLIDFSMDHDWCAVLRKLLDLLFSGIVDTGHHASVENALSE
MPLLHTAVQRNSRSMVEFLLRYIPRKAKNTKVSDESPSSFLFRPDVTGSNGLTPLHLAAS
CAGFENILDALLEDPGMVGIGAWENARDSTGLTPKDYAYLRGHNHYIDLFQSKVNQNSS
GKHVIVDILGLSNLKSKQKQSDELKSAKLNSLYTEKWQISQNCKLCEQRPSYGFRGTSLTC
RPVVMSLVTIAVVCVCTALFFKSMPRVCYVFVPFRWDTLKYGAV

>BBB14726-RA
MGSNYMTVEGSSTVSSGLSDSINGLKFGQKIYFEDGGKAVVQSGQPPRCQVEGCNTDLSD
AKTYYSRHKVCGMHSKSPIVIVAGIEQRFCQQCSRFHRLPEFDQGKRSCRRRLAGHNERR
RKPPPGSLLSSRLGRLSSSFFGDNTSKNGGFLLDFSSYSRQSEKDLWPGSETSEQVSGSQ
SRSIMWPGHSEDHPSKMYLHGSASESSYSFPSGECITGVSSDSSCALSLLSNQSWGSRNS
SSGTALGNSMNVDGTPVSQCNAAHGVTVAHFPNSSSSWGYKGNNDASCGLHGVPSGHL
RLGQISQPHYNGQFHGDLLMDHVQDGGRQYMDVDHSRAYNSNNTNHVDWSL

>BBB13790-RA
MEAGMGGITDVYSSAVMPVSDPKAVGKKTLEWDLNDWKWDGDLFLATPLNTTHTPLDC
RSKQLFPLGPELAMNNNVGSEEISKTNEKGKREMEKRRRVVVVPHEELNEEGRPLNLKLG
EQVYPIAEDEADKLEGKSVKKSRSAGTGTSQPACQVEGCMANLSNAKDYHRRHKVCEVH

SKVSEAFVGNVMQRFCQQCSRFHALPEFDEGKRSCRRRLAGHNKRRRKTLPETSPNVGSLT
DEKSAGYLLVSLLRILSNLHSNGSDETKDQDLISHLLRNLASQVSGSNLPELQQGSQRLLNA
GISVGIPEKNPSSAQELFQAVPSAEAHIGLLTRENQHQKEKTQYASQPGIFHPADGSIAT
KGSVPRAYLGIDLNNVYDDSQECVDIPGNGHGSIACAYWPEHLQKSSPPQTSGISDSNSG
HSSSSGSDSQGRTDRIVFKLFGKDPDDLPHQLRTQILDWLSHKPSDIEGYIRPGCIVLTI
YLRLNKSLWEELCYDMSSSLSRLLSLSDDPFWKTGWIYTRVQQSAAFICDGRVVLDTPLP
FKSRGSQISSISPIAVPVAQTVQLVVKGSNLSGPTSRLLCAIEGRYLVQDSCYSLVESTA
EQDEVQSLSFHCSIPNVVGRGFIEVEDYGLSGCFFPFIVAEPEICSEICMLERVMETVGT
DEGINRRNDALEFIHEMGWLLHADRLSSMSGQPNIQLDLFPFVRLKWLIDFSMDHDWSAV
LRKLLDLLFSGIVDTGNHASVKNALSEMPLLHTAVQRNSRSMVEFLLRYIPKKVKNTKGS
EQKQSHYEPPISFLFRPDVTGSNGLTPLHLAASCAGFENILDALLEDPGMVGIGAWENAR
DSTGLTPKDYAYLRGHNHYINLFQSKVNKISSGKHVVVDILGLSNLNSKQKQSDELKSAK
FNSLYTEKRQISQNCKLCVQRPNYGFRGTSLTCRPVVMSLVTIAVVCVCTALLFKSMPRV
CYIFVPFRWDSLKYGAV

>BBB16095-RA
MKLGCQAEKCTADLTEAKRYHRRHKVCEHHAKAPSVLVSGIRQRFCQQCSRFHELSEFDD
TKRSCRRRLAGHNERRRKSASESQGEGGSSSSQSKGSNLQLRTGDQAQISYSGNNNNNGN
SSYKQFQIR

>BBB07453-RA
MSAISMMELNAKSPFLWDWENLVMFNTKAAETPKKLQSDSEIEGIEGFEAGSFYSSGGRS
DGGTGSDLGYASSSKSSKSASVDSSADRELKGNQFSLEATDCFQQDLRGKRDLVNSSGTT
RASPTIESSVASGESLIALELGKRTYFEDVSGASNVKNTARSSIPPDSAAKKTKSSSPSIENPRC
QVEGCNLDLSSAKDYHRKHRVCENHSKSPKVVINGLERRFCQQCSRFHGLSEFDQKKRSC
RRRLSDHNARRRKPKPEVIQFNSMRLTSSLYEGRNQLNFAFDQVPMHQTRHGTWDSARES
KVTLAKPGFFTTMKTGGTDQLHSTHSGLPSPTTKVSPVSTQLLPSKGVPSDVFQRGLPVSVA
PSNLAAAQDFRALSLLSTSSWSSCDPEPPALNHSMHTNHASLAPSLPPMHPLPLGGPPISSE
FWQADQPASGGPGSHSSTFQLFKAPNESSFYLY

>BBB14296-RA
MESWWFDSLDKGFESNEAISQSDSIIKGKNVLIGWEHKSPLSNEDSVLTPSQQSVENRSF
SELGIAEVVRRQCPTDSTRNELEDNGSDGDIYSSYVTTNAISGDDESSSKFSSSVMDSSS
RESSLIDLKLGGFGDNPNSSSTRTATAHVLSSADSSTPPKRARVSSQAVHCQVYGCHKDL
SSAKDYHKRHKVCDVHSKTPKVIVNGIEQRFCQQCSRFHLLGEFDDGKRSCRKRLAGHNE
RRRKPQVGFSNRNGRSFQSYTGSNFQGFTPTSTSFICQDILPRGISHTVKYGTNDWVKHI
KVEDGTGCTQTPTYSCINQQLQPKSILPPYDFEKQFPFIDNTNNTGTQFPFGKNVNQHTP
EIVSHSLFQTNSPRNEDLALLDAASTVQELAGISESGCALSLLSFQSQNSSGHSSAMPGC
HPVVIPSSSPQYSVNEVSEKIFGNGAQALTSEVQNRYSSVIVSSTGRNQPSFMSVLNYGN
NAHSEFGNEIHHRSKFMNIKDHLLCEDGTTIDLLQLSSQLQRVENQKLSEPLKQDTENSF
CLRMT

>BBB02950-RA

MGYKLTKNYCDLAELKKGGIATISAIFGSSSFDTEKAIGERLVDLKLGRLGDIGKGLVNNFK
GHSHSIMDPFSPTAGSSKRPRTPGTSSQVVSCLVDGCKADLSKCRDYHRRHKVCEMHSKTP
RVTIGGNEQRFCQQCSRFHSLGEFDEGKRSCRKRLEGHNRRRRKPQPEPLPVNHGNFFSAS
QGSRFLAFSNQPIIPATSVMSTAWSTVKSESSPTLYNSATPSSYSPAYRGRQFPFLQVPESPLTG
VSSAVIDSDRALSLLSSNAETPEMGLGRTFHHPSPINPTRPMLSSLHFNSVPSQYSGSSQGHG
MEGHPTGSGLFSDLRSSNSLCQDVFQNDTGASSTSGGHQTLSFSWE

>BBB13189-RA
MEEVGTQVASPLYIHQNIGGRFCEGALIGSKRSLCYNSGSNHHQQQQHSQIHRSGHGW
NPKDWEWDSSHFLARSRPLESDRLRLGSLHEVSTPNNKEVVNPVLSSSFTSKSSSPPHDDQ
QSGNSLRLQLGGVVDNNGTGTSFNKTSVGNVQSSNSTEDPVSSSRPNKKVRSGSPGGGNY
PMCQVDSCSEDLSKSKDYHRRHKVCELHSKATKALVGKQMQRFCQQCSRFHPLAEFDEG
KRSCRRRLAGHNKRRRKTQAEDTTSPAIQPTDSHKTGYGNLDIVNLLTVLARGQGNAEQN
VPPCPSLPDKNQLMQILSKINSLPLPTDIAANPHIPPCSTKNGFEQGALGQSNMDVDAASR
STTDLLAVLSATLAASSPDSLAFFSQKSNPASCVDKNKSTDMDKDTGSDACKKPSIELQS
LGGERSSSSYQSPTEDSDSQVQDTRTNLPLQLFSSSPGDDSSPNLVTSRRYFSSDSSNPT
EERSHSSAAPVTRKLFPLETASESARPVRMSFSEEANVNVEASRTETSASRMTLELFTMG
NKAASNSLQNLPHQAGYTSSSGSDHSPPSFNSDPQKDRTGRIIFKLFDKDPSQLPGALRT
QIYNWLSNSPSDMESYIRPGCVVLSIYVSMSSAAWEQLEESFLQRVDALVQDSDFEFWRS
GRFSVNIGRQLAVHKDGRIRICKPWSTVSSPELFVVSPLAVVSGQDTSLVLRGRNLTSPG
TKIHCAYMGGYSSKEVLKSSDQGLPCEEIRLSEFNVHAAADSVLGRCFIEVENGVRGNCF
PIIIADAKICQELRLLEREFCEAKDSDVITDDQAQYVASPHSHEALHFLNELGWLFQRQK
SSDIAVNDFMLHRFQYLLTFSTERDYSALVKTLLDIFVEAESTMDGLSTECVEALSNMHL
LHRAVKRSSRKMVDMLVHYSAPCGSDSSKKYIFPPNLRGPGGITPLHLAACTSGSYDIVD
ALTNDPMQIGLHSWRSLLDDGGQSPYSYALMRNNHTLNSMVAQKLSDRRNRQVSVTIGN
EIVESMVPAEVRQRPSLPLDTKQKSCSKCAMRSYSRMPGSHGFLHRPFIHSMLTIAAVCVC
VCLFFKSLHVNSVTPFMWDNVDFGAM

>BBB04921-RA
MDSWKHMGCEIKPMFNVESYPLISNTQALERMDFMDLGFPDDIRKSYSISSEPSGEVFSD
EIGTDYPQFGEESNHQQDFSLVDLKLGRLEDDGIDKDSELSKINAAESSVMLSSPAKKAR
SSMSSNRSSNCQVLGCNKDLSSFKSYYKRHKVCDVHTKTPKVIVDGIEQRFCQQCSRFHQ
LAEFDDIKRSCRRRLSAHNKRRRKPQLASEPGSGIIDFSSSLAFPEVMPSVYLGPSKYEE
GIDNSIKSSNFLLQMGCVGEKINKFPANVKDDIAGFAKPTNETLSSIQQTSRALSLLSAQ
SHSISSQLLETASATLTNRGNHADSNPGHSLNHSAGIFNKVSASGPHEPGIYSMDADEDG
PTMTNHNSSNTVGLHLPSDGISRPGLRNTDCHHPKERGIILNLLQLSSNLQRVEQQKHST
ENEAG

>BBB12016-RA
MEWDSKSCAWNGVSKVEFQDNGYHHHLATLAGSSGTGISNMFSVDLKLGRLGDMGDVS
MDTLKNSMPLNMASSSSPSPVLALSKRGRPRNSGAQSTVICSVDGCASDLNQCREYHRRH
KVCERHSKTPVVLVGGKEQRFCQQCSRFHSLEEFDEVKRSCRKRLDGHNRRRRKRQPETFY
MPDSAGGVLSSHTADGMLQYSCPQMHTVNWPTMSQQPDLYFQNNQETIYQPLLNDVAP

TGSRRGGLKVSPDNSGCALSLLSRYSSQSSDIRVGPVMQPTVMSSATAQGSSTSLHLNNSYH
QLPCSQGLDDIESSASLSSSSNTNAPNIGGFHTGHAGYRENSYRIFPFGWE

>BBB08709-RA
MDLPPLTGGGEESGAPFEWSDLFDFTIDDQLLLNLDVSDHPKEQPPVLLPPVTNAEDNGK
TVAVNGGETGSSDRVRKRDPRMICENFLAGRVPCACPELDALMAEEEEEEAGPGKKRPRM
GRIPGVAKCQVPGCEVDIRELKGYHRRHRVCLVCANATSVVIEGESKRYCQQCGKFHLLS
DFDEGKRSCRRKLERHNNRRRRKSADYRGTVEKEPQGDVQTEDVFSDGEAGKENAWSGG
QIEKEDSKDKTLSNLCSVLESQNIDSDSNLTFTPVDKVVDNLEREYSPPSDTKSAYTSACP
TGRISFKLYDWNPAEFPRRLRHQIFQWLASMPVELEGYIRPGCTILTVFVSMPQYMWVKL
FEDPVSYVQNSVGHGGILSGRGAALVYLNDLSFRVMREGASVMKVKVAVRAPRLHYIYPP
CFEAGKPMDFVACGSNLLQPKFRLFPNPDDLKKVYKEYGAFACGLDYLSQIHVMNARNE
EELLSWWANYGASTPLLQGLALKLISQPASSSCCERNRSTYGTIQTTKRNRLTSSRVEDLV
YVHMNLRLLARKKNEYREGPSSYWDIGDVMDVDHILELVELSLNEPEIEAMTLEVENECG
LSNFIPILIGDAHVCSEIKMIHQKYDCSNCRKKSQCMPSGSSYGTCEVSCSRQAALSEFM
LDVAWLLKQPCSEKLHCILTSSQIQRYNCLIKFLISNKSTSILEKILQSLQIVFDGMNSCDVIN
NDTDRDRRLLKEYIAQANDFLRQSAHNKELILKQGGECVSNVDPCLGSFSRDDMLSVDHT
ACQVTKLRAEMNRTFLEHSDSQGEGESISLINREVAMNVNDSRDWPKKSCHSVISKKFTST
RPFIYAIAVVAVCFGVCAVVLHPYKVTKFAVTIRRLAVLYANNNKSLSKSKHIDVKFRAVK
ERVESKQLSLEHINTNSMITDPLTKGLPTKVFHEHTARMVVMPPKDI

Supplementary File S6

39 sequences of AtSPL7 orthologs from different species

>AtSPL7
MSSLSQSPPPPEMDIQPPALVNDDPSTYSSALWDWGDLLDFAADERLLVDQIHFPPVLSPPL
PPLIPTQTPAESELDPSPEESGSGSDRVRKRDPRLICSNFIEGMLPCSCPELDQKLEDAELPKK
KRVRGGSGVARCQVPDCEADISELKGYHKRHRVCLRCATASFVVLDGENKRYCQQCGKFH
LLPDFDEGKRSCRRKLERHNNRRKRKPVDKGGVAAEQQQVLSQNDNSVIDVEDGKDITCS
SDQRAEEEPSLIFEDRHITTQGSVPFTRSINADNFVSVTGSGEAQPDEGMNDTKFERSPSNG
DNKSAYSTVCPTGRISFKLYDWNPAEFPRRLRHQIFQWLANMPVELEGYIRPGCTILTVFIA
MPEIMWAKLSKDPVAYLDEFILKPGKMLFGRGSMTVYLNNMIFRLIKGGTTLKRVDVKLES
PKLQFVYPTCFEAGKPIELVVCGQNLLQPKCRFLVSFSGKYLPHNYSVVPAPDQDGKRSCN
NKFYKINIVNSDPSLFGPAFVEVENESGLSNFIPLIIGDAAVCSEMKLIEQKFNATLFPEGQEV
TACSSLTCCCRDFGERQSTFSGLLLDIAWSVKVPSAERTEQPVNRCQIKRYNRVLNYLIQNN
SASILGNVLHNLETLVKKMEPDSLVHCTCDCDVRLLHENMDLASDIHRKHQSPIESKVNPP
SSGCCCVSSQKDIPSRILNFNKDPEAGLDCKERIQADCSPDSGGKETDPLLNKEVVMNVND
IGDWPRKSCIKTHSALAFRSRQTMFLIATFAVCFAVCAVLYHPNKVTQLAVAIRMRLVHKI

>CRR1
MASDLANIFNDEEAGSDRANSITSSDLDPFLLQGDDQTWAVEDWNWDPFNMFAAPKETP
NVACCQALKRRKVTEQSATSAPQQAAQQHHQHAACQPCHGASGSRAGPPAAPTAACCS
MAPGVQQHAPAPAPAQQLTGPLQTALNSMMPGQQQQRVADACGVSNMCHAPGMVAPP
PPMPVAAPLAATGHQAGGMGMGHMNPAMGHMGWPAMPDMSNPAAAAAAAAAAS

FFPTMGAGGRGPLATMGWGAQPMALQMPSMGLPGFPGGHTQCAPALQMPAVAFPTPSS
GIFHQQASMAVPPSALPSPALGCSGDTTSLQGSADNCAKPPCCSGNRATGPTCSGGRQQA
DSSNKAAPPPPKQPAQNTVGARGGAAAPAPGDLSDFNISDSEDDDEVARRPSAAGTQRGG
VGNAAALNAAAYANLGDIVSLQDVGDDTGAMVCQVPGCGKDLTNLKEYHQRYRICDVH
IKLQQVLKDGRLQRFCQQCGRFHDLTAFDGNRKSCRDQLSKHNARRRRRAQAEQAKGRA
AAQEAAVAAAGAMAAAAAVGGAAGGAPAVPGFEGGDVGKLLACLMQNPTQLHALRLL
LGVPTHPALPAAAPAAGLGMAAGGDTAAQPDQARTYGLARDILAGRNEFSPAFESEHRMI
RLSMKLFNKTPADLPHDLRNQVTSWLASAPLAMEASIRPGCVFLTVQMLVDEAGAQQAA
APGALHGLVEHLLTRTGCPFWHMGMYTVQMGTDMLLVRDGKMAGDANVNASGAAAP
AATGEGAEAAARVRGDGRFPVVRRLGPLAAVAGQPTVLKLHGLNLDAPGCSVILRWGNK
HLKAHMQALSTHRAIVRLPPLPDLCGPVWVEVARGAYLSPAKQLLVARNEELVQEINKLD
VKTGPLRHDTVELLLQDLALVLQHIAAQPGVAAQLQHAVIALKARRLLAMACDMGWAA
VASAVLPLACARCSCASEMVAAIHSASTPSQTGAADKRGLTLLHRAVRSGSVSLLAGMLAW
GDSHGYRWRVDAEGPAGITPLHLSAMLDDARIGLLLLDHCGWPAAFTHLRSDGGVTPFH
LAFQMGHYQVDALMSALGGLHQVADPNNAAAINNAAAAAAAAMSPSRRSRCGNAAA
MGCKPDPDEDVKPDLAALQRGGRNGGGGSGGCTELDPCENCHCTLPPLLLSIMASCTDC
GRRRMCMETECNNAAQRCGDCGCGGARPTARVDSGEVCSSRQHGTIFSITALCQGCHAN
RTLAVA

>XP_002948544.1[Volvox carteri f. nagariensis]
MDLANLFNDEGAGSERTNSITSSDLDPFLLQGDDQTWAVEDWNWDPFNMFAAPKETPN
VACCQALKRRKVDNTAAAQGQPQQQAQQGACCQGPSGSIRAGGQPPAAAAACCSFTPN
QTQAMQQLTGPLQAAMNSLAPQKAVVETCAVSSMCHGPGAMVPPAAVPAAASVAATG
PQMLNVNMGMALPAGMGWAGLGDMSSPAASAAAAAMAAAVDMPGFFTSMTGGTGR
PLAGMGWGIQPMALQMPSMPMQPFPGSHAQCAPALSMPASGFPTPSGVFQQQASMALP
PSSVPSPALGSSGETSSLQGSADNAKALCCSGNRGGGGPTCSGGRQQADSSNKASMPLPN
KAHQTGATGGRGADGAMVGGDLSDFAISDSEDEDEVGRRGPLGNNRGNGAHHQQHQY
LGDVIALHDVGDDNGLMVCQVPGCGKDLSGLKEYHQRYRICDVHIKLQQVMKDGRLQR
FCQQCGRFHELTAFDGNRKSCRDQLSKHNARRRRRAQAEQAKGKSAAVDPFAVGAVGC
GAAAAANGGATASFDGDVGKLLTCLMQNPSQLHALRLLLGVPTHPALPASHPAPGLGLG
VGLGSGSPADSSSDGSDQARTYALARDIMGARNEFAPAFESEHRLIRLSMKLFNRTPADLPS
DLRNQVNSWLASAPAGMEASIRPGCVFLTVQMLVDEAGEAQAMAPGALGSLAQHLLTR
TGCPFWHTGMYTLQLHEDIMLVRDGRVSCDAASANGDGRFPVIRRLGPLAAVAGQPVTL
KLHGQHLDAPNCLVIVRWGAQHLKAHMQPLSAHRAIVRLPPLPELCGPVWVELMRGAY
LSPAKQMLVARNEALVEEINKLDVRYGPLSQETVELMLQDMAIVLQHIAGEQGAGAQLP
HAAIALKARRLLAIACDMGWAAVASAVLPLACARCSCASEMVAAIHAASVPSSASSASSSS
GDKRGLTLLHRAVRSGSIPLLAGMLAWGDSHGYRWRVDAAGPAGITPLHLSAMLDDARV
GLLLLDHCGWPAAFTHLKSDNGVTPFHLAFQMGHYQVDSLMAILGGLHQIDQHAGANN
AVGAGAQPAGRRSRCGANAAAMVMGCKAEENEDVKPALRGHGHNNTATAAKNGCCA
ELDPCEMCHCTLPPLLLSIMASCNECGRRRMCMEAECTGAADRCGGCCCGTARPTLRSDS
GEVCSSRQHSTVFSITALCQGCHGNRILAVA

>OsSPL9

MDAPGGGGGGGGGGGGVDAGEPVWDWGNLLDFAVHDDDSLVLPWGDDSIGIEADPAE
AALLPPAPSPQPAEAEAEAAGPASLPSSMQAEGSKRRVRKRDPRLVCPNYLAGRVPCACP
EIDEMAAALEVEDVATELLAGARKKPKGAGRGSGAAVGGSGGGASRGTPAEMKCQVPG
CEADIRELKGYHRRHRVCLRCAHAAAVMLDGVQKRYCQQCGKFHILLDFDEDKRSCRRK
LERHNKRRRRKPDSKGILEKDIDDQLDFSADGSGDGELREENIDVTTSETLETVLSNKVLDR
ETPVGSDDVLSSPTCAQPSLQIDQSKSLVTFAASVEACLGTKQENTKLTNSPVHDTKSTYSSS
CPTGRVSFKLYDWNPAEFPRRLRHQIFEWLSSMPVELEGYIRPGCTILTVFVAMPQHMWDK
LSEDTGNLVKSLVNAPNSLLLGKGAFFIHVNNMIFQVLKDGATLTSTRLEVQSPRIHYVHPS
WFEAGKPIDLILCGSSLDQPKFRSLVSFDGLYLKHDCRRILSHETFDCIGSGEHILDSQHEIFRI
NITTSKLDTHGPAFVEVENMFGLSNFVPILVGSKHLCSELEQIHDALCGSSDISSDPCELRGL
RQTAMLGFLIDIGWLIRKPSIDEFQNLLSLANIQRWICMMKFLIQNDFINVLEIIVNSLDNIIG
SELLSNLEKGRLENHVTEFLGYVSEARNIVDNRPKYDKQRQVDTRWAGDYAPNQPKLGIS
VPLAESTGTSGEHDLHSTNAASGEEENMPLVTKALPHRQCCHPETSARWLNAASIGAFPG
GAMRMRLATTVVIGAVVCFAACVVLFHPHRVGVLAAPVKRYLSRNYSS*

>P.patens|Pp3c3_31330V3.3
MGHLDDSVAGWEWDSVLLLANPSLDTSPLQSSLQAHSSLDYITDFAVGQSWAGLGSLQTS
SPKPPDGKVAAGPQSSSSAEKTTTSDYELFRDPRLDCPNFLAGRVPCACTDEDDDEGGSRS
KRAKVSARCQVPACGADLAGLKGYHQRHRVCLQCANSTTVILRDIPHRYCQQCGKFHVL
SDFDEGKRSCRFKLERHNNRRRRKVQESGEDGATPGGGSDKASLNSGGGRVNGDENSAV
ESKAHAEVEQSVVVQVSKEASSPVEQSDTTDTTQILSAGIVDGSGSRTDGAQEALSAPRSKE
YTMPGPNVSGIAESAPLWNSTETPGGVSNGHKSGVDEDSLLALLLEDSPNVDERLPSRGFES
TSPIPPRSSKKPSAYASSHPTARISFKLYDWNPGDFPRNLRQQILEWLSNMPVDLEGYIRSGC
TILTLFISMPQSMWEGLNADWEGAVARLVCNPQNTSGFWEKGYFKAKLGRQTVHFENGK
AVNRSGGKEDCMPCMPVLQSVEPVCFRAGTGCMLSITGRNLLQANTKLLMSHGGKYIKA
WVLKASSEGKQEDKWQIVVPPVDECQAGPVFIEVENEDRLSNIMVVLVGDAEFCNEVESM
ELYTSSCEAQDLLFDLGWMLKESTLDSGVLIQRLRSLLDYANAKGWERVAERILEAADRRG
VLRKLVGAADARSFDWRNIWAESTGHSHSTYKLSSFAVPVSSRSYSMPSLAQSKRDADEHT
HIVVCNSSSKDLTTTSLLPQRGMREKRRSPWGRNTYRLVFAVASATAACAGLCVVLQHPE
QVAQLSTSLRRCLWSN*

>RcSPL7
MEPSPPSPPPSAIARRPKKHDEMEIHASVTEDPTSSALWDWGDLLDFTVDDQFPISFDSIDTT
VSSEVYDNNNETNNHNPVIESTTRAVVQDRVRKRDPRLTCSNFLAGRVPCACPELDEKLLE
EEESLPGKKRVRTTRSSSGITRCQVPGCEVDISELKGYHKRHRVCLRCATAGSVLLDGHRKR
YCQQCGKFHLLPDFDEGKRSCRRKLERHNDRRRRKPHDSKGTAVDKEIQGELQSEETACE
AEAGKDGQIIEKEAAVVESEDGNVSALHSDPNSQNLNSDSGLSVGTPKRGGKDDTKFSFSP
SNCDNKSSYSSLCPTGRISFKLYDWNPAEFPRRLRHQIFEWLASMPVELEGYIRPGCTILTAF
LAMPTFMWAKLFEDPMSYVHDLVIIPGKMLSKRGPMLIYLNNMIFHVMKDGNSVMKVNI
EGRAPRLHYVHPTCFEAGKPIEFVACGSNLLQPKFRLLVSFSGKYLAYDYCVALPHGHTEG
CSGLDHQLCKIFIPHIEPNVFGPAFIEVENESGVSNFIPVLIGDREICSEMKIIQQRFDASHLPK
GSQCEVSAQRQMAFSELLVDIAWLLKKPSSESSQRIMSSSQIQRLNSLLNFLLLHEATAILDK
ALKNLKIILMETEREVSGSSDADMKLLQKHVDWAWNILYQKVKKRDGLLLQWECTIQGRS

SGKCSDGDGPSVAPFTSEDLEKSSTGKLGLIANTSDFVRSDKVPLLNKEVVMNVNLVKDRP
NQSCSLIFSKRVLRSRPTVFLIATVAVCFGVCAIILHPNQVSRFAVSVRRCLTDRF

>B.distachyon|Bradi2g25580.4
MDASDSGGASAAPDAGEPDWDWNHILEFAVRGDDSLILPWDDTLGTAEAGPAEGAFLPA
PSPALPVEAEPVAPPPPVEAGGSRSGVRKRDPRLVCPNYLAGIVPCACPELDEMAAAAEAE
EVASEMLAGPRKKSRPASRGNGVAAGGGGGGSGVAGRGGAVEMKCQVPGCEADIRELKG
YHKRHRVCLRCAHATAVMLDGVQQRYCQQCGKFHVLLDFDEDKRSCRRKLERHNKRRR
RKPDSKGAFEKEVDEQLDLSADGSGGCELREENTDGTTCEMVETVLSNKVLDRETPVGSED
VLSAPTCTQPSLQNEQSKSVVTFAASVEGCLGTEQENANITNSSMHDTKSVYSSSCPTGRISF
KLYDWNPAEFPRRLRNQIFEWLSSMPVELEGYIRPGCTILTVFIAMPQHMWDQLSEDAANL
VRDLVNAPSSLLLGKGAFFVHVNNMIFQVLKDGATLMSTRLEVQAPRIHYVHPTWFEAGK
PVELLLCGSSLDHPKFRSLLSFDGEYLKHDCCRLTSHETIACVKNAAALDSQHEIFRINITQT
KADTHGPGFVEVENMIGLSNFVPVLFGSKQLCSELERIQDALCGSNEKYKSVFGEVPGATS
DLCGRLELKQTAMSGFLIEIGWLIRKSSPDELKNLLSSANIKRWTSVLKFLIQNDFINVLEIIV
KSSDNIIGSEILSNLERGRLEHHVTTFLGYVRHARNIVEDRAKYDKQTQLETRWCGDSASN
QPNLGTSVPFAKEVSAIMLQANIVVIIIKIIVRCLKSLQ*

>Z.mays|Zm00001d010309_P004
MASPTCCDWGRQDQFHSLHPATASPSLPASPRGIVRVPKSPELILPCGRAVVRASLSPEMDA
PDSGGGGSADAGEPIWDWGNLLDFVVQDDDSLILPWDDAAGIAASDPTEAATALLPASPP
LPQSVEEEPEPEPEPGPVLPPPPLRVQGIGRRVRKRDPRLVCPNYLAGIVPCACPEVDEMVA
AAEVEDVATEFLAGARKKTKTAGRRGKAEAAGTAGGTVRAAVAEMKCQVPGCEADIREL
KGYHRRHRVCLRCAHAATVMLDGVQKRYCQQCGKFHVLLDFDEDKRSCRRKLERHNKR
RRRKPDSKGTLDKEIDEPLDMPADVSGSDELREENMEGITSEILETVLSNKVMDRETPVGSE
DVLSSPTCTQPCLQNDQSKSVVTFAASVEACIGAKQESIKLANSPMHDTKSAYSSSCPTGRIS
FKLYDWNPAEFPRRLRHQIFEWLGSMPVELEGYIRPGCIILTVFIAMPQHMWDKLSDDAAD
LLRNLVNSPNSLLLGKGAFFIHVNNMLFQVLKDETTLMSTRLDIQAPRIDYVHPTWFEAGK
PVNLILYGSSLDQPNFRSLLSFGGDYLKHDCYRLPSHDTFDGFESGDFIPDSQHEIFRIHITQS
RPDIYGPAFVEVENMFGLSNFVPILFGSKQLCSELERIHDALCGSYSENNVLGELLSASSDPH
EHRKLCSSVMSGFLIDIGWLIRKPTPDEFKSVLSSTNIQRWICMLKFLIQNDFINVLEIIVKSM
DSIMGSEVLSNLERGRLEDHVTAFLGYVSHARNIADRRANHDEKTQIETRGIIVTSPNQPNL
GASVPFASENTDFGGDNKLNSTDEEEIMPLVTRDVSHRHCCQPDTTVRWLKPSLIVTYPGG
ATRMRLVTTVAVAAVLCFTACLVLFHPHGVGVLAAPVKRYLSSDSAS*

>Sorghum bicolor|Sobic.009G135000.1
MDVPDSGGGAPDAGEPIWDWGNLLDFVVQDDDSLVLPWDDAAAGIAAAADPTEAATAP
LLPASPPLPQPVEVEPEPEPEPEPEPGPVLPPPPLRVQGIGRRVRKRDPRLVCPNYLAGRVPC
ACPEVDEMVAAAEVEDVATEFLAGARKKTKTAAAAARRSKAAAAGAAPGAAGGGAAR
AAAAEMKCQVPGCEADIRELKGYHRRHRVCLRCAHAAAVMLDGVQKRYCQQCGKFHIL
LDFDEDKRSCRRKLERHNKRRRRKPDSKGTLDKEIDEQLDLSADVSGDGELREENMEGTTS
EMLETVLSNKVLDRETPVGSEDVLSSPTCTQPSLQNDQSKSVVTFAASVEACIGAKQESVKL
VNSPMHDTKSAYSSSCPTGRISFKLYDWNPAEFPRRLRHQIFEWLASMPVELEGYIRPGCTIL
TVFIAMPQHMWDKLSDDAADLLRNLVNSPNSLLLGKGAFFIHVNNMLFQVLKDGATLMS

TRLDVQAPRIDYVHPTWFEAGKPGDLILYGSSLDQPNFRSLLSFDGDYLKHDCYRLTSHDTF
DRVENGDLIPDSQHEIFRINITQSRPDIHGPAFVEVENIFGLSNFVPILFGSKQLCSELERIQDA
LCGSYSKNNVLGELLSASSDPHERRKLHSSVMSGFLIDIGWLIRKTTPDEFKNVLSSTNIQRW
IHILKFLIQNDFINVLEIIVKSMDTIIGSEILSNLERGRLEDHVTTFLGYVSHARNIVERRANH
DEKTQIETGGIIVNSPNQPSLGASLPHASENTDIGGDNKLNSADEEETMPLVTRDVSHRHC
CQPDMTARWLKPSLIVTYPGGATRMRLVTTVVVAAVLCFTACLVLFHPHGVGVLAAPVKR
YLSSDSAS*

>H.vulgare_Morex V3|HORVU.MOREX.r3.1HG0062640.1
MDASDSGGGGAAADAGEPVWDWDNLLDFAIPDDDSLLLPWDDPLGIEGDPTTETALLPA
PPPPQPVEPAPAPPSPSVEAGASRRGVRKRDPRLVCPNYLAGIVPCACPELDEMAAAAEVE
EVAAEVLAGPRKKPKAASRGSSGAVVKTGGGGSGSGVAARGGAAEMRCQVPGCEADIRE
LKGYHKRHRVCLRCAHASAVMLDGVQKRYCQQCGKFHILLDFDEDKRSCRRKLERHNKR
RRRKPDSKGSLEKEIDEQLDLSADGSGGGELREENIDGVARDMLETVLSNKVLDRATPVGS
EDAVSSPTCTQLSLQNDQSKSIVTFAASDEAAKQENAKLTNSPVHDSKSAYSSSCPTGRISFK
LYDWNPAEFPRRLRNQIFEWLSSMPVELEGYIRPGCTILTVFIAMPQHMWDKLSEDTANLV
RNLVNAPSSLLLDKGAFFVHVNNTIFQVFKDGATLMSTRLEVQAPRIHCVHPTWFEAGKPI
ELLLCGSSLDQPKFRSLLSFDGEYLKHDCCRLTSYETFGRVKSGDPTFDSQQEVFRINITQKK
LDTHGPGFVEVENVFGLSNFVPILFGSKQLCFELERIQDALCGSSKYKSANGELPGVTSNPC
ELWELQQTAMSGFLIEIGWLLKKPSPDEFKNLLSKTNIKRWICVLKFLIQNNFINVLEMILMS
SDNIIGSEVLSNLEKGRLEHHVTAFLGYIRHARNIVDNRAKHNEETQLGTRWCGGSASDQP
SLGTSVSLGKENVAASDESCFPSTNECEEEESVPLVTNEAVSHRQCCQPEMNARCLSPALVA
PFPGGAMRMRLITTVAVAAVLCFTACVAVFHPDRVGVLAAPVKRYLFSDCPPWNHSGC*

>T.aestivum v2.2|Traes_1BL_8709BB152.2
MAAAAEVEEVAAEVLAGPRKKPRAGSRGSSGAVVKIGGAGGGSGVAGRGGAAEMRCQV
PGCEADIRELKGYHKRHRVCLRCAHASAVMIDGAQKRYCQQCGKFHILLDFDEDKRSCRR
KLERHNKRRRRKPDSKGTLEKEIDEQLDLSADGSGSGEIREENIDGASCDMLETVLSNKVLD
RETPVGSEDALSSPTCTQLSLQNDRSKSIVTFAASAEACLGAKQENAKLTTSPVHDSRSAYSS
SCPTGRISFKLYDWNPAEFPRRLRNQIFEWLSSMPVELEGYIRPGCTILTVFIAMPQHMWDK
LSEDTANLVRNLVNAPSSLLLGKGAFFVHVNNTIFQVLKDGATLMSTRLEVQAPRIHCVHP
TWFEAGKPIELLLCGSSLDQPKFRSLLSFDGEYLKHDCCRLTSREIFGCVKNGAPTFDSHHE
VFRINITQTKPDTHGPGFVEVENVFGLSNFVPILFGSKQLCFELERIQDVLCGSSKYKSTNGE
FPGITSDRCEHWKLQQTAMSGFLIEIGWLIKKPSPDEFKNLLSKTNIKRWICLLEFLIQNNFIN
VLEMIVKSSDNIIGSEILSNLESGRLEDHVTAFLGYVRHARTIVDQRAKHNEETQLQTRWCG
DSVSDQPSLGTSVPLGKENVAASDDFCLPSTNAECEAEESVPLVTNEAVSHRQCRAPEMNA
RWLNPALVAPFPSGMMRTRLVATVAVAAILCFTACVALFHPGRVGVLAAPVKRYLFSDCPP
WHHSGC*

>PTQ47244.1[Marchantia polymorpha]
MAHGHETGLAWEWDSVLLLANPSLTSHSLEGDGSTSCGLPGSSAADQNHEHDNHCHEH
NHEHNHDKFHAHETSSADHVQTDSISSGSQQMGVNTDWRDPRLDCPNFLAGRVPCACT
DNDDDDSGVSRKRSKPVPRCQVQSCGAELTNLKGYHQRHRVCLRCAHATRVVLRNQPHR
YCQQCGKFHPICDFDEGKRSCRRKLERHNNRRRRKALESEDTLPPLDSGDIEPRSDSSPADD

AMNTTKPILFLEGEDKASSGGGVGFDSEMAQPSAVVTPNGKFIASKEADEAVIATVGTDVE
MSLQPLAPPVDKIVEEIFPTSEPSTKSPELVHSPEVQHVHKKDCLPHPEHVPISRDSHLSNTQ
TMSTNDMSSHHISPRDPQEAERLVRGHDEESLLALLMEDNSATEGSGLSQNGSVEVSEALL
WDSSPPPIRPSKQATYSSPNPTGRISFKLYDWNPGDFPRQLRQQVMQWLSQMPVDLEGYIR
SGCTILTLFVSMPQPMWEKLNADWTESVLRLVRGQCGTFKFWNSGYLIARVGQQIVHVEN
GKAVHPLCGRSDQGPMLEGVYPSCLEAGVEQVVVISGSNLQQSESRLLVSFDGSYIKNEVSS
PGMDKEKADSSSLLQATFPALHSQKYGTAFIEVEHTTGISNFIPLLVAEKKIVDEVRTLELEID
TATTSCCSSQSHDSRFQPLNQKRYHDLLVDLGWVLRNYTLNNFNPHHQSLGQDVERLQGL
LLFGVRRGWYTTVQRVLQVAKRSGVIERIGFLKNGMTALHVALLHQQFAMMDHLTSLMA
SLACAGSCASNSWWDVDCPVSRGLTARQVLTLQAKRMHYGSAKPEGRKLFGKSLAYEIRH
VSQPNVNPDERRFSKGCNSKADICPKNLGYRLLSKESPDYHEVDVLCSTEEEEARFFFPSVD
HSRGKISTWNSLLRRDDVDTQVKSLVPYGRSRCSVEGLTMYISRASTRRPVYASTVGVLLVC
AGLCVMLQHPNEVIELSLSLRRCLWGLQNGPV

>XP_002991675.2[Selaginella moellendorffii]
MGHDDDPNSSSSSLWDWDSVLLLAHPSDKGGGHGQEAEQGGAEAPGHSHQQQQQQQ
EQQQRRRRDPRLSCPNFLAGRVPCACSDEEDEEEAERSKKRSKLSSARCQVPACHADLTAL
KGYHQRHRVCLTCANATSVWVRGQHQRYCQQCGKFHPLGDFDEGKRSCRRKLERHNDR
RRRKLPESGDTSVVSDKHGTAPNTTTNSDVSDVQQGVAGGGGAPSANDLYCLRSHAQRLL
QGSKPPYHEGDDDSFGVDTNVASGEESLLAMLLEEQQEQHNNQSVSTTSGFPKRTTYTSPC
PTRRISFKFYDWNPADFPRRLRGQIMEWLANMPVELEGYIRSGCTILTLFIAMPQSLWAKLE
EDWNDYLVKLTTGAETGFWDKGFVLAFLGNQVAYIHNGSVSSPSLCERATKKYPVIEAVFP
LCVEAGTEATVTLTGHNLLKLNSRVLVASRGKYLPCCIRASSHFYTSIQIDVLVPQGPGVAFV
EIGYDHGISNSVPLLVADKQLCEEVQTLEQDLKASSSGCCSGRDVCRLETHEVLVDLGWVIS
HTSRAKRVSATPLHVCRLQRLLVFAVERGWTAATKRILEAARQMGQSDGVGIEKALQAAV
AHNRADIIKVLLLYQGKREASLYEGFADGRAAATLGRLCSTRLLHSSSLRYKRRTIVKVAVG
LAVACAGICLVVQHPRQVFHISTSLRRCLWYKP

>MBC9848774.1[Adiantum capillus-veneris]
MADCLHVLEEWDWENIFTSSLLEHDLPWHGELSEPVSMKAAATPSHASIHVSVPSSTIPVN
FTNSQLSVKSGKEADDLGTPSDVIATNTSDGSLDSSGYNHDTAVSSHHMPSFETSLINPPAA
RYSKKKKRDPRLDCPNFLAGRIPCSCPEEDELEADIEVEPLVKKRSKVGARCQVPTCGEDIG
HLKGYHQRHRVCLECANSPKVMLKDLPHRYCQQCGKFHRLSDFDEGKRSCRRKLERHNR
RRRRRLVDEEGEAQDCEARSLGIDIDNSNLQEGAAILNAQTVVTSTKRSQGMTRAKQKTK
GESIFQDVTPLLPTDDQCANDDSSGSVQRFEEDINSSNKRNLDEHCPLNVLLENGNEHIEE
GSRSLKHTLKVVPGTSYASIGQWKHTDYVSPCPTGRISFKLYDWNPADFPRRLRQQIFDWL
ANMPVELESYIRSGCIILTFFVTMPQVMWDKVLAEWKGQVQTFILKSGVKLVGTGQLSMPL
VEKSLVLKDGKATSAFCKDLLAPFVVDLYPRSLEAGCHTQIYVFGFNFSGGRFLLSFGEQYV
ECSDWESVDRSHFTAHKIWNSLGNIEVQKVNVFLPDSETFGPAFAEVESVHGLSNFVPVLV
ADKSICSEINTVRLNYISSASSNICVKSLGKDVKALRSQFGMDILTDLGWALKYITRPLPVDK
FELREALIDRLQTLHLFSLEHRCHAVARLVQTAVGNFIQGGNVRNTPEMLKGMKALNISGR
LHFSKGTAVKGQLTPRSAADDDSIQAVDKPNKIKGLQGPKCAQQQVHLTRRSELAEGGHD
SKMDILPIEEPLLEERISISEEGWNLKACWALSSSWQRKPATGTSTLCTSNKKVTRILVTAVAV
VTVCTGMCFMLQHPHEVAEMSMSLRRCLWGSNDLTHV

>KAH7306849.1[Ceratopteris richardii]
MADSNYVLESWDLETLFASSLLDHDLTWHGELPIPDYVNAAPMGLSANIQLPYSASALNF
DESHTSVRSIAEGEGYGTPVAASSTVVAANTSDGSLDSSVYDQQDPPSSHRLQSSGASFINPP
AAGTYKKKKKRDPRLECPNFLAGRIPCSCPEEDELEPDVEVDALVTKKSKVAARCQVPSCG
EDISHLKGYHQRHRVCLTCANSPKVILKDQAHRYCQQCGKFHRLSDFDEGKRSCRRKLER
HNRRRRRRPVEGEGNMEDCEDPSVAWTATKNAHTPAASKGKSKGSNAVHLQKKKDAAR
DDQHASDSLSESVRRFEEDIISSSEGKLDDHPRVDVVLTSLNESAEQDSRNMKLCLKSSSPST
PHMSTNEWKCTDYISPCPTGRISFKLYDWNPADFPRRLREQICEWLKSMPAELESYVRSGCII
LTFFVAMPQLMWDKVLAGWKDQVQAFILKSGVKLLGSAELTVGTTEKSLCLENGRVTNAY
HRDLLIPIIVDLYPRSIEAGCCIQLYVFGFNFSGGKFLLSFGEQYVECSECEAVDRCQCTARGF
WNTLDDIEVQKVTVRLPDTPKFGLAYMEVENSQGLSNFIPVLVADRLVCADINKMKFRHQ
YSSSSRALDCSDNEAVKQVKFHPSSDLISDLGWTLSYMHGNHPNGSIETLNWQLQRLLVFF
SELHCHAVASYLVQTTFSKFLTQNIYNFHDLHLWNIHDTSKNMKAISCHDHSRMPVNYVSI
ITTGDRPSPHEIHNLSKEKDLELLDAATEESPLLQRNTQGEDSSCLLPMELPWLKEKVPIPGD
VWRQKSSFSWSLNCRRRPQGIGSASLQGGTRRFTRILVAVVGVVAVCTGACLMLQHPREVA
EFSMSFRRCLWGSTGTKHV

>ABR17971.1[Picea sitchensis]
MKQSDTRNPDVIRSSREQEGRETTAAMADLLDDSVASVLDAGNWEWDSVLDFSIGSLPHIL
PWQSEELSDDLSERLLPSQVLEVNQAREVQNASPRNRSQDAEEEEKAAASSRIRKREVQNA
SPRNRSQDAEEEEKAAASSRIRKRDPRLICSNFLAGRVPCACPEMDNMEEELSRKRAKVVV
LRCQVPSCEADISHLKGYHRRHRVCLCCANAATVVLDDVPQRYCQQCGKFHLLTDFDEG
KRSCRRKLERHNNRRRRKPVNVVEASVQEQTLRAESALQSADTEPHNNGKSGQASKSFSY
PGCGEKQSSSGSEERNISEVEQPYKGDSSLKNVGSRSDDVSKISSSSMPVKVSTELEGKLFPER
DIGSGEKFDDEDVRVVENSRAIMLVGPRNGDNGSTYAELNEQSYTGMPMEENSHKDRFPH
NISGRSEKDEGAGPLKSSLSSNRHDKHLSYTSVCPTGRISFKLYDWNPAEFPRRLRQQILQW
LADMPIELEGYIRPGCTILTAFIALPQFMWEKLFANSPGYIHSLLNGSESILSGKGNMLVYLN
DTIMQIKNGEACLVDTKMDMRIPKLLSVHPISFEAGYPIEIVACGRNLLQSKYRFLISFHGNY
LHYGTCEAIPLGNPGSSFRSKENIFHSSNHESFKIFIPSTDPRLFGPAFIEVENEYGISNFIPILIG
DKEICSEFQMLEQEVVKSGFCCRSNHGLAIGSSNSDICEQNVVKRQSILELLLDIGWVLKDP
EPDENKVPVNFVHIQRLNCLFSYLINGHLLAMTEKVLHSPKVVKMLRQVYGKSNEVNGA
DIKLLQNYVEHAWQSLRKKPNYKVTTDFQWFLDNVVLQGCPSSMKIATLSCNQQPRKVSQ
ESQEMEPLLEIQTETRPSSGIESIDTRVLSVEENNMLTPLLEKECSSSIASDMKYNSTWRGPKIE
VAEYTRKMKVCLKGDIRVDRRIFILAITVISVCAGICVVLQHPHQVMEISMSLRRCLSGLRKA
HEDISRP

>H.annuus r1.2|HanXRQChr08g0227251
MHNPPPPPPPPSSAFNMDEPSSSIWDWSQFLDFNIDDHLSTQSPLIPTSQDHLYPIEIEPEQPA
ADSFPVNSTANTNVRVRKRDPRMACSNFLAGRIPCACPELDAQLVAEEEEAAPGKKRTRT
VATSRCQVPGCETDISELKGYHKRHRVCLGCANASNVVINGESKRYCQQCGKFHVLLDFD
EGKRSCRRKLERHNNRRRRKPTDTKGPGLQSAADYDDGCDEAGKGGKFMLSETVAGEGP
SSSSVNARNIHSDSIPSLAASGETQTDEEKEKATHSLSDCDDKIAFSSMCTTGRISFKLYDWN
PAEFPRRLRHQIFQWLANMPVELEGYIRPGCTNLTIFIAMPRFMWMKLSEDPVVCIHDLLA

SPRNLLSGRHTFFVNLNNTIFSVMKGGRSVIKIKAVEKSPKLHYVQPTCFEAGKPIEFLACGS
NLLQPRLRFLVSFAGKYMKDDARVSICNQSDTSTTNLDHQFLSICAPRTELNVFGPGFIEVE
NESGLSNFIPILVAEEEVCSEIKIMQTKYYSTLRSRDSKSSSCEVAVNKFSEVLVDMAWLLKQP
IVEDTECAIMSSQLQRFTFLLNFLIEYESTTVLKRILHCMEMRVIGSGGMFEDDKTSLQETLN
HATEVLNQRLEKKVNLGSPSNDILSEDESCGDEVHSFVSTVNRVSPSGLEHEKIGLLNADCI
MSVTPYKERAKPSNNVLSYKTNRLFTPRALILAVALVTVCFGICEAVFHPHKATAIAVTIRRC
LFNGN*

>H.annuus r1.2|HanXRQChr07g0193871
MHNPPPPSPPPFTMDSIDDSSTSIWDWSQFLDFNVDDHLPFPTDLSPLISTSPDLYPIDDQPL
QPLTDSFPVISTSNTNARVRKRDPRMACPNFLAGRVPCACPELDAQLAAEEEEEAASKKKK
RAVTVRKSSGGSRCQVSGCETDISELKGYHKRHRVCLRCAYAGSVVIDGESKRYCQQCGKF
HVLSDFDEGKRSCRRKLERHNNRRRRKSTDSKASGPHLTAVYDDEAGKGGKSTSGETAAG
EKSSLAGGDHNSLAIIAQNIQSDSIPSLAASGETQTDEEKEKTTHSPSYGDDKTDFSSMCTTG
RISFKLYDWNPAEFPRRLRHQIFQWLASMPVELEGYVRPGCTILTIFIAMPRFMWLKLDEDP
VVCIHDLLASPRSLLSGRDTFFVNLNNTIYSVMKGGKSVIEIKVGGKSPRLHYVQPTCFEAG
KPIEFLACGSNLLQPRLRFLVSFAGKYMTNDVRVSPSCNTSSTNLDHQLLNIRVPHSEMDVF
GPAFIEVENESGLSNFIPILIADKEICSEIKIMQTKYYSTLRSNDSESSSCEVAVNKFSEVLVDM
AWLLKQPIVQDMERATMSSQLQRFNFLLNFLIEYESTTVLKRVLDSLKMRIIENGDIVETDR
TLLQGTVNRATEVLNQRLEKTVNHGLHPSDILLNNDDDPFISTVNQVHEESEKVGLLNAD
CVMNVTPYKEQPKKPSNRMFNYKTTRFFTLRLLILAVASVTVCFGICAVVFHPHKAAAIAI
NIHRCLFDDN*

>A.trichopoda|evm_27.model.AmTr_v1.0_scaffold00197.11
MEVGWEWSNLLLDDFSSIDGDAPSLPSEAAPYCSDWDALPLPSEDPQPQSMPQPEPSSATS
SVLLPHSSPLNPQTSSPAKVRKRDPRLTCSNFLTGHIPCACPELDEQERDEDEASRKRTKMT
AAAVVRCQVTDCEADISELKGYHRRHRVCLRCANATTVILDGQPKRYCQQCGKFHVLQD
FDEGKRSCRRKLERHNNRRRRKNLDFRHGVEGSHLTEKEPLSIMTIEDVFCNGEAGSGLGD
GCGEASLHLSDQMKDTNTRSLLECEEGHESPFHSVMGKMNPSLQNPQSDDVLSIVTSDKA
QRKSGNDHSRPSLSSSLCDNQAGYSSVCPTGRISFKLYDWNPAEFPRRLRHQIFQWLASMP
VELEAYIRPGCTILTVFIAMPHFMWEKLFKDATVFLDDLVNAPGSLLSGRGRIFIYLNNWIF
QVLRGGTSLANNMMDMRVPKLHYVHPVCFQAGKPMEFVACGRNLFQNKFRFLVSFAGK
YLQYDSCQAISLEETKSFRGIGGSFIHCSDTEMFKIRIPSTDPKLFGPVFIEVENVSGISNFIPVL
LGDKWTCSELQNFELMLGEAVCHEYDNNFVMNAACRSSNIADSDKKCFSELLLDIAWLL
KEPHFDHDRNETELNSMHFERWSCLLRFLVRNGLVFVLEKVLKSPMVLMKLEESENQTSD
VDMRLFRKYVNQARELVDQQNKRGRLRLRSKNITSQGSSPPTRDINANILNDKHCTNQEM
GSKGERDHEKSIATYLVQGGDMRTPLLSKEVVMRVNHSPCPIERPRFYPSAGIIVSPRLFLIV
LGAVVMCCGVCIVLRHPHEVWEFSISLRRCLTGTPKR*

>S.lycopersicum|Solyc01g080670.3.1
MHNQPFSSSQTGVPTELRNTQMISSLLSGDDPAASSNFDWSDLLDFDLHEQLNISFDDPLH
QEQQPETEFVAPVIPSSEDSPHSQDTDAGRIRKRDPRMACSNFLAGRIPCACPELDEKMEEE
EMAGIGPGKKRARTVRASAGAGARCQVPDCEADISELKGYHKRHRVCLRCANATSVVLD
GHSKRYCQQCGKFHILSDFDEGKRSCRRKLERHNNRRRRKATDTSKTSAEKESQQLTTAD

DVSGDDDIVKDNTCMGSQLGEKEILLESEGHVPICSTQGIQNNHSDSFTASGETQVDAEKE
NYKNSHSPSYYDNKSALSSVCPTGRISFKLYDWNPAEFPRRLRHQIFQWLASMPVELEGYIR
PGCTILTVFVAMPTFKWGKLLEDPAAHLYELIASPGNMLRGRGSFLIYLNNMVFRVTKGEN
SVVKVKLKGPAPKLMSIYPTCFEAGKPMEFFACGSNLMQPRFRFLVSFGGRYLGNDINVVP
SDCKYEGDSSSTEHQLLKIHVPRTEADLFGPAFVEVENESGLSNFIPILIAEKDICAEMKEIQR
KFCSGGSECTAVCSPCEASTSRKSEFSEFMLDVAWLLREPSSENVQILASVQMQRFNYLLNIL
MESQSTIILERVLSYFENIVKRNMLAGITDADMTLFQKNILEKNILLKERLHLKEYFAGDSGQ
IMQELPNLQDTAVPHKHNIEFGPTYWELTSRVPLLDAELPLRVKEQQSGKSCGFLVRKTLL
TSRTLVFVISGFALCLGLCATFLHPRKVGDIAMTIRRCLFDKT*

>M.truncatula|Medtr2g020620.1
MESKSQPILPPMDPPEDLSSVWDLSYLLDFDDDIPQLPPLPNPNPTPEENERIRKRDPRLTCS
NFLAGQVPCACPELDALLEDNGLPGKKRARTARLIFSELKGYHRRHRVCLRCANAATVVL
DGDVKRYCQQCGKFHVLSDFDEGKRSCRRKLERHNTRRRRKAVDSAVGVDNEVQTVTQ
NDDSNCDGELGIDYSNLSRENIEKRALQDHEEEPVVNGSSTPETQNINGDSVVSFVASAET
QANIGKDVSDPSKSPSYCDNKSDYSSMCQTGRVSFKLYDWNPAEFPRRLRLQIFQWLASMP
VELEGYIRPGCTILTIFIAMPNIMWINLLKDPMYYVRDLAAPRNMLSGRGTALIHLNDMIFR
VMKDGISVTKVEVNMQAPRLHYIHPTCFEAGKPMEFFACGSNLLQPKFRLLVSFYGKYLKC
EYCAPSPHNSAEDNISCAFDNQLYKICVPHIEENLLGPAFIEVENESGLSNFIPVLIGDKEICT
ELKILQQKLDASLLSKQFRSASGSSICSSCEAFVHIHTSSSDLLVDIAWLLKDPTSENFDRMVS
ASQIQRYCYLLDFLICNDSTIILGKILPNLISITKSMKSNISDVDMDQLLKGMCNARDAICRK
GGGIVLNSKMEGFKPAQCSSQNAKLSVVEVNSQGIQFRADAELGVLSSLASDEKNQKIPLL
KRDIIMNMEELPKRCDHQNLTRGFLRSRPTTFVLVSLVVCLAVCVSVFHHGRVNELAVSIR
RCLFNH*

>P.trichocarpa|Potri.008G197000.1
METSSPSPPPPPSASQHGGDMEIHYPPITTDWDWSDLLDFAVDDRIPLSFDTPGDLTQTIDN
PTPEIESQQVQLPVPDRVRKRDPRLTCSNFLAGIVPCACPEVDELLREEEATLPGKKRVRVA
RAGSSIARCQVPGCETDISELKGYHRRHKVCLRCATATAVVLDEQTKRYCQQCGKFHVLS
DFDEGKRSCRRKLERHNNRRRRKPADSSKASAGDKEVQGDLLTEDTTTCDAEAEKDGCSS
GQMAEKEGLVESEDGHVSTMNSDPNSQNVTSDSGVSFTAFGDVLMDGGKDDSKFLFSPSH
CDNKSDYASMCPTGRISFKLYDWNPAEFPRRLRHQIFQWLANMPVELEGYIRPGCTILTAFI
AMPTFMWVKLVEDPVSYLNDLFGSGKMLSKKGRMRVYVNNMIFNVTKDGNSVMKVNV
EGHAPRLHYVHPTCFEVGKPIEFVVCGSNLLQPKFQFLVSFAGKYLAHDYCVALPQAHTK
GGPGLHHQLYKILTHCNEPNLLGPAFIEVENESGLSNYIPILIGDTEICSEMKIIQQRFDASHS
LIIGSECEVSTMRQTALSEFIMDIAWLLKEPSAENSQQMMTSFQIQRINSLLNFLLHHESIIILD
KILKNLKIMMDKKEANGMVNGTSDTNMRLLQSYMDYASNIRHEKLQRSEVLKHHLEFSG
KENNCISGSCCGNNKESVALSTENLEQRPNGVLGVMGNSNFTVRSDEFPLLTKDVVMRM
NLVNERPKKSCGLVFSNRVLKYRPSFYVIALIAVCFGVCAIVLHPHKVSKLAVSIRRCLTERF
*

>P.trichocarpa|Potri.010G026200.1
METSSSSSPPPPPPPQHDGDMEIHLPPVTTEWDWGDLLDFTVDDQFPLSFDTVVDVTQPID
NPTPEVESQQLEAPVSDRVRKRDPRLTCSNFLAGIVPCACPEMDELLLEEEAALPGKKRVR
VARAGSSIARCQVPSCEADISELKGYHRRHRVCLGCANATAVVLDGETKRYCQQCGKFHV

LSDFDEGKRSCRRKLERHNNRRRRKPADSSKGSAGDKEVQGDLLTEDTTCDAEAGKDGL
WSSSQMVEKEGLVESEDGHISALNSDPISQNVNSDSGVSFTASGDTRMDCGKDDSKLPFSPS
ICDNKSAYSSVCPTGRISFKLYDWNPAEFPRRLRHQIFQWLASMPVELEGYIRPGCTILTAFL
AMPTFMWVKLLEDPASYLNDLLGSGKMLSKKGRMRVYLNNMIFNVTKDGHSVMKVNV
KGHAPRLHYVHPTCFEAGKPMEFVVCGSNLLQPKFRFLVSFAGKYLAHDYCVALPQVHT
KGGSGLHHQLYKILTHCIEPNLLGPLFIEVENESGLSNFIPVLIGDRDVCFEMKIIQQRFDVS
HSLIFGSECEVSAMRQTAFSEFSTDIAWLLKEPSAENFQQTITSFQIRRFNSLLSFLLHHESIIIL
DRILKNLEIMMDKREVNGMFDDTSDTNMRLLQSYMEYASNILHKKKRSEVLKHHLECPG
QEYCVSGSCCVSNKPAVVISSEGLEQRPDGGLGVMANSKCIVKSEEVPLFNRDERPKKSCG
LVFSNRVLKYRPSVFVIAIIAVCFAVCALLLHPRKVSKLAVSIRRCLTDRY*

>V.vinifera|VIT_205s0020g02160.1
METSSLPPPLQQSPSPTGRPRVSEMEVQHPMTEDASALWDWGDLLDFSVDDPFTISFDSDH
NLEVSPSPEPLTREAPDAPERVRKRDPRLTCENFLAGRIPCACPELDEMILEESAPGKKRVRT
ARPAAGRARCQVTGCEADISELKGYHRRHRVCLRCANASVVILDGQNKRYCQQCGKFHI
LSDFDEGKRSCRRKLERHNNRRRRKPIDSGGTVEKEIQGELISEDAAHDGEADKDSLCLSSQ
LIEREPLLESEDGHFSTLCSVPGSQNIQSDGIVSFVGSGEAQIDGGKNDSKYTLSSSYCDNKSA
YSSPCPTGRISFKLYDWNPAEFPRRLRHQIFQWLASMPIELEGYIRPGCIILTIFIAMPKFMWD
KLLEDPASYVHDFVAAPGKMLSGRGNVLVYLNNMIFRVTEDGTSVMKVEVKMQAPKLHY
VHPNCFEAGKPMEFVACGSNLLRPKFRFLVSFAGKYLSYDYHVVFPRGKIEGDTAGSLDHE
FCKIYIPHTEPNAFGPAFIEVENDHGLSNFIPIFIGDKEICSEMKILQHRFDASLCSKGSQFFAK
DPSDSCKVSVLGQTAFSEFILDIAWILKEPASENIQRSLTSSHIQRFNCLLNFLIHNESTTILEKI
LQSLKILIDNMDLNIQVNGATDTDLRLLYKYMDHASKILHQKLHSSGGLVLHSGNSVTKG
DHPSCFHNNMLPVVFPPEDTKISANGGLAAMASSTSTDRSETVSLLNREVVMNMNSIKEQ
PRKSCSLIFSKKAMTSRPFLYMIVAAAACFGICAVLLHPHEVGKLAVSIRRCLFDNS*

>S.fallax|Sphfalx0151s0006.1
MGHSEDTTSGWDWDSVLLLVNPAVDTTSDELVRETLDSACGFSVGQSWPEFEDLHSSRPG
NLAATAAAEETTTKQQQNTQTGPQSSDTVEKAVSRKEGAQHRDPRLDCPNFLAGRVPCA
CTDNEEEDEGGSQSKRVKLSARCQVPSCAADLASLKGYHQRHRVCLRCANASSVKLHQH
LHRYCQQCGRFHVLSDFDDEKRSCRRKLERHNNRRRRKVQESGDDTATLTIDNDNPSPDE
GKDGKSTGEEKATVDREMQSEVDQSHNLIASTLAPPLVEQSKTTEEAHASLMGGSANPKP
SNSNSPRRQIVEKGEVGVRKGGVDEDSLLALLLEESPNEVDGLPLTSDLQPRISSRELDHSST
YTSRYPTGRISFKLYDWNPGDFPRNLRQQILQWLSNMPVELEGYIRSGCTILTLFIAMPQSM
WDKLYADWAGAVTKLVQGSQSVAGFWDRGYFKAKLGHKTVHFEDGQVVNRSGGKDEC
MPCMPVLHSVQPICFEAGIEGHLTVFGHNLLQPNTRLLVSTGGKYLDACVVQSQHGNKLD
KYQIIVPALKHSQVGPVFIEVENEGRTSNSMSVLVGDRDLCSALERLDLQASRGCEQDLVFD
LCWMLRDSSCQDYGACSRLRDLFLFAKACGWVRLAEYLLQVAGRKGMLSEVLSIDDMDP
TCADKTFWWGRNMDTQGLTVLPAMDFEWDDGHLLPTFRKSGKYGEKDTHVILPKGRDA
LVPLLAQRRQRGSCNQRWGTHRVIAVVASVTVACAGVCLVLQHPNEVIQMSTSLRRCLW
GQ*

>S.fallax|Sphfalx0023s0010.2

MMGHSVETMMGWEWDSLLLLINPAIADTSDGGGVVGDALLDSAACGFSVGQSWPEFEDF
DTGPGILVEEEEETQRQQFAKTKPQYRDSTEKAGNEERRDPRLDCPNFLAGRVPCACSDNE
DEEEEGGGSHSKRVKLSARCQVPACGADLASLKSYHRRHRVCLHCANATSVKLHQRLHR
YCQQCGRFHVLSDFDEEKRSCRQKLERHNNRRRQKVQESGEDTTTITIDNDNPSLEEGKDG
KSMVEEKATVDNEVQSDADRSCSLVAPTIPLLAEQLKTTDEAHPSLPNPNASNTMSPKEQS
VEKGEVGVRKVGVDEDSLLALLLEEAPDEVNHLRHMSDLPTVISTRERDRSSTYTARYPSG
QISFKLYDWNPGDFPRNLRQQILEWLSNMPVELEGYIRSGCTILTLFIAMPQSMWDKLYVD
WAGAVRKLVQGPQSVAGFWDQGYFKAKLGWKTVHFVDGQLVNRSGGKKECMPCMPVL
KSVQPICFEAASEGQLTVSGENLLHPNTRLLVSAGGKYLNAYVVQSCHENEEDEWKIIIPAL
DCSQVGPLFIEVENEGGTSNSMTVLVGDRNLCSELERLELEVSRGCEQDLVFDLGWMLRDS
SCQHSGACSRLQDLFLYAKACGWHSLAEYVLQAAGRKGMLPEVTKVGDKTYPFRGDMD
APQALMVLPTMVLEWNSAHLLPPFKKDRKFGKKDVHITVDSGRDTLVPLIPKQRRGSCNQ
SWGTYRVMAAVATVTAACAGVCLVLVHPDEVIQLSTSLHHRLWGQ*

>T.plicata|Thupl.29378511s0017.1
MADLPGDPTNWDWENILYFPDVSPNILPWHAEEPSANFSEDLLAPLETLRILEVQNSFEEN
HRSQDVTEEETASRIRKRDPRLICENFLAGRVPCACPEMEENEEEEELDGSRKRAKVGIVRC
QVPSCEADISHLKGYHRRHRVCLSCVNAATVVLDDVPQRYCQQCGKFHLLPDFDEGKRSC
RRKLEKHNTRRRRKPIGDKASVQGRTSRTKSPLQNADAEPYIYATSAQESKSLSSLGTGEKQ
ASSVSEEFQISEVEQPYKDRSCVDIVELKSAGDNKVSNSSRPVIVPIELEGTLIPDNDRGCGEY
LNDRERKFIENNQASPLKTLVGSQDHDDSTYTEFNEHSYTGLLIEDNSHKDKHLHSSSGWN
QRVKGTGPSRSSLSSTGQEKNLPYTSVCPTGRISFKLYDWNPAEFPRRLRQQILQWLANMPI
ELEGYIRPGCTILTCFIALPQYMWEKLSADATSYICSLLSGSESILSGRGNMLVYLNNIVMQIE
NGEASLVNTKINQRVPQLYSVHPVFIEAGQPIEIVACGKNLFQSKFRFLISFGGSYLHHHSCE
AIPLQKENSHFRTKENVFHASNHESCKISIPSTDSRLFGPAFVEVENEAGISNFIPILIGDKQIC
SEFELFERELMKCRICCRSGSEDVAITSPSFDMHKSNLVKQQSILDLLLDIAWVIKDPEPEEN
EIQVNHAHLQRLNHLFSFLIDGQFHSVMDKLLHSPKVIKIIKQVYGPSNHFNDADVELLYN
LVEHAWKSVRKKTNYKDIGNFQQFVEKVLLQGQPGNQQPYKCSDESQVVEEIHTRIISSSGI
ESGDTRVLSVEENNVFMPLLEKEFCSDISTGLKPKPNFGDQKMIDVAGCAKMLRIDLNRDI
RVDRRVFILTITFIAVCAGICVALQHPHQVMKISMSLRRCLHGLHKGQGVASDP*

>C.richardii|Ceric.22G034200.3
MADSNYVLESWDLETLFASSLLDHDLTWHGELPIPDYVNAAPMGLSANIQLPYSASALNF
DESHTSVRSIAEGEGYGTPVAASSTVVAANTSDGSLDSSVYDQQDPPSSHRLQSSGASFINPP
AAGTYKKKKKRDPRLECPNFLAGRIPCSCPEEDELEPDVEVDALVTKKSKVAARCQVPSCG
EDISHLKGYHQRHRVCLTCANSPKVILKDQAHRYCQQCGKFHRLSDFDEGKRSCRRKLER
HNRRRRRRPVEGEGNMEDCEDPSVAWTATKNAHTPAASKGKSKGSNAVHLQKKKDAAR
DDQHASDSLSESVRRFEEDIISSSEGKLDDHPRVDVVLTSLNESAEQDSRNMKLCLKSSSPST
PHMSTNEWKCTDYISPCPTGRISFKLYDWNPADFPRRLREQICEWLKSMPAELESYVRSGCII
LTFFVAMPQLMWDKVLAGWKDQVQAFILKSGVKLLGSAELTVGTTEKSLCLENGRVTNAY
HRDLLIPIIVDLYPRSIEAGCCIQLYVFGFNFSGGKFLLSFGEQYVECSECEAVDRCQCTARGF
WNTLDDIEVQKVTVRLPDTPKFGLAYMEVENSQGLSNFIPVLVADRLVCADINKMKFRHQ
YSSSSRALDCSDNEAVKQVKFHPSSDLISDLGWTLSYMHGNHPNGSIETLNWQLQRLLVFF
SELHCHAVASYLVQTTFSKFLTQNIYNFHDLHLWNIHDTSKNMKAISCHDHSRMPVNYVSI
ITTGDRPSPHEIHNLSKEKDLELLDAATEESPLLQRNTQGEDSSCLLPMELPWLKEKVPIPGD
VWRQKSSFSWSLNCRRRPQGIGSASLQGGTRRFTRILVAVVGVVAVCTGACLMLQHPREVA

EFSMSFRRCLWGSTGTKHV*

>L.japonicus Lj1.0v1|Lj6g0005792.1
MEPQSQSQSQSQSQLQLQASLPLPPPPHMDPDDPSSSVWDLTYLLDFNLDDDAPVFPFDD
NNLSETPLTDPQDQEEETHKVRKRDPMLTCPNFLAGRVPCACPEMDALLEEQGLPANKRP
RTAPSAARCQVPGCEVDISELKGYHRRHRVCLRCANAPTVVFNGEAKRYCQQCGKFHVLS
DFDEGKRSCRRKLERHNNRRRRKAPDSRAAADHEPQAVTQNEEFSGDLEAGKDCSNLSSE
IVEKEASPPSRDHEEEPVAVWSSTLDTPNINGDSVVSFVASGTSAQVNGVNDVSNISNSPPSY
CDNKSAYSSMCQTGRISFKLYDWNPAEFPRRLRHQIFQWLASMPLELEGYIRPGCTILTIFIA
MPNIMWINLQKDPLYYVHDLVAPRKMLSGRGTTLVHLNDMMFRVMKDGTSVTKVEVNM
QAPRLHYVHPTCFEAGRPMEFFACGSNLLQPKFRFLVSFSGKYLKYDYCVPSPHNWSEDNI
SCVNDNQLYKIYVPHTDECLFGPAFIEVDNESGLSNFIPVLIGDKEICTEMKTLQQKLDASLP
SKQFQSASGDSICSSCEALAHRHTASSDLLVDIAWLLKDPTSESFDRVMTASQIQRYCYLLDF
LICNDSTIILGRILPNLIILTESMKSNFVINRISDIDMTELLKCMHNARDAICQKHRKDGGVV
APSEMEDFEAAQSGSQNNMQSVVAVNYQGILPGPDSNLTVLRCPTSNDKNEKIPLLKQEII
MNVEEFPKRCDRRYLTRGLLSSRPAIFMAVSVAVCLGVCVSVLHHHGRVSELALSIRRCLFN
Y*
>C.violacea|Clevi.0003s1907.1
MSSASRSSPAPDMDFLPQAMVDDDPSTFSTAMWDLGDLLDFAVDERLLVSLDPDQPPFAS
APPPPSPPPPPPQMESEWNPSPDSGSDRVRKRDPRLICSNFLEGRIPCSCPEIDQRLEEAELPT
KKRVRSGGRGGSGVARCQVPGCEADISELKGYHRRHRVCLRCANASSVLLDGETKRYCQQ
CGKFHLLSDFDEGKRSCRRKLERHNNRRKRKPPGDKGGGAEKQQQAAMQNENSIIDVDD
GKDNICSIDQRAEKEASVDFEDQHFSAQGAVPVAQSINADSFVSFEGQGGAQTDEGKNDT
KFEHSPSYGDNRSAYSSECPTGRISFKLYDWNPAEFPRRLRHQIFQWLATMPVELESYIRPG
CTILTVFIAMPEIMWAKLSKDPVAYLDEFILKPGKMLFGRGSMTVYLNNMIFGLMRGGTSV
KRVNVKVEAPRLKYVYPTCFEAGKPIELFICGRNLLQPKFRFLVSFSGKYLPYNYSVISSTKED
GKGSARCCNKFYKINIVISDPNLFGPAFVEVENESGLSNFIPLIIGDAAICSEMKLIEQKFNAT
LFPDGRVSACCSSMCFCGDFEQRQTAFSGLLLDIAWSVKVPTSDCSEQTMNLCQIRRYNRV
LRYLIQSNSASILEKVLQNLEGLVNKMEPDSVIHCNSDGDVRLLHENMNLARDIYKKRQSH
EQSRTNLGNVLPSSNCGCACESSLQKDSPSRILTINQDSEAGVDTKEGKQSVARRETDPLLS
KEFVMNMDGMRDWPRKSCSPIHPAQAVRSRPTVFLISTLFICFAVCAVLYHPNRVTELAVA
IRTRLALKT*

>S.alba|Sialb.0193s0063.1
MSSSQSPSLPEMEIQPPALLDDDPSTYSSSALWDWGDLLDFAADERLLVSLDSDQTPFSPVP
PPPPLLIATQSETYPSPDESGSGSDRVRKRDPRLLCSNFVEGLVPCSCPELDQKLEEAELPKK
KRVRGGSGVARCQVPGCEVDISELKGYHRRHRVCLQCANASFVVLDGEDKRYCQQCGKF
HVLPDFDEGKRSCRRKLERHNNRRKRKPVDKGGVASKQQQVLSQNDNSVIDVDDGKDN
GCSSDQRVEQEASLNFEDRHIPTQGPVPFTHSINADNFVSGTGSGEAQPDEGINDTKFELSP
SGGDNKSAYSTVCPTGRISFKLYDWNPAEFPRRLRHQIFQWLATMPVELEGYIRPGCTILTV
FIAMPEIMWAKLSKDPVAYLDEFILKPGKMLFGRGSMTVYLNNMIFRLIKGGTTLRRVDVK
LESPKLQFVYPTCFEAGKPIELVVCGLNLVQPKCRFLVSFSGKYLPHNYSVVPGPGQDGKRS
CNNKLYRIHIVNSDPSLFGPAFVEVENESGLSNFIPLIIGDKAICSEMKVIEQKFNATLFPEGQ
DITAACCSLTCCCSDFKERQSTFTGLLLDIAWSVKVSSSECTEQTVNRCQIKRYNRVLNYLIQ
SNSPSILGNVLHNLEMLVKKMEPDSFVHCTCDCDVRLLHENMSTARKKQSHVDSKVNPV

TSACCCESSFQKEFPSGVLNFNQEPEAGLGCNERIQTASTDTGRKETDPLLNKEVVMNVND
IGDWPRKSCIPVHSAQTFRSRQTVLYIATFAICFVVCAVLYHPNKVTQLAVAIRTGLAHKL*

>S.alba v3.1|Sialb.0712s0028.1.p
MSQPQPPPAPEMDIQPPALLDDDPSSAMWDWGDLLDFAADDRLLFSPAPPPPPMIAMQSE
SYPSPDESGSGSDRVRKRDPRLLCSNFVQGMVPCSCPELDQKLEEAELPKKKRVRGGSGVV
RCQVPGCEVDISELKGYHKRHKVCLTCANASSVVLDGEDKRYCQQCGKFHVLPDFDEGK
RSCRRKLERHNNRRKRKPVDKGGVASKQQQVLSQNDNSVIDVDDDDGKDNTCSSDQRM
EQEASLISEDRNIPTQDSVPFPHSINAGSFVPVTGSGEANPNEGMNDTKFELSPSPGDNKSD
YSTVCPTGRISFKLYDWNPAEFPRRLRHQIFQWLATMPVELEGYIRPGCTILTVFIAMPEIM
WAKLSKDPVAYLDEFILKPGKMLFGRGSMTVYLNNMIFRLMKGGTTLKRVDVKLESPKLQ
FVYPTCFEAGKPIELIVCGLNLLQPKCRFLVSFAGKYLPHNYSVVSAPEQNVKRSCNNKLYR
INIVNSDSNLFGPAFVEVENESGLSNFIPLIIGDKAICSEMKLIEQKFNAALFPEEQDVAACC
CSLTCRCRDFKERQSTFTGLLLDIAWSVKVPSAACTELTVNRCQIKRYNRVLNYLKQSNSPS
ILGNILQNLEILVKKMEPDSLIHSKCDCDVRLLHENMNLARKQQSDEDLKANPVTSACCC
ESSFQKDIPSRININQDPEAGLDCEERKQVTSPDTGGKETDPLLKKEVVMNVSDIGDWPRKS
CMSIHSAQAFRSRQTAFLITTFVVCFAVCVVIYHPNKARQLAVAIRTRLAHKL*

>CqSPL21
MDLPPLTGGGEESGAPFEWSDLFDFTIDDQLLLNLDVSDHPKEQPPVLLPPVTNAEDNGKT
VAVNGGESGSSDRVRKRDPRMICKNFLAGRVPCACPELDALMAEEEEEEAGPGKKRPRMG
RTPGVAKCQVPGCEVDIRELKGYHRRHRVCLVCANATSVVIEDFDEGKRSCRRKLERHNN
RRRRKSADYRGTVEKEPQGDVQTEDVFSDGEAGKENAWSGGQIEKEDSKDKTLSNLCSAL
ESQNIESDSNLTFTPVDKVVDNLEREYSPPSDTKSAYTSACPTGRISFKLYDWNPAEFPRRLR
HQIFQWLASMPVELEGYIRPGCTILTVFVSMPQYMWVKLFEDPVSYVQNSVGHGGILSGRG
AALVYLNDLRFRVMREGASVMKVKVAVRAPRLHYIYPPCFEAGKPMDFVACGSNLLQPKF
RSLVSFAGKYLAHDYYVAFPRGKEDKPAIDYDYQLCRIYVPHTEPSYFGPAFVEVENECGLS
NFIPILIGDAHVCSEIKLIHQKYDCSNCRKKSQCMPSGSSYGTCEVSCSRQAALSEFMLDVA
WLLKQPCSEKLHCILTSSQIQRYNCLINAHNKELILKQGGECVSNVYTCLGSFSRDDMLSVD
HTACQVTKLRAEMNCTFLEHSDSQGEGESISLINREVAMNVNDSRDWPKNSCHSVISKKFT
STRPFIYAIAVVAVCFGVCAVVLHPYKVTKFAVTIRRCVFDNSS

>CqSPL22
MDLPPLTGGGEESGAPFEWSDLFDFTIDDQLLLNLDVSDQPKEQPPVLLPPVTNAEDNGKT
VAVNGGESGSSDRVRKRDPRMICENFLAGRVPCACPELDALMAEEEEEETGPGKKRPRMG
RTPGVAKCQVPGCEVDIRELKGFHVLSDFDEGKRSCRRKLERHNNRRRRKSADYRGTVEK
EPQGDVQTEDVFSDGEAGKENAWSSGQIAEKEDSKDKTLSNLCSALESQNIESDSNLTFTPV
DKVIDNLEREYSPPSDTKSAYSSACPTGRISFKLYDWNPAEFPRRLRHQIFQWLASMPVELEG
YIRPGCTILTIFVSMPQYMWVKLFEDPVSYVQNSVGHGGILSGRGAALVYLNDLSFRVMRE
GTSVMKVKVAVRAPKLHYVYPPCFEAGKPMDFVACGSNLLQPKFRSLVSFAGKYLAHDYY
VAFPRGKEDKPAIDYDYQFCRIYVPHTEPSYFGPAFVEVENECGLSNFIPILIGDEHVCSEIKM
IHQKYDCSNCRKKLQCIPSGSSYGTCEVSCSRQAALSEFMLDVAWLLKQPCSEKLNCILTRL
LKEYIAQANDFLRQSAYNKELILKQGGECVSNVDTCLGSFPHDDLLSVDHTACQVTKLRAE
TNHTFLEHSDSRGEGESVSLINREVAMNVNDNRDWPKKSCHNIISKKFTSTRPFIYAIALVAV

CFGVCAVVLHPYKVTKFAVTIRRCVFDDSS

>A.hypochondriacus v2.1|AH020154-RA
MELPSLTTTDEQLPTPFEWSDIFDFTIDDQLLLNLDVHDQQHQLQSPIVLPPPNVDSEQSQK
PPTVSPPQTSSSDRVRKRDPRMVCENFLAGRVPCACPELDAQVMEEEEEAAPGKKRSKMG
RTPGIAKCQVPDCQADIRELKGYHRRHRVCLVCANASSVLLHGETKRYCQQCGKFHVLSD
FDEGKRSCRRKLERHNNRRRRKSVGSRGALDKEYQGDLHTEDAFSDGEAGKDNSWSSNQI
TEREDSKDGTLSNLCSALESQNIESDSIVTSTQVDKVINNLERDCTPPSENKTAYTSVCPTGRI
SFKLYDWNPAEFPRRLRHQIFQWLASMPVELEGYIRPGCTILTIFLSMPQNMWVKLIDDPVS
YIRSSVGFGGMLSGRGVAMIYLNDMSFRIIRDGASVMKVKVAVRAPKLHYVYPPCFEAGKP
MNLLACGSNLLQPKFRSLISFAGKYLAHDYYVAFPREKEDESASYCDHQFFRIYVPHTDPSY
FGPAFIEVENECGLSNFLPILIGDEHVCSEIKTIHQKYDCSYCRKKFQNRPSGSSDDTCEVSCS
RQAAFSEFMLDVAWLLKQPRSEKLHRVLTSFQIQRFNCLLKFLISNKSTSILEKILQSLENVCD
EMESSDAADVTDTDRRLFKKCIAHARDFLLQSVHENELVVCQARTCAQKDYRCLENFPCH
DDLSYDRPVCQVTDYSPSDGESVSLVNGELTMKITENKEWSRNSCNHMMSKKLINSRPFIY
AIAVVAFCFGLCAVVLHPYKVTKLAVTIRRCVLNDAS*

>AAA17066-RA
MDLPPLTGGGEESGAPFEWSDLFDFTIDDQLLLNLDVSDQPKEQPPVLLPPVTNAEDNGKT
VAVNGGESGPSDRVRKRDPRMICENFLAGRVPCACPELDALMAEEEEEETGPGKKRPRMG
RTPGVAKCQVPGCEVDIRELKGFHVLSDFDEGKRSCRRKLERHNNRRRRKSADYRGTVEK
EPQVDVQTEDVFSDGEAGKENAWSGGHIAEKEDSKDKTLSNLCSALESQNIESDSNLTFTP
VDKVIDNLEREYSPPSDTKSAYSSALFEDPVSFVQNSVGHGGILSGRGAALVYLNDLSFRVM
REGASVMKVKVAVRAPKLHYVYPPCFEAGKPMDFVACGSNLLQPKFRSLVSFAGKYLAHD
YYVAFPRGKEDKPAIDYDYQFCRIYVPHTEPSYFGPAFVEVENECGLSNFIPILIGDEHVCSEI
KMIHQKYDCSNCRKKLQCIPSGSSYGTCEVSCSRQAALSEFMLDVAWLLKQPCSEKLNCILT
RLLKEYIVQANDFLRQSAYNKELILKQGGECVSNVDTCLGSFPHDDLLSVDRTACQVTKLR
AEMNHTFLEHSDSQGEGESVSLINREVAMNVNDNRDWPKKSCHNIISKKFTSPRPFIYAIAL
VAVCFGVCAVVLHPYKVTKFAVTIRRCVFDNSS

>BBB08709-RA
MDLPPLTGGGEESGAPFEWSDLFDFTIDDQLLLNLDVSDHPKEQPPVLLPPVTNAEDNGKT
VAVNGGETGSSDRVRKRDPRMICENFLAGRVPCACPELDALMAEEEEEEAGPGKKRPRMG
RIPGVAKCQVPGCEVDIRELKGYHRRHRVCLVCANATSVVIEGESKRYCQQCGKFHLLSDF
DEGKRSCRRKLERHNNRRRRKSADYRGTVEKEPQGDVQTEDVFSDGEAGKENAWSGGQI
EKEDSKDKTLSNLCSVLESQNIDSDSNLTFTPVDKVVDNLEREYSPPSDTKSAYTSACPTGRIS
FKLYDWNPAEFPRRLRHQIFQWLASMPVELEGYIRPGCTILTVFVSMPQYMWVKLFEDPVS
YVQNSVGHGGILSGRGAALVYLNDLSFRVMREGASVMKVKVAVRAPRLHYIYPPCFEAGK
PMDFVACGSNLLQPKFRLFPNPDDLKKVYKEYGAFACGLDYLSQIHVMNARNEEELLSW
WANYGASTPLLQGLALKLISQPASSSCCERNRSTYGTIQTTKRNRLTSSRVEDLVYVHMNLR
LLARKKNEYREGPSSYWDIGDVMDVDHILELVELSLNEPEIEAMTLEVENECGLSNFIPILIG
DAHVCSEIKMIHQKYDCSNCRKKSQCMPSGSSYGTCEVSCSRQAALSEFMLDVAWLLKQP
CSEKLHCILTSSQIQRYNCLIKFLISNKSTSILEKILQSLQIVFDGMNSCDVINNDTDRDRRLLK
EYIAQANDFLRQSAHNKELILKQGGECVSNVDPCLGSFSRDDMLSVDHTACQVTKLRAEM

NRTFLEHSDSQGEGESISLINREVAMNVNDSRDWPKKSCHSVISKKFTSTRPFIYAIAVVAVC
FGVCAVVLHPYKVTKFAVTIRRLAVLYANNNKSLSKSKHIDVKFRAVKERVESKQLSLEHIN
TNSMITDPLTKGLPTKVFHEHTARMVVMPPKDI

>EL10Ac3g06527.1
MEIPPLTAGEEESATQFEWSELLDFTIDDQLLLNLEVSDHGENQLPVVYPTESGGELEKSLTE
NGAETGSSDRVRKRDPRMSCENFLAGIVPCACPELDAMMMMEEEEAGPGKKRPRIGRMP
GVARCQVPGCEADIRELKGYHRRHRVCLVCANATSVVLDGQSKRYCQQCGKFHVLPDFD
EGKRSCRRKLERHNNRRRRKSADSKGAVEKELDGDLQTEVFSDGEAGKENAWSSSQIAEK
EDSKDGTVSNLCSALESQNIESDSILTFTHVDKAIDNSEQDYSPLDTKSAYTSVCPTGRISFKL
YDWNPAEFPRRLRHQIFQWLSSMPVELEGYIRPGCTILTIFVSMPQHMWVKLFEDPVSYVRS
SVGHGGMLSGRGAALVYLNDTSYRVMRDGASVMKVKVAVRAPKLHYVYPPCFEAGKPM
DLIACGSNLLQPKFRSLISFAGKYLAHDYYVAFPRGKGGHDTATDYDHQFCRIYVPHTEPTY
SGPAFVEVENECGLSNFIPILIGDEHVCSEIKMIHQKYDCSYCRKKLQYKPIGSSRRTCEGSCS
RQAALSEFTLDVAWLLKQPCSEKLHCILTSYQIQRFNCLLKFLMSNKSTSILEKVVQSLQFVF
DEMNSSEVTHDVNDTDRRLFTEYISHARDFLHQSAYENEVMLQQAGDGVPKGDKYLESFP
HDDVLSVDGTVCQGTEMKADMNCPLLEYSNCQGEGESNSLMNAEVAMHVDDNREWPR
KSCNYMISKKVINPRPFLYMIAVVAVCFGVCAVVFHPDKVNKFAVTIRTCVFDNSS*

>Spov3_chr3.03296
MDLPPLTGGGEESGTPFEWSDLFDFTIDDQLLLNLDSSDNQKIQIETPVVTPAAVGDGEGFG
KTLPVNGGGEGSADRVRKRDPRMICENFLAGRVPCACPELDAMMAEEEEEEAGPGKKRP
RMGRTPGVAKCQVPGCEVDIRELKGYHRRHRVCLVCANASSVLIDGQSKRYCQQCGKFH
VLSDFDEGKRSCRRKLERHNNRRRRKSSDFRGAVEKEPLGDVQTEDVFSDGEAGKDNAWS
SSQIAEREDSKDGTLSNLCSALESQNIESDSVLTFTQVNKVIDNLEQDSSPPSDTKSAYSSVCP
TGRISFKLYDWNPAEFPRRLRHQIFQWLASMPVELEGYIRPGCTILTIFVSMPQYMWVKLFD
DPVSYVQSSVGVGGILSGRGAALVYLNDTSFRIMREGASVMKVKVAVRTPKLHYVYPPCFE
AGKPMDLLACGSNLLQPKFRSLISFAGKYLAHDYYVAFPRGKGEDKPTIDHDRQFCRIYVP
HTEPSYFGPAFVEVENECGLSNFIPILIGDEHVCSEIKMIHQKHDCSYCRKKLHCKSSGSSYG
TCEISCSRQAALSEFMLDVAWLLKRPCSEKLHCILTCSQIQRYNGLLKFLISNRSTSILEKILQS
LQIVFDEVDSCDVTNDDTDIDRRLLKEHIVHAKDFLRQSAHKKELILHQAEGCIPKVDKCL
GSFAHDDLLPVDRTACQVTEMRVDNNCTVLEYSNSQNEGESISLINGEVTMNVKDNREWA
RKSCNNVISKKFVNTRPLVYMIAVVAVCFGVCAVVLHPYKVTKIAVTIRRCVFDNT*
