# Supplementary material for: Genome-Wide Identification and Characterization of SPL Family Genes in Chenopodium quinoa
Source: Genes (Basel). 2022 Aug 16;13(8):1455. doi: 10.3390/genes13081455 (PMC9408038; doi:10.3390/genes13081455)
Supplement: Supplementary file 1 [file genes-13-01455-s001.zip › Table S1-S3.pdf]

Table S1. List of species where the genome-wide identification of *SPL* genes was performed.

Species	No. of *SPL* genes	References
Arabidopsis thaliana	16	Cardon et al., 1999
Oryza sativa	19	Xie et al., 2006
Physcomitrella patens	13	Riese et al., 2007
Vitis vinifera	19	Wang et al., 2010
Solanum lycopersicum	15	Salinas et al., 2012
Malus domestica	27	Li et al., 2013
Vitis vinifera	18	Hou et al., 2013
Cucumis melo	13	Ma et al., 2014
Populus trichocarpa	28	Li and Lu, 2014
Ricinus communis	15	Zhang and Ling, 2014
Salvia miltiorrhiza	15	Zhang et al., 2014
Brassica rapa	29	Tan et al., 2015
Citrus Clementina	15	Shalom et al., 2015
Gossypium hirsutum	24	Zhang et al., 2015
Prunus mume	15	Xu et al., 2015
Triticum aestivum	58	Wang et al., 2015
Arachis hypogaea	15	Li et al., 2016
Brassica napus	58	Cheng et al., 2016
Capsicum annuum	15	Zhang et al., 2016
Chrysanthemum morifolium	12	Song et al., 2016
Nicotiana tabacum	15	Han et al., 2016
Panicum virgatum	35	Wu et al., 2016
Sorghum bicolor	18	Chang et al., 2016
Zea mays	31	Mao et al., 2016; Zhang et al., 2016
Ananas comosus	16	Ali et al., 2017
Betula platyphylla	12	Ning et al., 2017
Phyllostachys edulis	32	Pan et al., 2017
Solanum tuberosum	15	Kavas et al., 2017
Ziziphus jujuba	18	Shao et al., 2017
Ziziphus jujuba	16	Song et al., 2017
Glycine max	41	Tripathi et al., 2017
Betula luminifera	18	Li et al., 2018
Camellia sinensis	20	Wang et al., 2018
Fragaria vesca	14	Xiong et al., 2018
Gossypium arboreum	29	Cai et al., 2018
Gossypium raimondii,	30	Cai et al., 2018
Gossypium barbadense	59	Cai et al., 2018
Gossypium hirsutum	59	Cai et al., 2018
Petunia axillaris	21	Zhou et al., 2018
Brassica juncea	59	Gao et al., 2019
Citrus Clementina	15	Zeng et al., 2019
Fagopyrum tataricum	24	Liu et al., 2019

Medicago truncatula	23	Wang et al., 2019
Tamarix chinensis	14	Wang et al., 2019
Brachypodium distachyon	18	Tripathi et., 2020
Camellia sinensis	25	Zhang et al., 2020
Carica papaya	14	Xu et al., 2020
Hordeum vulgare	14	Tong et al., 2020
Jatropha curcas	15	Yu et al., 2020
Juglans regia	48	Zhou et al., 2020
Paeonia sufruticosa	16	Wang et al., 2020
Triticum aestivum	56	Zhu et al., 2020
Triticum aestivum	48	Li et al., 2020
Carya illinoinensis	32	Wang et al., 2021
Citrus sinensis	15	Song et al., 2021
Cucumis sativas	15	You et al., 2021
Dactylis glomerata	17	Feng et al., 2021
Piper nigrum	34	Li et al., 2021
Punica granatum	15	Li et al., 2021
Saccharum spontaneum	17	Feng et al., 2021; Liu et al., 2021
Vaccinium corymbosum	20	Xie et al., 2021
Ipomoea batatas	29	Sun et al., 2022
Morus alba	15	Li et al., 2022
Medicago sativa	22	Ma et al., 2022
Populus euphratica	33	Qin et al., 2022

Table S2. List of primers used in this study.

Primer name	Gene	Sequence（5'→3'）	Purpose
CH-S-01	*CqSPL1*	TGCATCCATTGCCTCTAGGT	qRT-PCR
CH-A-02		TTCGGTGCTTTGAACAACTG	
CH-S-03	*CqSPL2*	CCATTTCATCGGAGTTCTGG	
CH-A-04		CTCGTTTGGTGCTTTGAACA	
CH-S-05	*CqSPL3*	GTTTATTGTTCGGCGGTGAT	
CH-A-06		ATTTAATGGAGCGGCAAGAA	
ZM-S-01	*CqSPL4*	TGCCTGAACTACAGCAAGGA	
ZM-A-02		GGATGGAAAATGCCCGGTTG	
CH-S-09	*CqSPL5*	ATGAAGTTGGGTGAGCAGGT	
CH-A-10		TGGGAAGTTCCAGTTCCAAG	
ZM-S-03	*CqSPL6*	CGAAGCAAAGCGTTACCACC	
ZM-A-04		TGAGCTTGATCCCCTGTCCT	
CH-S-13	*CqSPL7*	ATGATGCCAGTTGTGGTTCA	
CH-A-14		TCTTGGACATGGTCCATCAG	
CH-S-15	*CqSPL8A*	TCAGGGGAAAATGCTCAATC	
CH-A-16		TACCCTTGGTGGTGATACCG	
CH-S-17	*CqSPL8B*	TCAGGGGAAAATGCTCAATC	
CH-A-18		ACACCCTTGGCGGTGATACT	
CH-S-19	*CqSPL9*	CTACATGAACGTTGAAGGGTCT	
CH-A-20		CGCTACCCACATCCTCAAA	
CH-S-21	*CqSPL10*	CACACCAATCAAGTCCACCA	
CH-A-22		CGCAGCATAATCGGAAAGTC	
CH-S-23	*CqSPL11*	CTTGCTCTCAGGGTTTGGAC	
CH-A-24		CCAACCAAATGGGAAAATCC	
CH-S-25	*CqSPL12*	TGGGAACATAAATCCCCTCTT	
CH-A-26		CACTTCTGCAATACCCAATTCA	
CH-S-27	*CqSPL13*	TGGGGATTTCATATGTGGTG	
CH-A-28		TTGCATCAGATTGAGAGATGG	
CH-S-29	*CqSPL14*	CAGCTCAAGGTAGCAGCACTT	
CH-A-30		GGAGCATTGGTGTTGGAACT	
CH-S-31	*CqSPL15*	GACTCTGATGTCATCACTGA	
CH-A-32		CTAGACTCCGCTTCAACAA	
CH-S-33	*CqSPL16*	CCGTTGTGAGTGGACAAGAC	
CH-A-34		TGACGAGTACCCACCCATGT	
ZM-S-05	*CqSPL17*	CGTAGAAGGAAGCCTCAGCC	
ZM-A-06		GCTGTTGTAGAGTGTCGGGT	
ZM-S-07	*CqSPL18*	GAGGGGAAGCGGAGTTGTAG	
ZM-A-08		AAGGGAAGTTGAGGGTCAGG	
ZM-S-09	*CqSPL19*	TTCCTCGGTTGAGAGGAGGT	
ZM-A-10		TGCTTTCCCACACACAATGC	
CH-S-41	*CqSPL20*	GACCCACCATGATCAATCCTA	
CH-A-42		ATACCACGTTCCTTGGGATG	

ZM-S-11	*CqSPL21*	CTTGAAGCAGGGTGGGGAAT
ZM-A-12		GAAAGGCCGAGTACTGGTGA
CH-S-45	*CqSPL22*	ATGATTTTCTTCGCCAAAGC
CH-A-46		TGTGTGGTTCGTCTCTGCTC
LT-S-CqACT2-QRT	*CqACT2*	CCCCTGCTATGTATGTTGCAATTC
LT-A-CgACT2-QRT		AGTGGTCTGTTAGGTCACGACCAG
LT-S-CqRAN3	*CqRAN3*	TTGGTGTCGAAGTTCATCCATTGG
LT-A-CqRAN3		GTAAGTCAATCGAGCAGTCACATC
SX-S-1664	*Cqu-MIR156a*	ACATAGCAAACCCAAACCAACAC
SX-A-1665		ACCAATAATCTTCAACCCCAACTC
SX-S-1666	*Cqu-MIR156b*	TTCGAATGGTAAGAGGGAGGTGAC
SX-A-1667		AGACAGACAGAGAGTAAGCACG
SX-S-1749	*Cqu-MIR156c*	ATGGGTGGGAGGAGTGAGAAATTG
SX-A-1750		TGGAGTTGGAAGGGAAGCTGACAG
SX-S-1670	*Cqu-MIR156d*	AATGAATGGTAAGAGGGAGGTG
SX-A-1671		AAGCACGCATAGCTTCAAGCATAAG
SX-S-1674	*Cqu-MIR157a*	GAGGCAGTGGTGAATGGAAGCTG
SX-A-1675		AGGTGGTGACAGAACATAGAGAG
SX-S-1680	*Cqu-MIR157j*	GCGGCAGTGGTGAATGGAAGCTG
SX-A-1681		AGAGAGGGGCTGAAGGTGATGAC
SX-S-1682	*Cqu-MIR157k*	TGCATTGGGATATTTGGATTTGG
SX-A-1683		AGAGAGGCAGGAACTGATGATGAC
SX-S-1684	*Cqu-MIR157l*	ATGAAGTGCATGGGGATATTTGG
SX-A-1685		ACGAAGGAAAGTGAAGTGCAGCAG

Table S3. Categories of cis-elements presented in the promoters of *CqSPL* genes.

**Category**	**Cis-elements**
Light responsiveness	3-AF1 binding site, ACE, AE-box, AT1-motif, ATC-motif, ATCT-motif, Box 4, Box II, chs-CMA1a, chs-CMA2a, chs-Unit 1 m1, GA-motif, Gap-box, G-box, GATA-motif, GT1-motif, GTGGC-motif, I-box, LAMP-element, MRE, Sp1, TCCC-motif, TCT-motif
Development	AC-II, AP-1, as-1, AAGAA-motif, CARE, CAT-box, CCGTCC-box, circadian, dOCT, F-box, GCN4_motif, HD-Zip 1, HD-Zip 3, MBSI, MSA-like, O2-site, re2f-1, Telo-box
Hormone responsiveness	ABRE, ABRE2, ABRE3a, ABRE4, AuxRR-core, CGTCA-motif, ERE, GARE-motif, P-box, TATC-box, TCA-element, TGACG-element, TGA-element
Abiotic stress	ARE, AT-rich sequence, CCAAT-box, DRE, DRE core, DRE1, LTR, MBS, MBSI, MYB, MYB recognition site, Myb-binding site, MYC, STRE, TC-rich repeat
Biotic stress	box S, W-box, WRE3, WUN-motif
Promoter-related	A-box, AT-rich element, AT-TATA-box, CAAT-box, CCAAT-box, TATA, TATA-box, unnamed_1
Unknown function	Box III, CCGTCC motif, CTAG-motif, MYB_like sequence, TCA, unnamed__2, unnamed__4, unnamed__6, unnamed__8, unnamed_10, unnamed__12, unnamed__1 4, Y-box
